# Neuropathogenesis-on-chips for neurodegenerative diseases

**DOI:** 10.1038/s41467-024-46554-8

**Published:** 2024-03-12

**Authors:** Sarnai Amartumur, Huong Nguyen, Thuy Huynh, Testaverde S. Kim, Ran-Sook Woo, Eungseok Oh, Kyeong Kyu Kim, Luke P. Lee, Chaejeong Heo

**Affiliations:** 1 Department of Biophysics, Institute of Quantum Biophysics, Sungkyunkwan University, Suwon, 16419 Korea; 2 Center for Integrated Nanostructure Physics (CINAP), Institute for Basic Science (IBS), Suwon, 16419 Korea; 3 Department of Anatomy and Neuroscience, College of Medicine, Eulji University, Daejeon, 34824 Korea; 4 Department of Neurology, Chungnam National University Hospital, Daejeon, 35015 Korea; 5 Department of Precision Medicine, Graduate School of Basic Medical Science (GSBMS), Institute for Anti-microbial Resistance Research and Therapeutics, Sungkyunkwan University School of Medicine, Suwon, 16419 Korea; 6 Harvard Medical School, Division of Engineering in Medicine and Renal Division, Department of Medicine, Brigham and Women’s Hospital, Boston, MA 02115 USA; 7 Department of Bioengineering, Department of Electrical Engineering and Computer Science, University of California, Berkeley, CA 94720 USA

**Keywords:** Alzheimer's disease, Huntington's disease, Amyotrophic lateral sclerosis, Parkinson's disease, Neurodegeneration

## Abstract

Developing diagnostics and treatments for neurodegenerative diseases (NDs) is challenging due to multifactorial pathogenesis that progresses gradually. Advanced in vitro systems that recapitulate patient-like pathophysiology are emerging as alternatives to conventional animal-based models. In this review, we explore the interconnected pathogenic features of different types of ND, discuss the general strategy to modelling NDs using a microfluidic chip, and introduce the organoid-on-a-chip as the next advanced relevant model. Lastly, we overview how these models are being applied in academic and industrial drug development. The integration of microfluidic chips, stem cells, and biotechnological devices promises to provide valuable insights for biomedical research and developing diagnostic and therapeutic solutions for NDs.

## Abstract (web summary)

This review focuses on recent advances in on-chip platforms for patient-like in vitro modeling of the pathology of neurodegenerative diseases, including Alzheimer’s, Parkinson’s, and Huntington’s diseases as well as Amyotrophic lateral sclerosis. The authors advocate for broader usage of these human-relevant models in the academic and pharmaceutical fields.

## Introduction

As life expectancy increases with advances in medical care, humanity faces a new crisis related to the rising ageing population^1^. This leads to a concomitant increase in the incidence of incurable neurodegenerative diseases (NDs)^2–4^. These diseases generally affect the brain activity in the elderly by diminishing their cognitive and behavioural functions^5–8^. Tremendous efforts have been made for decades to investigate the underlying mechanisms of these fatal NDs using both in vivo and in vitro systems, despite the complexity of the human brain structure and the limitations of real-time observation. Notably, several pathogenic features, such as specific neuronal loss, gliosis, neuroinflammation, oxidative stress, mitochondrial dysfunction, and early vascular damage tend to overlap in common NDs, including Alzheimer’s disease (AD)^9,10^, Parkinson’s disease (PD)^6,11^, amyotrophic lateral sclerosis (ALS)^7,12^, and Huntington’s disease (HD)^13,14^. Although the primary initiating factor remains elusive, hypotheses suggest that the accumulation of several dysfunctional proteins is closely related to the onset of each ND^15^.

Despite advances in our understanding of NDs, the development of disease-modifying treatments (DMT) that target the underlying mechanisms of their pathogenesis remains challenging. Only a few DMTs, such as aducanumab^16^ and lecanemab^17^ for AD and riluzole^18^ and edaravone^19^ for ALS, have recently been approved. A substantial number of drug trials for NDs that were effective in preclinical studies have yet to demonstrate efficacy in clinical trials, and the limitations of current experimental models are responsible for this failure. Notably, animal models, which have been heavily relied upon for the drug development of NDs, differ fundamentally from humans in terms of their immune systems^20^ and the ratio, distribution, morphology, and gene expression of their brain cells^21^. Moreover, current animal models of NDs cannot fully replicate the complex pathogenic lesions of human NDs, and often overlook sporadic cases, which account for most cases^22,23^. To investigate the pathological processes of NDs, advanced in vitro models that can accurately replicate various physiological conditions, multicell types, and interactive cell-to-cell environments, and provide real-time monitoring modulated by different biochemical and biophysical cues are in high demand.

Over the past decades, stem cell biology and advances in cell culture techniques have rapidly developed a stem cell-based 3D culture platform that generates an “organoid” for disease modelling and drug screening^24^. Organoids have several advantages over conventional 2D cell cultures, including patient-specific origins and intrinsically complicated 3D compositions that mimic tissues. Several brain organoid subregions have been developed for brain studies, including the cerebral cortex^25^, hippocampus^26^, hypothalamus^27^, forebrain^27^, midbrain^27,28^, functional choroid plexus^29^, and cerebellum^30^. However, current methods for organoid growth have several limitations that need to be addressed, including insufficient neuronal functionality and cellular microenvironment, lack of vascular and immunological components, low reproductivity and uniformity, and difficulties in long-term maintenance^31–33^.

Microfluidic organs or organoids-on-chips have provided unique opportunities for researchers to develop an inventive experimental design by allowing the reproduction of critical elements of each organ, such as disease-specific miniaturised environments with high controllability and tunability, biochemical, and biophysical cues, continuous medium flow, and complex interactions between cells relevant to the in vivo system^34^. Moreover, integrated microfluidic chips are ideal platforms for drug screening and basic life science studies in biomedical research^35^. Despite the challenges in determining the brain’s critical structural and functional units owing to its complex morphology and physiology, many commercialised and customised microfluidic chips have simplified the complex characteristics of the brain into miniaturised systems^22^. Existing brain-on-chips mimic diverse levels of brain units, such as the axonal chip^36^, neuron-glia chip^37^, blood-brain-on-a-chip^38,39^, and neurovascular-unit-on-a-chip^40^. These have been applied in studies on brain tumours^41^, vascular diseases^40^, brain injury^42^, neuroinflammation^43^, and NDs^44^. These microfluidic chip designs can be repurposed for desired disease conditions according to the experimental targets, allowing researchers to understand neuropathogenesis at a more profound level.

Recently, the idea of taking advantage of microfluidic chips to overcome the limitations of organoid formation was proposed, with promising results in the development of organoids-on-chips for the pancreas^45^, liver^46^, kidney^47^, stomach^48^, and brain^49–51^. This approach provides better in vivo mimicry properties than conventional models, preventing necrosis by controlling organoid expansion with chip size, integration with the vascular bed to provide sufficient nutrients, allowing co-culturing with systemic microglia, applying mechanical and physical cues, delivering a drug through a physiologically relevant barrier, and achieving mature characterisation with continuous fluid flow. However, there is still room for improvement in both culture systems to accelerate their application in drug screening and personalised medicine. In this review, we aim to highlight the potential of integrated microfluidic technologies for advancing research on the neuropathogenesis of NDs (AD, PD, ALS, and HD) to make substantial breakthroughs in disease modelling and drug development.

## Current understanding of neurodegenerative diseases

**Fig. 1:**
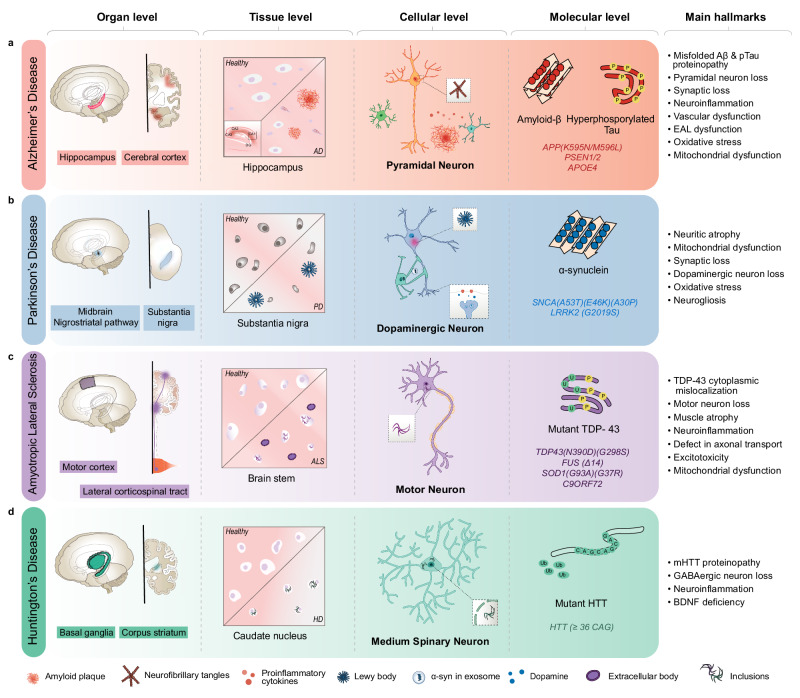
The similarities and differences in the main hallmarks of neurodegenerative diseases from the organ to molecular levels. **a** AD is characterised by the inclusion of misfolded amyloid-β (Aβ) and neurofibrillary tangles in pyramidal neurons, primarily in the hippocampus and cortex regions of the brain. **b** PD is characterised by Lewy body aggregates composed of misfolded α-synuclein and degeneration of dopaminergic neurons in the substantia nigra region of the brain. **c** ALS is characterised by including mutant TAR DNA-binding protein 43 (TDP-43) and other proteins, degeneration of motor neurons in the motor cortex and spinal cord, and muscle atrophy with dysfunctional proteins. **d** HD is characterized by including mutant Huntingtin protein (mHTT) and degeneration of medium spiny neurons in the basal ganglia, and corpus striatum of the brain. AD Alzheimer’s disease, ALS amyotrophic lateral sclerosis, BDNF brain-derived neurotrophic factor, EAL endosomal-autophagic-lysosomal pathway, GABA gamma-aminobutyric acid, HD Huntington’s disease, PSEN presenilin 1, SNCA synuclein alpha.

Numerous studies have identified the interconnected pathogenic features that contribute to ND progression over several decades. These include dysfunctional protein-related pathogeneses, gliosis, neuroinflammation, metabolic alterations, oxidative stress, mitochondrial dysfunction, and genetic alterations. However, the primary aetiologies of selective neuronal cell death and synaptic loss in NDs remain to be determined. This has hindered the development of disease-modifying drugs and early diagnostic tools. In this section, we review the current progress in the investigation of AD, PD, HD, and ALS. These diseases share some commonalities and distinctions in their pathogeneses (Fig. 1).

### Alzheimer’s disease

AD has become a major concern worldwide, accounting for 80% of all dementia cases. The prevalence of AD is expected to rapidly increase over the next 30 years^52^. In AD, symptoms of cognitive and behavioural impairment occur as the first, but memory loss presents much earlier, before clinical diagnosis, especially in the predominant age group (>65 years)^53,54^. This results from progressive neuronal and synaptic loss mainly in the cortex and hippocampus (Fig. 1a). Unfortunately, when AD is first diagnosed in the clinical setting, several irreversible pathological changes occurring in the brain are already in progress^55^. One of the main pathological characteristics of AD is the deposition of amyloid plaques and neurofibrillary tangles (NFT) in the brain tissue. On a macroscale, moderate atrophy of the cortex, amygdala, and hippocampus as well as enlargement of the temporal horn of the lateral ventricle have been observed in AD. This ultimately leads to a loss of whole brain volume^56^. Gene mutations linked to amyloid protein processing pathways, such as *APP*^57^*, PSEN1*^58^, and *PSEN2*^59^ are responsible for early-onset AD (EOAD). Recent genome-wide association studies (GWAS) have revealed many genetic factors associated with late-onset AD (LOAD), such as *BIN1, APH1B, PTK2B, PILRA, CASS4, CCDC6, TSPAN14, NCK2*, and *SPRED2*^60,61^. However, most LOAD cases are attributed to *APOE4*^62^ and *TREM2 R47H* variants^63,64^, which regulate brain inflammation, cholesterol, and glucose metabolism, and microglia function^65,66^. In this section, we describe the current leading hypotheses of AD, such as amyloid-β (Aβ) and tau protein dysfunction, neuroinflammation, blood-brain barrier (BBB) dysfunction, gut-brain axis, mitochondrial dysfunction, oxidative stress, and metabolism alterations.

#### Amyloid-β and hyperphosphorylated tau-related pathogenesis

The most prominent histological hallmark of AD is the deposition of misfolded amyloid aggregates or plaques in the extracellular space. The core element of amyloid plaques is the pathological Aβ peptide (~4 kDa), which is produced by altered cleavage of the amyloid precursor protein (APP). APP is a type I transmembrane protein that is typically involved in cell interactions, migration, synapse formation, and neuroprotection. It is mainly produced by neurons, astrocytes, platelets, and vascular endothelial cells^67,68^. However, in the familial AD brain, the APP cascade is altered due to mutation in protein processing genes (*APP, PSEN1*, and *PSEN2*), causing the formation of Aβ peptides^67,68^. Among Aβ peptides of various lengths, Aβ42 tends to aggregate and form plaques, but Aβ40 is the most abundant. In addition, this abnormal aggregation is induced by the impairment of protein regulation systems, such as the endoplasmic reticulum system, ubiquitin-proteasome system (UPS), and autophagy-lysosomal pathway (ALP)^69^. Importantly, dysfunction of several regulating proteins, such as LRP1^70^, SNX6^71^, GGA3^72^, and SORLA^73^ affect APP endocytosis, endosomal trafficking, and Aβ production, which are closely associated with the onset of AD.

Studies have focused on examining differences in the effects of various forms of Aβ on cells, and they reported that intermediate forms of Aβ, such as oligomers and protofibrils, are more neurotoxic than mature fibrils^10,74,75^. Several in vitro studies have reported that the pathogenicity of oligomers varies depending on their size and structure. These oligomers are known to cause neurotoxicity by binding to neuronal receptors (PrPc^76^, NMDAR^77^, β2-AR^78^, p75NTR^79^, and α7nACR^80^), altering cell membranes^81^, causing mitochondrial dysfunction^82^, Ca^+2^ imbalance^83^, as well as inducing tauopathy^84^.

AD progression may be linked to Aβ propagation among neurons. In the human AD postmortem models, the positions of Aβ plaques exhibit a regional-specific outer to inner pattern, initially detected at the frontal, and medial parietal cortex, followed by the allocortical region and midbrain. Then, in late clinical phase, Aβ accumulation is found in the brainstem and cerebellum^85^. Furthermore, positron emission topography (PET) imaging of the AD brain has revealed that Aβ dynamically accumulates in synaptic contact areas, which may be a result of Aβ propagation^86^. Also, an in vitro study has demonstrated prion-like propagation of Aβ oligomers between neighbouring cells^87,88^, which is explained by the “seeding-nucleation” theory observed in an in vitro study as a three-step process (seeding, nucleation, and elongation), where Aβ oligomers formed from monomers elongate into fibrils. These fibrils then accelerate the formation of oligomers (nuclei), resulting in increased aggregations^89,90^. Moreover, current hypotheses have shown three Aβ propagation ways: direct cell contact^91^, exosomes^92–94^, and tunnelling nanotubes between cells^95–97^.

Abnormal intracellular accumulation of tau proteins, NFTs, is another primary pathological marker that is considered to be more closely related to cognitive decline^98^. Tau, a microtubule-associated protein, is mainly located in the axons and supports axonal transport, maintenance, and generation of microtubules. However, alterations in post-translational modification promote its aberrant aggregation^99^. Among the several types of post-translational modifications, the relationship between *hyperphosphorylated tau* (pTau) and AD pathogenesis has been well studied by discovering several phosphorylation sites (Thr175, Ser202, Thr205, Thr212, and Ser422) and its toxic promotion of misfolding and self-aggregation^100^. Furthermore, other neuroprotective phosphorylated states (Ser214, Ser262, and Ser305) inhibit tau aggregation^101,102^. However, hyperphosphorylation depends on the starting site, which may accelerate other multisite phosphorylation states^103^. The leading cause of tau hyperphosphorylation is unknown despite the discovery of several tau-associated kinases and phosphatases, such as GSK-3β, CDK5, ERK2, PKA, and PP2A^104–107^. Recently, one group found that a 12-amino-acids-long peptide derived from CDK5 disrupts hyperactivated CDK5 (CDK5-p25 complex). This enzyme leads to decreased DNA disruption, pTau levels, and neurodegeneration, and increased behavioural ability in an AD mouse model^108^.

The spread of toxic tau shows similar characteristics to Aβ, such as prion-like behaviour and propagation between cells via direct contact^109^, exosomes^110^, and nanotubules^111^. In vivo and in vitro studies have shown that tau propagation from the entorhinal cortex (EC) to the hippocampal region is mediated by exosomes released from microglia^112^. Similarly, one in vitro study has also shown that microglia secreted extracellular vesicles (EVs) containing Aβ propagate through axons, causing synaptic alterations^113^. Despite the protective effect of microglia exosomes and EVs by promoting clearance of Aβ and tau, studies suggest that these may be a factor in propagation among cell-cell interactions in the AD brain, which makes them an exciting aim for further research.

The relation between Aβ and NFT in AD pathogenesis remains controversial. In the early phase, NFTs were observed in neurons of the more profound parts of the EC and CA1 regions of the hippocampus, followed by other parts of the brain, including the parietal, medial, and lateral occipital cortices, in a reversed pattern of amyloid plaques^86^. A histological-level study also found that NFT begins to form and spread under the influence of Aβ deposition^114^. Recent automatic PET imaging analysis has revealed that tau accumulation first appears at the rhinal cortex, independent from Aβ^115^. Subsequently, tau spreads robustly throughout neocortex when the Aβ level reaches at certain threshold. This finding has been termed ‘Ca-tau-strophe’ and highlights the importance of the tau protein in earlier diagnosis and therapeutic choice^115,116^. However, the detailed mechanisms underlying tau protein toxicity have not been fully elucidated. However, substantial research findings have proposed an association between Aβ and other proteins (such as Pin1, HSP, Fyn kinase, α-synuclein, and PASCIN1) regarding tau hyperphosphorylation^117,118^. Aβ accumulation induces tau aggregation and propagation trans-synaptically in vitro studies^119^. Notably, some studies have suggested that NFT may protect against amyloid plaques^117^. This emerging evidence has indicated the importance of synergy of Aβ and tau in AD pathogenesis^120^.

APP-C99, a C-terminal fragment of APP, has recently been suggested as an early AD marker associated with endosomal-autophagic-lysosomal (EAL) malfunction^121^. C99 accumulation in neurons causes lysosomal and synaptic distortion and cognitive changes by aggregating within EAL vesicle membranes^122^.

#### Neuroinflammation and systematic inflammation

Compelling evidence for the association of immune-regulating genes with AD suggests that systemic immune-mediated neurodegeneration is an alternative hypothesis for the pathogenesis of AD. As ageing, circulating proinflammatory cytokines (IL-1β, IL-6, TNFα, and IL-18) induced by infection, chronic diseases, stresses, and cellular senescence may initiate or contribute to the neuropathogenesis of AD^123^. When these cytokines cross the BBB, they can activate glia and astrocytes, leading to Aβ and tau phosphorylation, oligomerization, neuroinflammation, and neurotransmitter toxicity^123^. In pathological studies, increased levels of proinflammatory proteins in the cerebrospinal fluid (CSF) have been associated with brain volume loss and thinning of the white matter, corresponding to cognitive dysfunction^124^.

As Aβ plaques form, the presence of activated microglia and astrocytes also increase^125^. Therefore, extensive research has focused on the role of astrocytes and microglia in AD progression, including their contribution to Aβ and pTau depositions, neuroinflammation, and Ca^+2^ imbalance. Microglia exert their neuroprotective activities by clearing misfolded proteins^126^. However, as AD progresses, microglia transform into a deteriorating state, enhancing pTau-related kinase expression and production of the inflammasome NLRP3 (NOD-, LRR-, and pyrin domain-containing protein 3). Increased production of NLRP3 accelerates the activity of caspase-1 and secretion of proinflammatory cytokines, such as IL-1β and IL-18, and promotes neuronal and glial death via apoptosis^127,128^. In addition, GWAS identified multiple genes associated with LOAD (*CR1, SPI1, MS4As, TREM2, ABCA7, CD33*, and *INPP5D*) that are expressed in microglia, underscoring the important role of microglia in AD pathogenesis^129^. Specifically, studies have suggested that a mutation of TREM2, lipid sensing transmembrane glycoprotein expressed in microglia, causes the loss of its phagocytosis function^130^, and diminishes its migration towards Aβ plaques and uptake of Aβ_40_ and Aβ_42_^131,132^. Notably, the observation of APOE as a ligand for TREM2 has revealed new insights into their association with the pathogenesis on Aβ and pTau^133,134^. Yeh et al. ^134^ demonstrated that microglia expressing TREM2 variants exhibited reduced internalisation of Aβ and the apolipoprotein or lipoprotein complex. Gratuze et al. ^135^ recently suggested that microglial activation could worsen tau-associated neurodegeneration in the presence of APOE4 through a TREM2-independent pathway. One group recently developed a successful therapeutic approach based on TREM2 function in microglia activation. They introduced a high-affinity human TREM2 antibody with a monovalent transferrin receptor (TfR) binding site to enable efficient delivery to the brain. This antibody promotes glucose and lipid metabolism in microglia, shifting them to a metabolically responsive state distinct from that triggered by amyloid pathology^136^.

Reactive astrocytes play a critical role in the progression of AD. It has been suggested that astrocytes become reactive to surrounding plaques, but there are limited data on the exact morphological and functional alteration^137,138^. A recent PET study revealed a correlation between reactive astrocytes and protein biomarkers (Aβ, and tau) in the CSF of patients with AD, suggesting their contribution to progression^139^. The expression of APOE in many cell types has gained attention because APOE4 is considered the primary genetic risk factor for sporadic AD^140^. Astrocytes expressing APOE4 have demonstrated diminished functions, such as autophagy, clearance, and internalisation of Aβ, and support for neurons for their durability and synapse formation^141,142^. Moreover, a recent report suggests that astrocytes expressing APOE4 immoderately provide cholesterol to neurons by expanding their lipid membrane, increasing APP deposition, and promoting Aβ formation^143^. Conversely, oligodendrocytes expressing APOE4 exhibit dysfunction in myelination^144^, coinciding with increased neurotransmitter production and Aβ42 secretions in APOE4 neurons^145^. Studies have demonstrated an association between APOE4 expression and tauopathy. The deletion of APOE4 leads to the reversal of pathologies, including increased maintenance of the myelin sheath and decreased disease-associated neural subpopulations, and facilitates the severity of tauopathy^146^.

At the molecular level, using single-cell and single-nucleus RNA sequencing, researchers have recently revealed the specific transcriptional and functional characteristics of astrocytes and microglia, which have been categorised as disease-associated astrocytes (DAA) and microglia (DAM)^147,148^. The DAA subtype of astrocytes is associated with the early stages of AD and progressively increases over time. In AD mouse models, the level of homoeostatic astrocytes declines, while intermediate and glial fibrillary acidic protein highly expressed DAAs in the hippocampus and cortex region^147^. DAM showed the same transitioning pattern from a homoeostatic to an activated state. Moreover, one study indicated that phagocytic DAM subtypes can internalise Aβ proteins through a two-step sequential process that involves the downregulation of microglia-regulating genes and upregulation of phagocytic and lipid metabolism pathways^148^. According to a recent study using 3D in situ RNA sequencing, spatiotemporal variations in glial cells have been observed in the brains of AD mice. This study has revealed a layered position of cells surrounding Aβ plaques, with the first layer being DAM, followed by DAA and oligodendrocytes^149^.

#### Blood-brain barrier dysfunction

Research on the role of the BBB in AD is important because of the strong hypothesis of its association to the circulation of misfolded proteins originating from the brain or body system, and other pathogenic features. BBB disruption can be observed during the early stages of AD before regional atrophy occurs, and is emerging as a leading pathogenesis^150^. Initial defects in brain vessels may initiate various interconnected pathways such as misfolded proteins and neuroinflammation, and directly contribute to neurodegeneration^151^. Conversely, according to animal studies, pathological Aβ and tau proteins may cause disruption in blood vessels in vulnerable areas of AD during disease progression^152,153^. BBB disruption begins with changes in permeability, which are associated with a decrease in tight junction proteins in endothelial cells and reduced pericytes^151,154^. However, Aβ^155^ and tau accumulation^156^ disrupt the BBB integrity by altering the level of tight junction proteins. Moreover, microglia-driven neuroinflammation damages the BBB integrity and contribute to disease development^157^. Therefore, investigating brain vascular pathology is a promising avenue for gaining insight into the initial systemic mechanisms of all NDs.

#### Glymphatic system dysregulation

The dynamics of CSF and tissue interstitial fluid are supported by the perivascular network known as the glymphatic system^158^. Bidirectional aquaporin channels (AQP) facilitate continuous fluid flow to assist in circulating proteins and metabolites. The contribution of AQP to AD pathogenesis, associated with the clearance of pathological proteins, has been investigated^159,160^. In the absence of AQP4, *APP* mouse had decreased influx of CSF tracer, and accumulation of Aβ and APOE4 proteins in neurons, as well as phagocytic activation of microglia, suggesting the substantial role of AQP4 during disease progression^160^. Moreover, AQP4 knockout tau-expressing mice demonstrate increased tau aggregation and neurodegeneration, suggesting a highly relevant AQP4-dependent extracellular tau clearance pathway^161^.

AQP4 is primarily expressed in the endfeet processes of perivascular astrocytes in the BBB and tripartite synapses^162,163^. It is also found in the epithelial cells of the choroid plexus and the ependymal cells of the ventricular lining in the brain. Human postmortem AD models have shown decreased levels of perivascular AQP4 in the frontal cortex, which is associated with cognitive dysfunction and amyloid and tau pathology^164,165^. At the cellular level, the effect of mislocalisation of AQP4 channels on Aβ accumulation have been also investigated with deletion of α-syntrophin (SNTA1), important for perivascular localisation^166^. Several approaches have been proposed to inhibit AQP4 mislocalisation as a treatment for central nervous system (CNS) injury and stroke, with the aim of stabilising the BBB and disrupting the blood-spinal cord barrier disruption^167,168^. One group tested a calmodulin inhibitor targeting the mislocalisation of AQP4 to the calmodulin-mediated cell surface. This approach has successfully demonstrated reduced oedema and recovery of spinal cord injury. Moreover, the same calmodulin inhibitor decreases the expression of AQP4 proteins and reduces oedema, while increasing glycogen metabolism in the early acute phase in a post-stroke mouse model^168^. Cerebral oedema is the main hallmark of CNS injury and the main underlying factor for NDs, treating oedema by regulating AQP4 could be the treatment of choice for AD.

#### Gut-brain axis

The brain and gut communicate via neural, immune, endocrine, and metabolic pathways. Several hypotheses have proposed that the gut microbiome substantially contributes to brain disorders, including AD^169^. Considerable evidence suggests a connection between changes in gut microbiota and various features of AD, including Aβ, tauopathy, and neuroinflammation, and other pathogenesis-inducing neurodegeneration^170,171^. The current hypothesis is that gut microbiota indirectly affects BBB homoeostasis by activating peripheral immune cells. Alternatively, it can occur through the direct release of metabolites and hormones that travel through the vagus nerve, leading to neuroinflammation and contributing to proteinopathy^171^. Notably, a study has reported the antimicrobial effect of Aβ, revealing its protective role in immunity and inflammation^172^. This antimicrobial function has been consistent in all forms of Aβ peptide from oligomers to fibrils, not only in in vitro cultures, but also in AD mouse brains. Bacterial infection has accelerated the seeding and formation of Aβ deposition, which is in close proximity to the pathogen. These findings suggest that the inflammatory effects of infection and microbial changes may possibly initiate amyloid pathogenesis^172^.

A recent study demonstrated the impact of the gut microbiota on tauopathy of human APOE isoforms in a tau-transgenic mouse model^173^. In bacterial-free tauopathy mice, the level of pTau in the hippocampal area decreased, and microglia and astrocytes remained in a homoeostatic state in a sex- and APOE-dependent manner. In particular, when testing the differences in APOE isoforms, only APOE3 expressed in the tau male model showed improvement after treatment with antibiotics, but not the APOE4 isoforms of both sexes. Moreover, short-chain fatty acids in bacterial metabolites play a role in tau-induced neurodegeneration in mice. These findings strongly suggests that the importance of the gut-brain axis on AD pathogenesis, association with Aβ- and tau-induced pathogenesis, and neuroinflammation.

#### Brain-derived neurotrophic factor deficiency

Brain-derived neurotrophic factor (BDNF) is a critical factor in hippocampal neurogenesis that sustains synapses and neuronal circuits. A deficiency in BDNF is associated with several neuropathogenesis, including Aβ deposition, phosphorylated tau formation, neuroinflammation, and apoptotic cell death^174^. Moreover, the deficiency in neurogenesis observed in the early stages of AD can be explained by alterations in BDNF signalling; however, the underlying mechanism remains unknown. One study claimed that Aβ monomers maintain the production and release of BDNF by activating IGF receptors^175^. In addition, when BDNF was restored in a tau mouse model, behavioural changes, neuronal loss, and synaptic loss were reduced, but not hyperphosphorylation^176^.

#### Mitochondrial dysfunction and oxidative stress

In the early stages of AD, changes in mitochondrial structure, function, and accumulation have been observed^177^. Specifically, impairment of the mitophagy process is the major change currently attributed to cholesterol metabolism alterations related to APOE, mitophagy regulation of Aβ, tau protein, and APP-C99 accumulations on mitochondrial-associated membrane^177–179^. Astrocytes expressing APOE4 exhibit reduced fission and mitophagy^180^.

The principal characteristic of most NDs is the different vulnerable neurons^181^. During the progression of AD, neurons of the ECII, pyramidal neurons of the CA1, cholinergic neurons of the basal forebrain, noradrenergic neurons of the locus coeruleus, and rostral neurons are more vulnerable at the onset of the disease, whereas sensory cortical neurons begin to degenerate only at later stages. A recent study identified the molecular basis of vulnerable neuronal subtypes by profiling mRNA and successfully built a data source for modelling AD with specific neuronal circuits^182,183^. Vulnerability is attributed to oxidative stress, because oxygen-sensitive glutamatergic cells are selectively affected^184^. When oxidative stress increases, the vulnerable cells tend to experience DNA damage and mitochondrial fragmentation^185^.

#### Altered glucose and lipid metabolism

Understanding the complex roles of glucose and lipid metabolism is crucial for understanding neurodegeneration in the brain. According to a recent cohort study, high blood glucose and cholesterol levels increase the risk of AD^186^. Additionally, as the disease progresses, glucose levels in the brain decrease, whereas cholesterol levels increase^187,188^. Brain imaging studies have revealed that glucose metabolism is impaired in the hippocampal and cortical areas before the onset of cognitive decline or pathological changes^188,189^. Reduced glucose uptake is associated with accelerated neurodegeneration and Aβ depositions^189–191^. A four-month insulin treatment via the intranasal route improved cognitive function in APOE2- and APOE3-male mouse models, but not APOE4-expressed female mice^192^.

Others suggest that Aβ depositions cause a deficiency of GLUT-1 and GLUT-3 in vulnerable regions^193^. One group has proposed that BBB dysfunction, which is apparent at the onset of AD, is due to decreased GLUT-1 expression in BBB endothelial cells^194^. These results suggested a complex association between glucose metabolism and AD pathogenesis.

In the oldest investigation by Alois Alzheimer, lipid accumulation was highlighted as the main pathological feature in AD^195^. Subsequently, many high-risk genes associated with AD were found to be involved in lipid metabolism, including *APOE, TREM2 CLU*, and *ABCA7*. This suggests that lipids are essential for AD progression; however, the complex link between changes in lipid metabolism and AD pathology remains unclear. Cholesterol and sphingomyelin of lipid rafts regulate APP processing in lipid rafts, suggesting their role in the formation of Aβ^196,197^. During ageing, disruption of the lipid composition of the transmembrane membrane could be one of the earlier pathogeneses of LOAD. Furthermore, studies have indicated the role of glial cells in lipid metabolism, particularly cholesterol^143,144^. Astrocytes expressing APOE4 increase cholesterol input to neurons, causing an expansion of lipid rafts that ameliorate Aβ formation^143^. Meanwhile, oligodendrocytes expressing APOE4 show decreased myelinating function due to altered cholesterol metabolism^144^. Moreover, other studies have suggested a connection between lipid and glucose metabolism in AD owing to reduced glucose uptake caused by high levels of oxidised cholesterol^198^. These findings demonstrate that the initial pathogenesis of AD, particularly LOAD, may be related to disruption of metabolism in the brain.

### Parkinson’s disease

PD currently affects over six million patients worldwide and is the second most prevalent ND^164^. PD progresses slowly with complex clinical features, affecting not only the motor systems (tremor, bradykinesia, rigidity, and gait disturbance), but also non-motor systems (rapid eye movement sleep behaviour disorder), constipation, hyposmia, and cognitive impairment^199,200^. These symptoms correlate with neuronal degeneration in certain brain regions, most commonly in the substantia nigra pars compacta (SNpc), where dopamine neurons reside^201^ (Fig. 1b). Subsequently, patients begin to experience cognitive dysfunction in various extra-nigral regions, including the basal forebrain, thalamus, amygdala, and cortex, which initiates atrophy. Unfortunately, it is often diagnosed in a clinic much later, after the brain has lost approximately 50% of dopaminergic cell death^202^.

Most cases of PD are sporadic and occur later in life. Less than 10% of the cases related to genetic inheritance occur at an earlier age^203^. Currently, owing to GWAS, more than 200 genes associated with both early- and late-onset PD have been identified^204–207^. There were 90 loci related to familial cases, including *SNCA, LRRK2, VPS35, PRKN, PINK1, DJ-1* and *GBA*. Among these genes, point mutations and multiplications discovered in *SNCA* strongly intensified with the development of PD^208^. Other genes that govern important biological pathways in dopaminergic neurons, including mitochondrial function and protein degradation (*LRRK2, PARK2, PARK7, PINK1*, and *PRKN*), have also been suggested as significant risk factors for PD. Despite notable findings, the early pathogenesis of PD remains unclear because of its complexity. Here, we focus mostly on misfolded α-syn related pathogenesis, and dopaminergic neuron vulnerability, as well as mitochondrial dysfunction occurring in PD.

#### Accumulation of misfolded α-synuclein

One of the main conspicuous histopathological hallmarks of PD is the abnormal protein deposition called Lewy bodies (LBs) and Lewy neurites, which consist chiefly of aggregated α-syn^209^ and are found mostly in the extracellular space of SNpc^210^. A similar inclusion can be found in synucleinopathies, such as dementia with LBs, Parkinson’s disease dementia, and multiple system atrophy^211,212^. Physiological α-syn (14 kDa) is an intracellular protein, normally expressed by the *SNCA* gene, and is abundantly found in presynaptic terminal of neurons and erythrocytes. Although the physiological function of α-syn is not fully understood, several studies have suggested that it is associated with the modulation of synaptic functions^213^ and may have neuroprotective role^214^. The α-syn structure is generally divided into three regions: amphipathic domain (N terminus, residues 1–60), central non-amyloid component (NAC region, residues 61–95), and carboxyl acidic tails (C terminus, residues 96–140)^215^. The N terminus of α-syn consists of four 11-amino acid imperfect repeats and consensus sequence, KTKEGV, which is responsible for forming the α-helical structure when binding to the cellular membrane, such as apolipoproteins^216^. The hydrophobic NAC region is essential for forming β-sheet rich α-syn fibrils, which were first discovered in amyloid plaques and induce the formation of aggregated forms of α-syn^217^. However, the toxicity of LBs is still controversial, as some suggest that it is merely an accumulated trace of the cytoprotective response against PD progression^218^. Misfolded α-syn aggregation is considered the culprit of complicated pathogenesis of PD. Misfolded oligomeric α-syn is responsible for triggering neuronal toxicity by interfering with cell-to-cell signal transmission, mitochondrial pathways, and disruption of protein degradation pathways rather than mature fibrils^219–222^. Moreover, the physiological conformation of α-syn in vivo is an α-helical tetramer (58 kDa), rather than an unfolded α-helical monomer structure, that is resilient against aggregation^223,224^.

One of the notable theories of aggregation pathways is that misfolded α-syn aggregates may operate similarly to prions and self-propagate by converting normal prion proteins into abnormal ones through a seeding effect^87^. In the process of prion-like aggregation, α-syn creates a stable seed or nucleus as it combines into insoluble aggregates. This, in turn, accelerates formation of aggregated α-syn by misfolding α-syn and recruiting it into itself. The resulting aggregated α-syn can then be fragmented into small seeds, thereby perpetuating a vicious cycle of aggregation^225^. Preformed synthetic α-syn aggregates can accelerate the fibrillation of α-syn in buffer conditions^226,227^. Additionally, administrating preformed α-syn aggregates into mouse brains and cultured neurons facilitates the accumulation of α-syn aggregates in neurons, ultimately leading to the development of PD^228–230^. α-syn originates in the olfactory bulb or dorsal nucleus of the vagus nerve before propagating to other regions^15,231^. Notably, some groups have proposed the bottom-up hypothesis of prion-like progression of α-syn pathology, which suggests that the propagation is caused by the transmission through the autonomic nervous system^232^. This is supported by the earlier gastrointestinal symptoms prior to the onset of motor symptoms in patients with PD. This current research suggests that prion-like behaviour of α-syn may explain the progression of PD.

The factors that contribute to the development of abnormal α-syn aggregates are not yet fully understood. However, familial PD risk genes play a role in triggering aberrant aggregation. Specifically, multiplications (duplication and triplication) of the *SNCA* gene results in an overexpression of α-syn by disrupting the proteostasis process, including the synthesis, degradation, and clearance of α-syn^233,234^. In addition, several missense mutations in the *SNCA* gene, including A53T, A30P, E46K^235^ and H50Q^236^, disturb the folding dynamics of α-syn by distorting its intrinsic structure, which can lead to the early onset of PD^237,238^.

#### Dysfunction in protein degradation pathways

As LBs accumulate during PD progression, researchers have investigated protein degradation pathways to confirm the link between the impairment of these pathways and the onset of the disease. Under normal physiological conditions, protein dynamics are regulated via protein degradation pathways, such as UPS^239^ and ALP^240^. However, when these degradation pathways are disrupted owing to ageing, oxidative stress, inflammation, or other unknown causes, they can lead to the abnormal deposition of misfolded proteins in neurons, causing neuronal toxicity, which is related to various NDs^241,242^.

In PD, LBs are highly ubiquitinated and the cellular components of the UPS are abundantly identified, suggesting that protein clearance via the UPS is closely related to the pathogenesis of PD^243,244^. In vitro and in vivo studies have shown that introducing proteasomal inhibitors to disrupt the UPS led to accumulation of misfolded α-syn and neuronal death due to an impaired 26S proteasome pathway^245–247^. Conversely, overexpression of α-syn in dopaminergic neurons can cause dysfunction in the UPS by disrupting the 26S proteasome. This precedes the onset of behavioural impairment and neurodegeneration^248^.

Another protein degradation pathway, the ALP pathway, plays an important role in clearing large cellular debris that cannot be removed by the UPS. The ALP system functions through lysosomal acidification via three delivery pathways: macroautophagy, chaperone-mediated autophagy (CMA), and microautophagy. Perturbations of these lysosome-mediated protein degradation pathways are associated with pathogenesis of PD^249^. The introduction of genetic mutations (*ATP13A2* and *LAMP2*) into the components of lysosomal degradation pathways induces the accumulation of α-syn aggregates^250,251^. In contrast, overexpression, or modified forms of α-syn could impair the clearance of misfolded proteins by hampering the CMA-mediated protein degradation. This could eventually lead to an increase in the pathogenesis of α-syn^252,253^.

#### Degeneration of highly vulnerable dopaminergic neurons

Motor dysfunction in PD is explained by dopamine deficiency caused by the degeneration of dopaminergic neurons in the SNpc. Mainly dopaminergic cells are found to be the most vulnerable to pathogenic protein toxicity, leading to impaired dopaminergic signalling from the basal ganglia to the whole brain area, such as the nigrostriatal pathway. Corresponding to dopamine depletion, several pathological processes that regulate dopamine synthesis occur in the brain^254^. Compensatory overexpression of postsynaptic dopamine receptors occurs when there is a considerable loss of dopaminergic neurons^255^. Activated microglia are also observed on-site in dopaminergic neurons, promoting degeneration by releasing reactive oxygen species (ROS)^256^ and neurotoxic cytokines, and inducing glutamate excitotoxicity^257^. Furthermore, a deficiency in the dopaminergic circuitry is associated with reduced neurogenesis in the subventricular and subgranular zones^6,258^. However, molecular evidence for the selective vulnerability of dopaminergic neurons to degeneration is lacking. Recently, researchers profiled the genomics of PD-derived dopaminergic neurons and found that *AGTR1* expressed by a specific subgroup in the ventral tier of the pars compacta was highly upregulated in degeneration targets^259^.

#### Mitochondrial dysfunction

Mitochondria, which control cell death and energy production, have been linked to the pathogenesis of various NDs^260^. The chemical toxin 2-methyl-4-phenyl-1,2,3,6-tetrahydrophiridine (MPTP) was first found to cause acute Parkinsonism by disrupting mitochondrial pathways^261^. Once MPTP enters dopaminergic neurons through the dopamine transporter, it is oxidised by enzymes and converted into 1-methyl-4-phenylpyridinium ions (MPP^+^). It damages dopaminergic neurons by interfering with mitochondrial metabolism, specifically complex I of the electron transport chain, leading to increased oxidative stress^262,263^. As this environmental factor causes mitochondrial dysfunction, which is implicated in Parkinsonism, accumulating reports have suggested a substantial correlation between mitochondrial dysfunction and PD^264,265^.

α-syn plays a central role in pathogenesis of PD by inhibiting mitochondrial pathways^226^. One study identified α-syn null mice that were resistant to Parkinsonism caused by the administration of MPTP, implying that α-syn is essential for causing toxicity to dopaminergic neurons in conjunction with MPP^+^^266,267^. Additionally, α-syn aggregates negatively influence mitochondrial function, including mitochondrial fission and fusion, mitochondrial fragmentation, and complex I deficiency^268^. Exposure of preformed α-syn fibrils induces the recruitment of phosphorylated acetyl-CoA carboxylase 1 (ACC1) at the mitochondrial membranes, causing mitochondrial fragmentation. Oligomeric α-syn specifically compromises the mitochondrial complex I pathway and generates ROS in cybrid cell lines, leading to increased cell death^269^. Dopaminergic neurons generated from patients with PD carrying the A53T mutation or triplication of the *SNCA* gene show accumulated α-syn inclusions, as well as impaired cellular morphology and membrane potential^270^. In addition to impacts of environmental agents and misfolded α-syn on mitochondrial dysfunction in PD, GWAS have discovered that various genetic variants related to mitochondria are implicated in the early onset of PD^271,272^. Among the genetic factors, autosomal recessive *PARK2* and *PARK6*, which express E3 ubiquitin ligase (Parkin), and PTEN-induced kinase 1 (PINK1), respectively, are strongly associated with mitochondrial dysfunction. Under normal conditions, PINK1 and Parkin are associated with the mitochondrial quality control system, mitophagy, which degrades and recycles damaged mitochondria to maintain mitochondrial homoeostasis^273^. However, when genetic mutations occur in these genes, the PINK1/Parkin system is unable to prevent oxidative stress and an imbalance in ATP production in mitochondria, eventually leading to the degeneration of dopaminergic neurons^274–276^.

The impairment of DJ-1, which protects neurons from oxidative stress by acting as a redox sensor and antioxidant, is also associated with the pathogenesis of PD, particularly in familial cases^277,278^. In PD, due to DJ-1 dysfunction, ROS production increases in the mitochondria, causing damage to neurons. In fact, DJ-1 is localised in the mitochondria and overexpression of DJ-1 relieves oxidative stress, preventing neuronal death^279–282^. However, when genetic mutations of the *DJ-1* gene disrupt its biological pathways, which can lead to neurodegeneration and a monogenic form of Parkinsonism by compromising antioxidant activity^277^.

#### Environmental factors and neurotoxicity

Given the increasing impact of environmental pollution, it is crucial to consider the influence of neurotoxic chemicals on the onset and development of age-related NDs. Neurotoxic heavy metals, pesticides, and metal-based nanoparticles contribute to ND pathogenesis^283^. Chronic exposure to lead, the main component of polluted air, is associated with cognitive dysfunction^284^. Common neurotoxic mechanisms have been observed in BBB disruption, oxidative stress, mitochondrial dysfunction, and protein aggregation during ND progression^285^.

Liu et al. ^286^ recently reported that polystyrene nanoplastic particles induce the α-syn mediated neurodegeneration by invading the brain through BBB. They hypothesised that charged nanoplastics disrupt the lysosome degrading function, and trigger the α-syn protein misfolding, resulting in the intracellular α-syn fibril accumulation in neurons. This study has demonstrated that there is a notable area to investigate concerning the detrimental effects of nanoplastics on the other NDs.

### Amyotrophic lateral sclerosis

Amyotrophic lateral sclerosis is a chronic, progressive disorder that specifically degenerates upper and lower motor neurons in the nerve skeletal muscles (Fig. 1c). General clinical symptoms begin with motor dysfunction, such as muscle weakness, dysarthria, and dysphagia, and gradually disturb cognitive and behavioural activities, usually leading to immobility due to respiratory failure. Mutations in more than 30 susceptibility genes are associated with familial and sporadic ALS pathogenesis. These corresponding genes were commonly clustered under functions, such as protein homoeostasis, RNA homoeostasis, axonal outgrowth, cellular transport, mitochondrial alteration, oxidative stress, and neuroinflammation^287,288^. Over 70% of familial ALS cases are due to mutations in the four main genes (*SOD1, C9ORF72, TARDB* and *FUS*) associated with RNA metabolism^289^. Despite the existence of known genetic risk factors, the initial cause and interconnected pathogenetic mechanism of sporadic cases, which account for 90–95% of all cases, have not yet been clearly identified^290^.

#### Vulnerable motor neuron degeneration

Neuronal degeneration of ALS often occurs in the corticospinal fibres, partial upper motor cortex, ventral horn of the spinal cord, and precentral gyrus. However, in most cases no significant whole-brain changes were observed^291^. As the disease progresses, pathological damage in cortical regions mirrors cognitive dysfunction examined in 50–65% of patients^292^. Histologically, with motor neuron degeneration, astrogliosis appears in the anterior horn of the spinal cord and motor nuclei of the brainstem, and spongiosis and microvacuolation are predominantly observed in the motor cortex. At the lower motor neuron level, the tripartite synapse (neuromuscular junction) consists of muscle fibres and synapse-covering glial cells (Schwann cells), and the axonal terminal of the presynaptic motor neuron is prone to damage, which often progresses to the upper neurons^293^. The higher vulnerability of these neurons is explained by their fast fatigue and high metabolism^185^. In particular, long-extruding axons are more sensitive to metabolic impairment. Recent evidence shows that the significance of non-motor (systematic) symptoms progressively increases with the severity of ALS, which may be related to the spreading pathology, similar to other NDs^294^.

#### Dysfunctional protein-related pathogenesis

Similar to other representative NDs, the main hallmark of both familial and sporadic ALS is mislocated protein accumulations, including commonly hyperphosphorylated TPD43, and dipeptide repeat (DPR) protein depositions following protein superoxide dismutase (SOD1) deposits found in cytoplasm^295^. These proteins are found predominantly in motor neurons. These deposition could be explained by the disturbance of the protein degradation mechanism. However, efforts are needed to reveal the consequences of protein aggregation on motor neuron function.

More than 200 mutations in *SOD1* are known to cause neurotoxicity in several ways, including protein misfolding, proteasome impairment, excitotoxicity, oxidative stress, endoplasmic reticulum stress, impaired axonal transport, axonopathy, inflammation, altered RNA processing, and mitochondrial dysfunction^296^. Mutated SOD1 proteins are associated with motor neuron toxicity through the formation of SOD1 aggregations^297,298^. Furthermore, a recent molecular study on the effects of different mutations in *SOD1* indicated that altered conformational variants of SOD1 are more toxic than the full form of SOD1 when associated with clinical phenotypes^299^. SOD1 aggregates cause selective motor neuronal death^300^. SOD1 aggregates are found not only in the cytoplasm of motor neurons, but also in astrocytes, microglia, and oligodendrocytes^301^. Moreover, similar to other NDs, evidence gathered from studies suggests exosome transfer along anatomical pathways^302–304^ and the potential role of oligodendrocytes in the uptake and prion-like propagation of SOD1 aggregates^305^. Regardless of this evidence, other components of SOD1-linked ALS need to be studied further because of reported cases of sporadic and familial ALS without SOD1 inclusion in some brain areas^306^.

Approximately 95% of patients with familial ALS have motor neurons with mutant TDP-43 inclusions in the cytoplasm^307,308^. Under normal physiological conditions, TDP-43 is localised in the nucleus and regulates gene expression and RNA metabolism. However, hyperphosphorylation, ubiquitination, and generation of C-terminal fragments cause aggregation in the cytoplasm of glia and neurons, leading to mitochondrial dysfunction, ROS production, and nucleoplasmic transport impairment. The double spiral-fold structure of TDP-43 suggests several underlying mechanisms associated with macromolecules, prion-like aggregation, and propagation that need further investigations^309^. Correlating with an increase in cytoplasmic aggregation, lower levels of nucleus TDP-43 have led to abnormal splicing, especially in UNC13A transcripts, which are crucial for neurotransmitter-meditated communication^310–312^. However, the pathological functions of UNC13A remain unclear. Notably, TDP-43, which is found in the cytoplasm of myotubes, exerts a protective effect by integrating into muscle formation and regeneration^313^.

Lastly, hexanucleotide repeat expansion in *C9ORF72* is found in 3–7% of sporadic ALS cases (40% of familial ALS) and is a common genetic cause of ALS^314^. RNA transcripts of these extended regions form five different DPRs and RNA foci deposited in the neurons of the ALS brain exhibit toxicity in several models^315–317^. DPRs influence transport from the nucleus to the cytoplasm by causing defects in the nuclear membrane and inducing TDP-43 dislocation^318^. Following this investigation, therapeutic options have been developed. One group recently suggested an antisense oligonucleotide (ASO) that can suppress the expression of repeat groups in transgenic mice, which is considered a primary step towards gene therapy^319^. Notably, these distinct aggregations may be positive for other phenotypes. DPR aggregates are found along with TDP-43 aggregates in the ALS brain; therefore, it is important to further demonstrate their relationship and differences^320^.

#### Neuroinflammation

Imaging and postmortem cell culture studies have shown the critical roles of microglia, astrocytes, other nervous and immune cells, and inflammatory cytokines in ALS pathophysiology^321,322^. When motor neurons have degenerated, microglia are induced and secrete ROS and proinflammatory cytokines, such as TNF-α, IL-1, and IL-6. However, as the disease progresses, microglial activation becomes detrimental to motor neurons^323^. Whereas mutated *SOD1*-expressed astrocytes induced selective toxicity in spinal motor neurons^324^. Studies have suggested several pathways of astrocyte-induced neurotoxicity such as glutamate toxicity^325^, lactate impairment^326^, secretion of inflammatory mediators^327,328^, and necroptosis^329^. However, the contribution of mutant proteins to astrocyte dysfunction and motor neuron-specific toxicity remains unknown. Likewise, C9ORF72 and SOD1-expressed oligodendrocytes have also exhibited motor neuron neurotoxicity in soluble and direct ways^330^.

#### Mitochondrial dysfunction

Motor neurons are highly energetic; therefore, they tend to be sensitive to age-related metabolic changes and mitochondrial dysfunction. In ALS pathophysiology, motor neurons derived from SOD1, TDP-43, C9ORF72, and FUS show impaired mitochondrial structure, dynamics, and functions that lead to death due to the activation of ROS and intrinsic apoptotic signalling pathways^331–333^. A study of sporadic ALS patient-derived induced pluripotent stem cells (iPSCs) reported several changes in mitochondrial parameters, including increased ROS, depolarisation of the inner membrane potential, reduced ATP production, impairment of oxidative phosphorylation, and the protein import system^334^.

#### Disturbance in RNA metabolism

Many genes linked to ALS contribute to regulation of RNA metabolism, such as *SOD1, TDP-43, FUS*, and *C9ORF72*^335^. As mentioned above, the abnormal expansion of *C9ORF72* is highly related to the production of abnormal RNA nuclei loci, which contribute to neurotoxicity. The RNA-binding protein encoded by TDP-43 has a regulatory role in RNA splicing, stability, and transport, especially in the expression of ND-associated (FUS, tau, ATXN2 and progranulin), synaptic, and neurotransmitter-regulating proteins, which suggest an important role in ALS pathogenesis^336,337^.

### Huntington’s disease

HD is a rare disease with an autosomal dominant inherited pattern caused by a mutation in the Huntingtin gene (*HTT*)^338^. The progressive loss of striatal and cortical neurons leads to chorea and deterioration in cognitive and behavioural abilities, mostly as a result of the toxic effects of inherited mutant *HTT* (mHTT)-encoded large HTT proteins. At an early stage, caudate nucleus atrophy occurs and the neurons of the basal ganglia (corpus striatum) and corticostriatal circuit begin to degenerate when patients experience clinical symptoms^339^ (Fig. 1d). As the disease progresses, the cortical, occipital, and parietal regions become atrophied, corresponding to the clinical characteristics of motor and cognitive alterations^339,340^. Although the exact triggers of striatal neuron degeneration remain elusive, the majority of studies have focused on earlier pathogenesis features, such as the toxicity of mHTT and impaired DNA regulation of the *HTT* gene, which inspired several drug candidates, but all have failed at different stages. In addition, other HTT-dependent and HTT-independent pathways have been investigated, including tau dysregulation, mitochondrial dysfunction, excitotoxicity, impaired BDNF, and neuroinflammation. These pathways may have a decisive impact on HD pathogenesis, similar to mHTT.

#### CAG repeat and mutant Huntingtin protein

HTT, the main cause of HD, *HTT* gene resides on chromosome 4 and encodes the large Huntingtin protein HTT (348 kDa) with a segment of polyglutamine^341^. It is normally found in the cytoplasm or nucleus of neurons; however, its specific function has not yet been determined. Some reports have claimed that it may be related to BDNF production, axonal transport, neuroblast migration, and the overall development and communication of the nervous system^342,343^. However, in its mutated form, the *HTT* gene is composed of an aberrant number of cytosine, adenosine, and guanine (CAG) repeats ranging from 40 to 250 repeats, which ranges from 6 to 35 in healthy individuals^341^. Over 1000 CAG repeats have been reported previously^344^. The instability of CAG repeats was first discovered in the striatal neurons of mice two decades ago and later validated in a human autopsy model^345^. The number of CAG repeats correlates well with the severity of clinical HD symptoms^346^. Therefore, longer and more unstable repeats generally result in an earlier onset^13^. Early-onset HD (Westphal variant) is associated with dystonia, Parkinsonism, and psychiatric symptoms, whereas late-onset HD often correlates with 40–55 CAG repeats, which initially occur with chorea. However, the toxicity of full-length HTT proteins, especially in striatal neurons, is not fully understood, and whether they are the earliest causative factors is still debated.

Current HD pathogenesis has been broadly described as the polyglutamine tract of mHTT generated from CAG expansion and the abnormal post-translational process of HTT, which eventually leads to a cascade of pathological process^343,347,348^. The deleterious effect of mHTT was first demonstrated in mouse brains, exhibiting intracellular inclusion, striatal shrinkage, and compensatory ventricular enlargement accompanied by progressive motor alteration^349^. In addition, earlier in vivo studies provided evidence that a decrease in mHTT expression correlates with the recovery of behavioural and pathological alterations^350^.

However, it remains controversial whether full-length Huntingtin proteins or fragments are toxic to neurons^351^. When the number of the CAG repeats increases, translated large Huntingtin proteins tend to divide into fragments that aggregate, causing the death of striatal neurons^352,353^. Evidence has been gathered regarding the pathogenicity of different mHTT protein fragments. The most toxic fragment is the short HTT exon 1 protein generated by either aberrant splicing^354^ or proteolysis^355^. This protein aggregates and causes neurotoxicity in the striatal region, resulting in motor and transcriptional dysregulation in mouse models^354^. This is similar to the toxicity of full-length HTT, but tends to cause more severe neuronal death^356^.

In contrast, oligomers are more toxic than large inclusions found in cells^353,357–359^. Current evidence suggests that the N17, polyproline, and polyserine (polyS)-rich domains are crucial for aggregation and neuronal toxicity^360–362^. In contrast, some reports indicated that monomer mHTT induces apoptotic death, whereas mHTT aggregates exhibit a protective role by withholding neuron necrosis^357,363^. Additionally, a recent report claimed that HTT aggregates were not linked to degeneration of the cortical and striatal regions^364^.

Several in vitro studies have demonstrated the ability of mHTT to spread from cell to cell via tunnelling nanotubes^365^, endocytosis^366^, and prion-like behaviours to form wild-type HTT aggregate^367^. Importantly, the malfunction of the proteasome system on the clearance of mHTT has been observed in several in vitro and animal models^368,369^. Moreover, aggregated polyS and polyleucine-containing proteins exhibit propagation behaviour, which was recently observed in vivo and in vitro studies^362^.

CAG repeats are not only associated with neurotoxicity at the protein level, but mRNA-containing CAG repeats in the nucleus may also be neurotoxic. Mutant *HTT* mRNA generates clusters in the nucleus of cortical and striatal neurons in HD mice and in postmortem models^370^. However, it remains unclear whether the formation of mRNA clusters exerts neurotoxic or preventative effects^370^.

#### Tau dysregulation

Recent studies have reported a significant association between tau protein and HD pathogenesis^371^. Studies have identified tauopathy features, including misfolding, hyperphosphorylated tau aggregates, NFTs, and mHTT oligomers, in the postmortem HD brain. These features correlate with severe cognitive alterations but not with motor dysfunction^372^. Furthermore, several cellular-level studies have confirmed tau dysregulation in HD models, including altered splicing^373^ and hyperphosphorylation^374,375^ of tau proteins induced by mHTT aggregates. Further studies are needed to investigate the advanced protein crosstalk mechanisms between tau and mHTT proteins to understand the pathogenesis of HD.

#### DNA instability and oxidative stress

Recently, the focus has shifted from polyglutamine tract proteins, the product of *HTT* gene, to CAG repeats and, most importantly, dysfunction of the DNA repair system, which seems to correlate with much earlier and more severe pathogenesis^376^. This was shown in a GWAS, where uninterrupted expansion of CAG repeats in DNA, but not polyglutamine, was related to earlier and more severe pathophysiology in patients with HD^377^. Dysfunction of DNA repair proteins that induce CAG expansion was observed in the highly clustered DNA repair genes *MSH3, MLH1, PMS1, PMS2, MLH3*, and *FAN*. Therefore, these genes may be effective targets for the treatment^376,377^. Small single-stranded RNA, ASOs, restrict MSH3 expression, which lowers the rate of CAG expansion^378^. In addition, a recent study showed the influence on CAG expansion by inactivating FAN1 with a mutation in an HD model using human iPSCs(hiPSCs)^379^. This finding shows a promising approach for delaying onset and severity with genetic treatment, since FAN1 is predominantly clustered in the earlier onset of HD.

Notably, the normal HTT protein itself plays a role in transcriptional regulation by binding to support the DNA repair enzymes activated by oxidative stress^380^. mHTT directly affects the transcriptional processes of cells, mostly in the caudate nucleus of the brain and in some peripheral tissues^381^. Moreover, there is evidence on the relationship between HTT and several other transcriptional regulators, such as p53, the CREB-binding protein, CBP, and miRNAs. A recent study investigated the miRNA profiles of patients with HD and found that overexpression of hsa-mir-10b-5p inhibited *GTPBP10* expression, which may correlate with the gradual loss of mitochondrial dysfunction^382^. Furthermore, epigenetic and chromatin-modifying factors are suggested to be involved in transcriptional dysregulation. Alterations in transcriptional regulation were observed in an early HD mouse model with downregulated epigenetic regulatory genes^383^.

#### Vulnerable medium spiny neurons and excitotoxicity

In patients with HD, neurons and other cells exhibit CAG expansion^384^. Notably, typically inhibitor GABAergic medium spiny neurons (MSN) of the corticostriatal tract, specifically those in the caudate putamen and globus pallidus, are preferentially degenerated^385^. The causes of this vulnerability and degeneration still need to be better understood. However, oxidative stress and the toxic effects of mHTT aggregates on synapse formation^386,387^, and excitotoxicity^388^, similar to other NDs, may play a role. Morphologically, MSN have many spines on their axons that substantially increase in number and size at an early stage. As the disease progresses, MSN as well as other types of neurons begin to be affected^13^. Patient-derived MSN neurons displayed age-associated characteristics, such as DNA damage and mitochondrial dysfunction, as well as severe death in the presence of endogenous mHTT aggregates^387^.

Overproduction of glutamate neurotransmitters generates excitotoxicity, followed by a cascade of alterations in neurons of the corticostriatal pathway^388,389^. In a proteomic analysis of late-stage HD mouse brain tissue, convincing impairment of proteins corresponding to glutamate signalling, neurotransmitter balance, synaptic transmission, glycolysis, and ATP production was revealed^390^. Notably, astrocytes display alterations in the glutamate-GABA-glutamine regulatory system, leading to decreased GABA production and increased glutamine release. In addition to MSN, cortical neurons exhibit decreased synaptic activity, increased excitability, and reduced complex dendritic arborisation during disease progression. However, this can be reversed in the second postnatal week and HD symptoms reappear. Treatment with ampakine CX516 stopped reappearing by stabilising glutamate transmission^391^.

#### Mitochondrial dysfunction

Various mitochondrial changes occur in neuronal and non-neuronal cells, including changes in mitochondrial structure, ATP production, motility, and biogenesis, which tend to be interdependent on the mHTT protein^392–395^. The interaction of mHTT with the inner and outer mitochondrial membranes causes abnormalities in Ca^+2^ homoeostasis and mitochondrial protein dynamics^396^. Changes in the structure and localisation of mitochondria have been observed in HD models, such as decreased number and fusion, increased fragmentation and fission, and accumulation in the soma with disrupted motility^397,398^.

#### Impaired brain-derived neurotrophic factor synthesis and transport

As with other NDs, BDNF impairment is another pathophysiological feature of HD, which correlates with its anatomically vulnerable site. Cortical neurons of the corticostriatal pathway provide sufficient BDNF to the MSN, which has physiologically low BDNF mRNA^399^. Additionally, altered BDNF formation and transmission in cortical neurons with mHTT aggregates may lead to a reduction in BDNF in MSN^400^. In addition, evidence suggests that mHTT affects the BDNF transcription factor of neurons^401^ and astrocytes^402^.

#### Neuroinflammation

Whether neuroinflammation is a causative factor or a response to other pathophysiological features in HD remains unclear. Inevitably, owing to their role in extracellular glutamate clearance, glial cells are critical for studying their influence on the highly vulnerable glutamatergic MSN. There is evidence on astrocyte and microglial activation, increased proinflammatory cytokines, and hypothesised systemic inflammation, all of which are associated with disease progression^403^. Many animal and human models have indicated that astrogliosis and striatal astrocyte dysfunction are considerable components of HD pathogenesis^404,405^. In an in vivo study, glial cells expressing mHTT induced a more severe disease phenotype and caused hyperexcitability in striatal neurons^406^. In the HD postmortem model, C3 expressed-A1 astrocytes contributed to the death of neurons and oligodendrocytes while losing some functions, such as synapse formation and function, and phagocytic capacity, which confirmed the neurotoxic function of astrocytes^407^. Importantly, A1 astrocytes were induced by IL-1A, TNF, and C1q cytokines of reactive microglia and were present in other NDs, such as AD, ALS, PD, and multiple sclerosis, which makes them critical candidates for further research. In other studies, mHTT in astrocytes caused astrogliosis and impaired BDNF release^402^ and glutamate uptake^408^, leading to striatal neuronal death when co-cultured with astrocytes^409^. In a recent study, it was discovered that mHTT aggregates promote proinflammatory cytokines and ROS by activating microglia^410^.

## Neuropathogenesis-on-chips for neurodegenerative diseases

Recently, several alternative culture systems have emerged as substitutes for traditional in vitro models to provide more reliable and representative human systems. These techniques include 3D cell culture, tissue engineering with 3D bioprinting, and microfluidics^411,412^. Among these, microfluidics stands out for its capability to replicate critical elements of organs with precise control over biochemical and biomechanical aspects, offering a high-throughput, physiologically relevant, and cost-effective solution compared with conventional methods. The representative lung-on-chip replicates the alveolus, which is the key functional unit of lungs^413^. This two-chamber chip design allows externally controllable mechanical forces to mimic the alveolus movement, and direct monitoring of the interaction between epithelial and endothelial cells separated by semi-permeable membrane^413^. Moreover, this design has been applied to pulmonary disease^414^ as well as other organ units with similar mechanical conditions, such as gut-on-a-chip^415^ and glomerular-on-a-chip^416^.

The possibilities of chip-based in vitro modelling are expanding in collaboration with advanced biological and engineering techniques, such as iPSCs, 3D culture, genome editing, hydrogels, 3D bioprinting, integrated biosensors, monitoring and analysis methods, various designs, and fabrication techniques. In this section, we highlight the benefits of using 2D and 3D microfluidic chips to model NDs. First, we provide practical biological and technological guidance for modelling NDs using various designs and introduce existing examples of NDs-on-chips. Furthermore, we present a more reliable and physiologically relevant approach: a 3D culture-on-a-chip (organoid-on-a-chip) design that can be adapted for studying NDs.

### Strategy for modelling neurodegenerative diseases on a microfluidic chip

#### Choosing cell source

Approximately 86 billion neurons are interconnected in a highly ordered manner to form neuronal networks within the human brain^417^. These networks exhibit distinct activities with support from non-neuronal cells (~9.8 billion) such as astrocytes, microglia, and oligodendrocytes^417^. Cerebrovascular cells comprise 0.3% of all brain cells and play a crucial role in disease progression. Recent single-cell sequencing data revealed the existence of different subtypes within each cell type, based on their spatial location and pathological conditions at the molecular level, suggesting the complexity of brain cells^418^. Owing to the limited access to the human brain, there is a demand for the development of methods for source cells with region-specific characteristics. This is particularly important for modelling NDs because they exhibit pathogenesis specific to certain regions.

Owing to the existing animal-based in vivo and in vitro models of NDs, current knowledge was accumulated. These models offer valuable insights into cellular characteristics, disease progression, systemic and age-related changes, and the impact of the environment on the complex pathogenesis of NDs, as described in the preceding section^419–422^. In particular, mice and rats are widely used because of their closely identical genomes (~85%)^423^ and the presence of age-dependent behavioural phenotypes that can be compared with humans^424^. Transgenic mice that overexpressed or knock-in of human genes associated with familial NDs have been mostly developed. AD mouse models are created using transgenic, knock-in, and injection methods, focusing on Aβ pathology (PDAPP, Tg2576, and APP23), tau, and neuroinflammation (JNPL3, rTg4510, PS19, and 3xTg)^419^. Currently, over 170 mouse models with AD mutations mostly exhibit characteristics of EOAD. Recently, a new sporadic AD mouse model has been generated^425^. This model includes humanised Aβ by knock-in method without expressing the *FAD* mutation and exhibits age-dependent impairment of cognition, synaptic, immune response, formation of periodic-acid-schiff granules, as well as transcriptomic changes in energy, metabolism, and neuroplasticity-related genes.

However, the external approach is widely used for NDs, using various chemicals and recombinant pathological proteins. In patients with PD, drugs (reserpine and haloperidol), neurotoxins (6-OHDA, MPTP, and lipopolysaccharide), and agrochemicals (rotetone, paraquat, and manab) induce PD-like characteristics, such as motor deficits, mitochondrial dysfunction, neuroinflammation, and nigrostriatal dopaminergic cell death^420^. Numerous studies have used exogenous recombinant human and mouse proteins to characterise misfolded protein pathologies both in vitro^362^ and in vivo^426^. α-syn preformed fibrils generated from monomeric recombinant α-syn can induce hyperphosphorylation of endogenous α-syn and enhance the spread in vitro^427^ and in vivo^428^.

Non-mammalian species, such as *Caenorhabditis elegans*, *Drosophila*, and yeasts are simpler and more cost-effective alternative models^429^. Cytotoxicity, aggregation, and propagation of the α-syn protein has been investigated in these models with overexpressed *SNCA* regarding PD. Despite the genetic variations in humans, these short-lifespan species are ideal for investigating the effects of ageing on ND pathogenesis^429^.

Human embryonic stem cells (hESCs) have also been used to model NDs and to provide genetic and molecular information relevant to humans. Human neural progenitor cells (hNPCs) derived from hESCs can differentiate into several subtypes, such as cholinergic, dopaminergic, serotonergic, noradrenergic, and medium spiny striatal neurons, expressing neuron-specific markers^430^, among which H9 and H1 are dominantly used in research^431^. However, ethical concerns surrounding hESCs led to the need for alternative solutions. Methods to induce neural differentiation, such as neural induction and direct reprogramming, have been successfully developed since the discovery of iPSCs from somatic cells with transfection factors^432,433^. Current differentiation protocols utilising genetic or chemical methods have demonstrated promising results in the generation of diverse types of neural cells, including cortical, basal forebrain, dopaminergic, cholinergic, glutamatergic, GABAergic, MSN, and functional motor neurons. Additionally, these protocols can generate other types of brain cells, such as oligodendrocytes, astrocytes, pericytes, vascular endothelial cells, and microglia^434,435^. HiPSCs have been extensively used in PD studies owing to the availability of current protocols that mainly focus on the induction of dopaminergic neurons. However, for further applications, there is a need to improve and standardise the derivation methods^436^.

Familial and sporadic patient-derived iPSCs are another candidate cell source. This approach allows the generation of human-relevant pathological phenotypes that cannot be achieved using other animal models. iPSC lines from patients with PD with *SNCA, LRRK2 Parkin, CHCCHD2, PARK2*, and *PINK1* mutations exhibit important phenotypes including mitochondrial dysfunction, oxidative stress, and α-syn accumulation in induced midbrain-like dopaminergic neurons^437,438^.

High reliance on genetically modified models often overlooks sporadic cases and fails to fully consider other important factors, such as ageing and patient-specific traits. Additionally, there are inevitable limitations due to the differences between human and animal brains at the cellular and tissue levels^20,21^. While genetically engineered animal-derived cells continue to play a fundamental role in in vitro studies of NDs^439^, human patient-derived cells show promise as reliable in vitro models for basic scientific research and the development of personalised medicine.

#### Choosing microfluidic chip design

First chip application on brain research began with a notable compartmentalised in vitro system referred to as the ‘Campenot chamber’^440^. This device has two fluidically separated chambers and is used to study the effects of nerve growth factors on axonal growth. Subsequently, researchers have converted the complex characteristics of the brain into simple and miniaturised systems by mimicking diverse levels of units of the brain, including axons, neuron-glia cells, the BBB, and the neurovascular-unit^22^.

Typical microfluidic chips consist of two or more compartments (chambers) for cell co-culture. These compartments were connected by microchannels, porous membranes, and phase guides. This allows for direct or indirect interactions between homogeneous (neural circuits) or heterogeneous (neuron-glia) cell populations which are loaded into fluidically isolated chambers.

The earliest design of microfluidic chips for the CNS involved the separation of a neuronal soma from its neurites using microchannels. This design allowed for directional control of neurite growth^36^. By incorporating multiple compartments for different neuronal subpopulations, the connectivity and size of the circuits can be regulated, enabling the modelling of neural circuits in a highly ordered manner. Current neuronal chips have been designed with compartments positioned in various geometries^441^ and different diameters and numbers^442,443^, microchannels with patterned shape^444,445^, and controlled fluidic flow^446^. These features effectively manipulate neurite growth and can create both indirect and direct as well as asymmetric and symmetric neuronal connections. Moreover, valves can be established on the chip to act as a controllable barrier between compartments using external factors, such as air, oil, gel, liquid, temperature, or pressure^447–449^. Regulated fluid flow can be achieved using extra pump systems and passive hydrostatic pressure, which applies controllable shear stress to the cells. This allows for a gradient of chemicals with varying concentrations throughout the cell compartments, which is useful for disease modelling. In ND research, gradients-on-a-chip used for investigation of pathological protein dynamics and toxicity, synaptic and neurochemical interactions, vesicle transportation, and neurite ingrowth^450,451^.

Another approach involves the use of a porous membrane-based chip that acts as an interface between two or more compartments. This chip enables indirect interactions mediated by soluble chemicals and direct physical contact. The size, position, and number of the pores were customised according to the research objectives. The thickness of the membrane was adjusted to facilitate the experimental interactions. Based on this design, BBB mimicry has been achieved on chips^452,453^. It mimics the walls of blood vessels by culturing a monolayer of endothelial cells on a porous membrane, facilitating soluble interactions with brain chambers^452^. In another example, a porous membrane was placed horizontally between two chambers to culture vascular and brain cells separately^453^. This design enables more dimensional interactions than a planar culture.

#### Monitoring and analysing steps

Microfluidic chips offer several advantages over conventional models for monitoring and analysis. These include allowing optical clarity for real-time monitoring under various chemical or physical cues, gas permeability, and continuous flow to balance nutrients or waste for longer viability; fluidic isolation for multi-omics analysis of soluble biomarkers from distinct cell populations; and microengineering to integrate biochemical, electrochemical, and electrophysiological biosensors.

Most microfluidic chips are composed of clear optical materials (polystyrene and polydimethylsiloxane). Additionally, most designs include a pre-determined image acquisition region within their structures, which enables consistent and accurate monitoring. Specific biomarkers can be detected directly from the chip using fluorescence and confocal microscopy by introducing a very small amount of staining dyes and antibodies through the fluidic channel inlet. However, issues of contamination, accumulated cell debris, and air bubbles in tiny channels can pose challenges in maintaining cultures and imaging. These can be prevented by carefully processing the fabrication steps, using trapped air bubble methods, or force-out procedures^454^.

The integration of various types of biosensors on microfluidic chips enables the continual measurement of the biochemical and electrophysical properties of cell cultures. Various parameters, such as oxygen, pH, and soluble metabolite levels can be analysed using immune- and enzyme-based sensors, allowing the evaluation of cellular survival and metabolism in response to induced pathophysiological conditions^454^. Moreover, the incorporation of automated electrochemical biosensors in microfluidic chips enables the continuous monitoring and analysis of dynamic cellular activity^455^.

In the case of the brain-on-a-chip, a transendothelial electrical resistance system, integrating electrodes^38,456^, and planar electrophysical multielectrode arrays (MEA)^457^ are often incorporated to measure the integrity and permeability of the BBB and assess neuronal electrical signals, respectively. Off-chip analysis tools, such as mass spectroscopy, liquid chromatography, and biochemical assays, such as ELISA, can be utilised to analyse small amounts of samples isolated from microfluidic chip cultures. Alternatively, whole-cell culture samples can be lysed and collected for further genomic, transcriptomic, and proteomic analyses. In certain cases, rather than opting for the commonly used closed approach, an open-chip design offers various benefits. The open chambers provide easier exposure to additional chemical components, imaging, transfer for analysis, and sample collection. Notably, most open designs tend to lose their dynamic fluid flow, which can limit the controllability and relevance. However, the benefits of using open chips have also been reported in recent studies. Salman et al. ^458^ developed an open chip for modelling the human brain endothelial microvessel. It enables the unidirectional exchange of media, gas, and nutrients within the collagen gel through a cylindrical hollow lumen. The top opening increases oxygen availability. Furthermore, it simplifies the harvesting of cells and exosomes for subsequent transcriptomic and proteomic analyses and allows for standard and advanced imaging by carefully optimising the dimensions and geometry.

#### Application in modelling neurodegenerative disease pathogenesis

Based on this research objective, relevant anatomical and physiological units, including the neuromuscular junction (NMJ), corticostriatal pathway, substantia nigra, BBB, glymphatic system, neurovascular unit (NVU), and gut-brain axis, can be replicated using matched chip designs. However, most existing ND studies using microfluidic chips still lack physiological relevance owing to the choice of cell source and designs under 2D and static conditions. These drawbacks limit the wide applicability of microfluidic chips. The limitations of 2D systems include their inability to generate complex interactions, waste removal, and oxygen diffusion. A planar environment reduces morphology, proliferation, differentiation ability, cellular signalling, and gene expression which affects the in vivo relevance of hiPSCs^459^.

Conversely, the implementation of a 3D extracellular matrix (ECM) gel provides mechanostructural and biochemical cues to cells which have improved relevance in cellular morphology, migration behaviour, cellular signalling, interactions, and substantial gene expressions^460^. Moreover, it allows time-dependent differentiation and neurodegenerative changes^461^. The important factor of NDs, ageing, alters properties of the brain ECM^460^. Aβ induced accumulation and aggregation of pTau has been observed in 3D in vitro condition^462,463^. This evidence indicates the importance of ECM in modelling NDs.

To characterise a 3D microenvironment that provides not only mechanical, but also biochemical cues, the properties of the ECM, such as pore size, solute retention, attachment motifs, biodegradability, and stiffness, must be considered^460^. Alterations in these properties can affect the behaviours of various brain cells as well as disease pathogenesis^464,465^. During AD, owing to altered functional connectivity, vulnerable regions of the brain exhibit altered stiffness, which is correlated with structural changes^466^. Furthermore, evidence shows that ECM stiffness directly affect the aggregation and propagation of pathogenic molecules, such as Aβ and tau oligomers^460^.

Various hydrogels have been integrated into microfluidic chips to demonstrate direct interactions while creating a more physiologically relevant brain tissue-like environment^467,468^. One example of this design utilises a phase guide in the central channel to confine ECM gel-embedded cells from dispersing into side flow channels filled with medium, while still allowing interaction without a barrier^461,468^. In the next section, we provide an overview of the use of custom and commercial microfluidic chips to model NDs pathogenesis.

### Existing examples of neurodegenerative disease-on-chips

#### Alzheimer’s disease-on-chips

Existing studies using microfluidic chips have investigated substantial AD pathogenesis, including Aβ and tauopathy, mitochondrial dysfunction, and neuroinflammation, providing valuable new insights that were not observed using conventional culture methods (Table 1). In particular, numerous neurons-on-a-chip are used for tau propagation^469–473^, and Aβ toxicity studies^474–476^. Neurons-on-a-chip allows the separation of the soma and neurites, facilitating the real-time visualisation of proteinopathy. Some groups have found evidence of tau transfer via exosomes^110^ and synapses^477,478^ using 2–3 compartment chips, suggesting prion-like propagation of tau.

**Table 1:** Summary of Alzheimer’s disease-on-chips

*Alzheimer’s disease-on-chips*						
Neuropathogenesis			Chip model			*Ref*.Fig.
Targeted pathogenesis	Cell source	Modelling approach	Features	Type	Design	
Neuroinflammation	hNeu/Ast/MG	*FAD(670N/M671L/V717I)* Endo Aβ/pTau	3D/S/C	Circular migration chip	2CHRs/MCs	^482^ Fig. 2b
BBB dysfunction	hNeu/Ast/Peri/EC	*APP/PSEN1* Endo Aβ	3D/S/C	BBB-on-a-chip	5MCs/Bs	^452^ Fig. 2c
Dysfunction of AQP4	hAst/HBMEC	Exo Aβ/LPS	3D/D/C	Glymphatics-on-a-chip	Central CHRs/2MCs	^486^
Immune response to Aβ	hNEUT	Exo Aβ mono	2D/S/C/G	Chemotaxis chip	3CHRs/3Vs/MCs	^483^
Immune response to Aβ	hMG	Exo Aβ mono, oli	2D/S/O/G	Circular chemotaxis chip	2CHRs/MCs	^484^
Aβ toxicity	pCoNeu	Exo Aβ	3D/D/C	Neurospheroid chip	50 wells/ OP	^485^ Fig. 2d
Aβ toxicity	pCoNeu	Exo Aβ	2D/D/C/G	Spatial G chip	MCs/OP	^451^ Fig. 2a
Aβ toxicity	pHiNeu/MG	Exo Aβ oli, fib	2D/D/O	Circular co-culture chip	4CHRs/MCs	^479^
Aβ toxicity	pCoNeu	Exo Aβ/Glutamate	2D/S/C	Axon-on-a-chip	2CHRs/MCs	^474^
Aβ toxicity	pCoNeu	Exo Aβ oli	2D/S/O	Axon-on-a-chip	2CHRs/MCs	^475^
Aβ toxicity transfer	pCoNeu	Exo Aβ mono	2D/S/C	Axon-on-a-chip	2CHRs/MCs	^574^
Aβ effect on tauopathy	pCoNeu/pHiNeu	Exo Aβ/pTau	2D/S/C	Axon-on-a-chip	2CHRs/MCs	^476^
Tau transfer	pHiNeu	hTau(*P301L*)	2D/S/C	Neuronal circuit chip	3CHRs/MCs	^478^
Tau transfer by exosome	pHiNeu	H GFP-tau exosomes	2D/S/C	Axon-on-a-chip	2CHRs/MCs	^110^
Tau transfer, aggregation	pCoNeu	hTau	2D/S/C	Axon-on-a chip	3CHRs/MCs	^575^
Tau transfer	pCoNeu/PHiNeu	hTau(P301L)	2D/S/C	Axon-on-a-chip	3CHRs/MCs	^477^
Tau transfer	pCo/HiNeu	hTau	2D/S/C	Axon-on-a-chip	2CHRs/MCs	^469^
Tau transfer	pCoNeu	hTau(*P301L)*	2D/S/C	Axon-on-a-chip	3CHRs/MCs	^470^
Tau transfer	pCoNeu	hTau	2D/S/C	Axon-on-a-chip	2CHRs/MCs	^471^
Tau transfer	pCoNeu	G of OA/pTau	2D/D/C/G	Axon-on-a- chip	2CHRs/main MC/24MCs	^473^
Tau transfer	hiPSCs Neu	Exo Cy5-Tau mono, oli	2D/S/C	Axon-on-a-chip	2CHRs/MCs	^472^

*AQP4* aquaporin, *Ast* astrocyte, *Aβ* amyloid beta, *B* barrier, *BBB* blood-brain barrier, *C* closed, *CHR* chamber, *Cy5* cyanine 5, *MC* microchannels, *D* dynamic, *EC* endothelial cell, *endo* endogenous, *exo* exogenous, *fib* fibril, *G* gradient, *GFP* green fluorescent protein, *H* human, *HBMEC* human brain microvascular endothelial cell, *hiPSC* human-induced pluripotent stem cell, *LPS* lipopolysaccharide, *MG* microglia, *NEUT* neutrophil, *O* open, *OA* okadaic acid, *mono* monomer, *oli* oligomers, *OP* osmotic pump, *pCoNeu* primary cortical neuron, *Peri* pericyte, *pHiNeu* primary hippocampal neuron, *pTau* hyperphosphorylation of tau, *S* static, *V* valve.

**Fig. 2:**
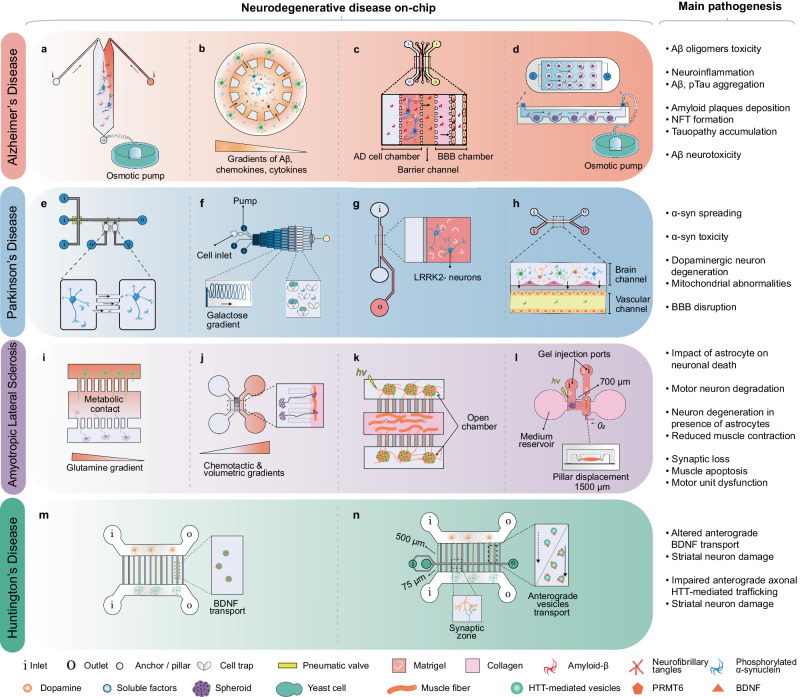
Examples of microfluidic chips for NDs modelling. AD - **a** Gradient chip with interstitial flow. The effect of Aβ oligomers on neurons was observed in gradient concentration condition^451^. **b** 3D static neuroinflammation-on-a-chip. Microglia migrated in response to inflammatory cytokines across microchannels to the centre chamber where AD neurons and astrocytes were cultured^482^. **c** BBB-on-a-chip. Formation of amyloid plaques, neurofibrillary tangles, Aβ, and increased permeability of brain ECs, were fully recapitulated in a 3D environment^452^. **d** Dynamic neurospheroid-on-a-chip. A flow of exogenous Aβ caused by an osmotic pump resulted in axonal degeneration and cell death^485^. PD- **e** Navie and GFP-tagged α-syn expressing neuroglioma cells were co-cultured to monitor the spreading of α-syn^494^. **f** Gradient chip. It modulated the intracellular α-syn expression of singularly trapped yeasts in system with galactose gradient^450^. **g** Dopaminergic neurons-on-a-chip. In a 3D condition, it recapitulated mitochondrial abnormalities and degeneration of dopaminergic neurons with the PD-related mutation^461,577^. **h** Substantia nigra and vascular barrier chip. hiPSCs-derived midbrain dopaminergic neurons, primary glia cells and a brain microvascular EC line were separated by a porous membrane to assess BBB on-a-chip dysfunction, progressive neuronal loss, neuroinflammation, and astrogliosis^453^. ALS- **i** 3-chamber-chip allowed metabolic interaction between SOD1-mutated astrocytes and cortical neurons through microchannels in a glutamate gradient condition^502^. **j** ALS chip. Applying chemotactic and volumetric gradients successfully formed interactions between FUS-mutated motor neurons and mesangioblast-derived myotubes through microchannels^503^. **k** An open compartmentalized NMJ device was used to co-culture optogenetic motor neuron and *SOD1*-mutated astrocytes as a spheroid to assess the muscle denervation pathology of ALS^505^. **l** TDP-43 mutated motor neuron spheroid and muscle fibres were co-cultured in a 3D condition between two separate reservoirs, which recapitulate ALS pathologies^506^. HD- **m** Impaired BDNF transport due to mHTT expression through cortical axons resulted in striatal neurons degeneration^509^. **n** The synaptic connection between cortical axons and striatal dendrites was formed by the different lengths of microchannels and a separate synaptic channel, assessed the early progression pathology^507^. AD Alzheimer’s disease, ALS amyotrophic lateral sclerosis, BBB blood-brain barrier, EC endothelial cell, HD Huntington’s disease, hiPSC human-induced pluripotent stem cell, NMJ neuromuscular junction, PD Parkinson’s disease.

Aβ-related pathology is also being investigated mainly using microfluidic chips consisting of two to four compartments. Substantial evidence of Aβ toxicity has been gathered, including the induction of hyperphosphorylation of tau proteins, degeneration of axons^474^, and disruption of BDNF retrograde signalling of axons^475^. Some studies have cultured neurons as well as co-cultured microglia with primary hippocampal neurons in the presence of exogenous Aβ proteins. Ruiz et al. ^479^ studied the glial clearance of different forms of Aβ, in a 4-compartment circular open microfluidic system. Each open compartment allowed for four parallel experiments and easy seeding co-cultures of different populations, such as microglia and neurons, with various cell densities under gravity-induced dynamic conditions. Aβ oligomers have a higher toxicity to hippocampal neurons compared to that of fibrils. However, only the microglia changes in Aβ exposure; the neuronal aspect is not included. Choi et al. ^451^ developed a microfluidic chip that can generate dynamic fluid conditions for gradient formation of Aβ aggregates, similar to in vivo studies, by integrating an osmotic pump to induce slow laminar flow (0.25 µL/min), similar to the interstitial flow rate (Fig. 2a). Under dynamic conditions, the cultured primary rat neurons showed a higher level of viability (7.5%). However, when exposed to Aβ oligomers, the neurons lost their synaptic activity and eventually experienced apoptosis. In addition, the fibril form of Aβ was less toxic to neurons than oligomers.

Several on-chips studies have been conducted using human neural progenitor cells transduced with familial AD-specific mutated genes, such as *FAD 670N/M671L/V717, APP*, and *PSEN1*. In AD pathogenesis, astrocytes play an essential role in the progression of AD, with “a double-edged sword” effect on neuroinflammation, owing to their neuroprotective and neurotoxic effects^138,480,481^. However, the dynamics of astrocytes and pathogenic proteins involved in neuroinflammation are not well understood. Park et al. ^482^ designed a simple circular chip that has two chambers (central and radial) which allow microglia migration towards to the centre where AD neurons derived chemokines and cytokines (TNFα, IL-1, and IL-6) are located (Fig. 2b). This design created a gradient of Aβ and neuroinflammatory molecules in a 3D environment and showed great potential for further molecular studies, including co-culture with glial cells. Additionally, numerous studies have been conducted on the migration of human neutrophils^483^ and microglia^484^ towards exogenous Aβ monomers and oligomers with chemotaxis chips in 2D conditions.

By incorporating various types of cells, a molecular understanding of the neuroinflammatory phenomenon can be obtained using microfluidic chips. Recently, research was focused on well-defined brain features, such as the BBB and the neurovascular unit, in AD neuropathogenesis. Shin et al. ^452^ developed a BBB-on-a-chip comprising a cell chamber for AD-mutated hNPCs and a tube-shaped endothelial cell chamber separated by a central barrier. As shown in Fig. 2c, the accumulation of Aβ and pTau proteins were observed in the brain channel. Upon removal of the barrier by Matrigel application, the proteins are transferred to endothelial cells, where they are deposited in the cytoplasm. They cause damage to tight junctions, indicating BBB disruption, which is observed in the early stages of AD. These promising results suggest that BBB-on-a-chip can be used as a drug-screening tool in the future.

A microfluidic chip composed of 50 cylindrical wells was developed to culture neurospheroids and integrate them with an osmotic micropump to provide a continuous medium supply similar to the interstitial flow rate^485^ (Fig. 2d). Medium with exogenous Aβ flows over neurospheroids continuously, causing axonal degeneration, synaptic dysfunction, and neuronal death. The spherical wells of this chip generate more homogeneous spheroids, and continuous flow leads to increased viability, which directly affects cell-cell interactions. This chip demonstrated that integrating microfluidic chips with 3D cultures can create more in vivo-like conditions that are unavailable in other conventional systems.

Unique characteristics of the glymphatic clearance system can be explored with microfluidic chips by allowing dynamic glymphatic flow in the space between astrocytes and endothelial cell co-culture^163^. Lee et al. ^486^ recently replicated the gliovascular unit using a microfluidic chip in AD studies. The glymphatics-on-a-chip design allows for the co-culturing of human astrocytes and blood vessels in 3D gel-embedded parallel microchannels. LPS-induced neuroinflammation causes altered fluid drainage and Aβ clearance through matrisome- and cell-dependent fluid transport pathways.

#### Parkinson’s disease on-chips

The majority of existing PD-on-a-chip studies have focused on recapitulating α-syn related pathogenesis (Table 2). However, only a few have used dopaminergic neurons to increase the relevance of these models. The first on-a-chip PD model was developed using simple single-linear microchannels with a gradient of neurotoxins to model the apoptosis of dopaminergic neurons caused by 6-OHDA. Depending on the concentration, this neurotoxin can induce apoptosis or necrosis^487^. By generating a back-and-forth continuous flow, the model achieved low and high concentration gradients of 6-OHDA, which resulted in PC12 cell line apoptosis by oxidatively damaging the mitochondria.

**Table 2:** Summary of Parkinson’s disease-on-chips

*Parkinson’s disease-on-chips*						
Neuropathogenesis			Chip model			*Ref* Fig
Targeted pathogenesis	Cell source	Modelling approach	Features	Type	Design	
Inflammation/ BBB disruption	ptPSCsNeu/Peri/Ast	LRRK2-G2019S	3D/S/C	BBB-on-a-chip	3CHRs/B	^497^
BBB disruption	hiPSCs DANeu/pAst/MG/Peri/HBMECs	Exo h α-syn mono, fib	3D/D/C	BBB-on-a-chip	2CHRs/B	^453^ Fig. 2h
Mitochondrial abnormality/ α-syn toxicity	HNESCs DANeu	LRRK2-G2019S	3D/S/C	Co-culture chip	CHRs/B	^461^ Fig. 2g
α-syn transfer, toxicity	H4 cell line	Exo α-syn-GFP	2D/D/C	Co-culture chip	2CHRs/1MC/V	^494^ Fig. 2e
Mitochondrial damage	PC12 cell line	6-OHDA	2D/D/C/G	G chip	Linear MCs	^487^
α-syn inclusion	*S. cerevisiae*	GFP-α-syn SNCA(*A53T*)	2D/S/C	Automated chip	CHRs/Ts	^496^
α-syn inclusion	*S. cerevisiae*	GFP-α-syn mono,fib	2D/S/C/G	G generator chip	CHR/Ts	^450^ Fig. 2f
α-syn transfer	hiPSCs CoNeu	Exo α-syn mono, fib	2D/S/C	Axon-on-a-chip	2CHRs/ asymmetric MCs	^495^
α-syn transfer	pHiNeu	Exo α-syn fib	2D/S/C	Axon-on-a-chip	3CHRs/MCs	^576^
α-syn transfer	pHiNeu	Exo α-syn fib	2D/S/C	Neuronal network chip	2-6IC/Heart-arrow MCs	^441^
α-syn transfer	pCoNeu	Exo α-syn fibrils	2D/S/C	Axon-on-a-chip	2CHRs/MCs	^488^
α-syn transfer	pCoNeu	Exo α-syn fib	2D/S/C	Axon-on-a-chip	2CHRs/MCs	^489^
Mitochondrial abnormalities	pCoNeu	Rotetone	2D/S/C	Axon-on-a-chip	2CHRs/MCs	^490^
Mitochondrial abnormalities	ptPSCsNeu	SNCA(*E46K,E57K*) α-syn oli	2D/S/C	Axon-on-a-chip	2CHRs/MCs	^492^

*6-OHDA* 6-hydroxydopamine, *Ast* astrocyte, *B* barrier, *BBB* blood-brain barrier, *C* closed, *CHR* chamber, *D* dynamic, *DA Neu* dopaminergic neuron, *ECM* extracellular matrix, *exo* exogenous, *fib* fibril, *G* gradient, *GFP* green fluorescent protein, *hBMEC* human brain microvascular endothelial cell, *hiPSC* human-induced pluripotent stem cell, *hNESC* human neuroepithelial stem cell, *IC* interlocking units, *MC* microchannel, *MG* microglia, *mono* monomer, *oli* oligomer, *pCoNeu* primary cortical neuron, *Peri* pericyte, *pHiNeu* primary hippocampal neuron, *ptPSCs* patient pluripotent stem cells, *S* static, *T* trap, *V* valve, *α-syn* alpha synuclein.

The microfluidic chip with two chambers connected through microchannels is the most common method for investigating α-syn inclusion and propagation. This chip design allows for clear observation of the process of α-syn fibril uptake, and its anterograde or retrograde transport through axons to other neurons by exposing neurons to exogenous α-syn^488,489^. Van Laar et al. ^490^ used the same microfluidic chip to emulate the dynamics of mitochondrial alterations observed in the earlier stages of PD. In PD, neural degeneration begins in long, less myelinated and distant axons^491^. This was confirmed by their response to rotenone; mitochondrial biogenesis increased in distal axons, followed by cell bodies and dendrites. Prots et al. ^492^ also used the same chip to investigate the toxicity of α-syn oligomers in neurons derived from iPSCs from patients with PD with mutant α-syn (E46K and E57K). They observed α-syn oligomerization led to the impairment of anterograde mitochondrial transport through axons, and structural synaptic loss. This result confirmed the role of mutant α-syn oligomers in the early pathogenesis of PD. Additionally, one group cultured preformed primary neurons with exogenous α-syn fibrils for an α-syn transfer study based on evidence that exogenous recombinant α-syn can promote misfolding of physiological α-syn, leading to the formation of LB-like aggregates in neurons^493^. In this experiment, a three-chamber chip was used to demonstrate the toxic effect of α-syn aggregation and uptake by inhibiting regulator protein of protein trafficking. They also investigated how endogenously released α-syn are toxic to separately cultured neurons in a paracrine way.

An example of a customised chip with micro-valves that allows control fluid dynamics has been developed to demonstrate α-syn uptake and spread^494^ (Fig. 2e). By co-culturing cells expressing GFP-tagged α-syn and normal cells in two chambers connected through microchannels, the researchers were able to observe α-syn successfully spreading throughout two different populations. Another group reconstructed a human corticocortical neuronal network by culturing hiPSCs on a microfluidic chip^495^. In this design, neurons in the two-cell chambers interact with each other in a narrow central chamber by projecting their axons through asymmetric microchannels. They indicated that prion-like propagation of physiological α-syn occurs between human neurons.

Two-, and three-, or multichambered chips recapitulate neuron to neuron α-syn spread. A microfluidic chip with two to six interlocked chambers that can be adjusted in several different ways successfully recapitulated the complex neuronal network without barriers and demonstrated phosphorylated α-syn propagation^441^. A well-known design of microfluidics, the gradient-generator chip, can provide a reliable chemical concentration by creating nine different doses in microchannels^450^ (Fig. 2f). They induced intracellular α-syn with exogenous α-syn monomer and fibrils inside *Saccharomyces cerevisiae* which were trapped individually in the chip with different chemical gradient environments. Recently, another group cultured live yeast on a microfluidic chip integrated with automated feedback control, which can regulate the concentration of α-syn aggregation in *S. cerevisiae* expressing A53T α-syn combined with GAL-inducible promoter with GFP in real-time^496^. Over a galactose gradient, they determined the threshold of α-syn mutant protein expression that induces the formation of inclusions and their clearance, which is strictly based on autophagy at the single-cell level. This approach can be applied to other quantitative studies of human neuronal cell lines.

As dopaminergic neurons are selectively damaged during the progression of PD, Bolognin et al. ^461^ studied the effect of the LRRK2-G2019S mutation on dopaminergic neurons (Fig. 2g). In this experiment, they used a “*Mimetas*” culture chip with a phase guide that allows culturing cells in 3D ECM conditions. The dopaminergic neurons successfully formed structurally relevant networks on the chip. Notably, substantial changes due to the PD mutation, including neurodegeneration and changes in the number and complexity of mitochondria, were only observed after six weeks in the 3D condition. This result demonstrates the importance of mechanical cues in demonstrating physiologically relevant pathogenesis.

Considering the significance of the BBB in PD pathogenesis, one group has successfully demonstrated the BBB dysfunction caused by the toxicity of exogenous human α-syn fibrils^453^ using a 3D microfluidic chip (Fig. 2h). The design of the chip allows the culturing of brain and vascular cells in tissue-specific ECM cocktails in two separate channels connected through a horizontal semi-permeable membrane. This mimicry of BBB has exhibited formation of intracellular inclusion by internalised α-syn, which leads to a progressive loss of dopaminergic neurons, mitochondrial dysfunction, and neuroinflammation associated with glia cell activation. Brain endothelial cells were cultured in support of pericytes, microglia, and astrocytes, therefore, the BBB was formed with higher maintenance and functionality. Moreover, dopaminergic neurons, the main cells of the substantia nigra, stably secrete dopamine, indicating that functional maturation is not considerable in conventional cultures.

More recently, de Rus Jacquet explored astrocytic activity in both BBB dysfunction and inflammation during PD pathogenesis^497^. The significance of the chip design is that the ECM gel (collagen) acts as a barrier between the brain vascular channels which allows direct interaction. The phenotypes of vascular changes in vitro which were observed in sporadic PD and human postmortem models were successfully demonstrated and vascular changes depended on LRRK2-G2019S-expressing astrocytes, which exhibit proinflammatory activity. In this model, LRRK2-G2019S-expressing patient-derived astrocytes failed to support BBB integrity and inflammation, particularly angiogenesis, as confirmed by RNA-seq data. Moreover, mutated astrocytes regulated BBB integrity via the MEK1/2 signalling pathway, providing evidence for the importance of astrocytes in PD pathogenesis.

#### Amyotrophic lateral sclerosis on-chips

Numerous rodent models have been developed to investigate ALS based on newly discovered genetic factors. These models have become indispensable tools for investigating disease mechanisms, although they mostly reflect familial case of ALS^498^. In vitro modelling, mostly using cells sourced from transgenic mice with familial gene mutations, such as *TARDBP, SOD1*, and *C9ORF72*, has also revealed important dysfunctions of cellular processes related to ALS which are also shared with sporadic ALS^498^. These dysfunctions appeared in protein transport, homoeostasis, RNA transcripts, and mitochondria^499^. Although such models provide defined mutation-related information, a specific model for sporadic ALS has not been developed^421^. In ALS, several connected mechanisms lead to motor neuron death, causing diverse phenotypes that are difficult to model. Mutations in RNA-binding proteins, such as TDP-43, FUS, NRPNA1, MATR3, and SETX, alter RNA metabolism^500^. Therefore, a unified model that reflects a patient’s phenotype is required.

Previous ALS studies have investigated toxic protein aggregation and propagation using simple 2-chamber microfluidic chips (Table 3). Westergard et al. ^501^ determined that C9ORF72-linked DPRs spread between cortical neurons via exosomes and non-exosomes via axonal anterograde and retrograde transmissions. This study once again proved that the spread of toxic proteins is a common underlying mechanism of progressive NDs.

**Table 3:** Summary of the amyotrophic lateral sclerosis-on-chips

*Amyotrophic lateral sclerosis-on-chips*						
Neuropathogenesis			Chip model			*Ref*. Fig.
Targeted pathogenesis	Cell source	Modelling approach	Features	Type	Design	
NMJ impairment	hiPSCs MN/hMesangioblast	FUS(*P525L, R521H*)	3D/S/C/G	NMJ-on-a-chip	2CHRs/MCs	^503^ Fig. 2j
NMJ impairment	hiPSCs MN/pMyoblast	C9ORF72	2D/S/C	Radial chip	2CHRs/MCs	^504^
NMJ impairment	ESCs MN/Ast/pMyofibres	SOD1(*G93A*)	3D/S/O	NMJ-on-a-chip	3CHRs/MCs/2 anchors	^505^ Fig. 2k
NMJ impairment	ptMN hiPSCs Myoblasts	TDP-43(*G298S*)	3D/S/C	NMJ-on-a-chip	2CHRs/pillars	^506^ Fig. 2l
NMJ impairment	pCoNeu/Ast	SOD1(*G93A*)	2D/D/C/G	NMJ-on-a- chip	2CHRs/main MC/MCs	^502^ Fig. 2i

*Ast* astrocyte, *C* close, *CHR* chamber, *D* dynamic, *ESC* embryonic stem cell, *G* gradient, *h* human, *hiPSC* human-induced pluripotent stem cell, *MC* microchannel, *MN* motor neuron, *NMJ* neuromuscular junction, *O* open, *P* primary, *pCoNeu* primary cortical neuron, *Pt* patient, *S* static.

Similar to other NDs, neuroinflammation is a pathogenic feature of ALS. Accordingly, astrocytes were simulated using microfluidic culture systems. In one of the earliest studies, Kunze et al. ^502^ co-cultured primary cortical neurons and SOD1-mutated astrocytes without direct contact to demonstrate the effect of astrocytes on neuronal death under a glutamate gradient condition (Fig. 2i). They observed neuronal death and decreased synaptic protein expression in neurons in the presence of astrocytes from patients with ALS, indicating SOD1 toxicity.

Considering that NMJ degeneration is the main hallmark of ALS, the co-culture of myocytes and motor neurons has been mostly recapitulated for ALS studies, taking advantage of the compartmentalised elements of microfluidic chips. Several groups have used different types of culture systems, including monolayer cultures and spheroids derived from genetically modified ESCs and hiPSCs^503–506^. Radial-shaped two-chamber microfluidic chips demonstrated mitochondrial toxicity, protein synthesis dysfunction, and axonal phosphorylated TDP-43 deposition associated with the activity of C9ORF72-modified hiPSCs neurons^504^. Another research group successfully established NMJ impairment in ALS by culturing hiPSC-derived FUS-mutated neurons and human primary mesangioblast^503^ (Fig. 2j). They generated NMJ by creating a volumetric gradient of BDNF, GDNF, and CNTF in microfluidic chambers to induce axonal growth in the muscle cell chambers through a microchannel. They found that FUS neurons exhibited a lower axonal growth rate, leading to fewer NMJ. However, an HDAC6 inhibitor reversed axonal growth in FUS neurons. Machado et al. ^505^ developed an open microfabricated chip composed of three compartments, which enabled the analysis of interactions between spheroids containing ESC-derived neurons and SOD1 astrocytes as well as primary myofibres (Fig. 2k). They investigated how astrocytes with mutated SOD1 induce neuronal loss, followed by myofibril dysfunction. However, these advanced models lose their physiological relevance when primary rodent myofibres are used, despite the use of human ALS-specific motor neurons. An exceptional example of such a model is co-cultured patient-derived motor neuron spheroids and hiPSC-derived skeletal myoblasts on a custom-made 3D microfluidic chip^506^ (Fig. 2l). Once skeletal muscle fibres are formed, motor spheroids are introduced into a 3D gel to form NMJ. Moreover, optogenetic technology has been applied to motor neuron spheroids by transfecting them with a light-sensitive ion channel (channelrhodopsin-2), allowing the non-invasive control of motor neuronal activity. The chip design also includes several important features, such as a separate reservoir allowing for the co-culturing of cells. The region for neurite elongation was designed to be longer to achieve a physiologically relevant neurite length. The pillars in these designs provide functions, such as limiting the size of the spheroid, preventing core necrosis, and measuring the strength of muscle contraction by changes in pillar displacement. By generating NMJ, they successfully recapitulated TDP-43 aggregation, neurotoxicity, and muscle atrophy due to synaptic loss.

#### Huntington’s disease on-chips

Compared with other NDs, there have been relatively few microfluidic chip applications for HD (Table 4). These chips replicate the corticostriatal network, a vulnerable region in HD pathophysiology, with two-^406^ and three-chambers^507,508^. To understand the toxic effects of mHTT on the corticostriatal neural circuit, on-a-chip studies commonly use transgenic mouse neurons. One group investigated how mHTT reduced the cortical axonal transport of BDNF and detected a positive effect of the TCP-1 ring complex (TRiC) on decreasing mHTT levels^509^ (Fig. 2m). Chip contains two separate chambers for cortical and striatal neurons, connected by microchannels. Design allows the observation of striatal neuron degeneration, and decreased synapses. In their three-chamber chip (presynaptic, synaptic, and postsynaptic), Virlogeux et al. ^508^ established laminin and poly-D-lysine gradients from presynaptic and synaptic chambers. By culturing mHTT-transduced primary neurons, deficiencies in synapses, BDNF, and glutamatergic trafficking, and a decline in mitochondrial transport and vesicle synthesis were observed. Later, this group used the chip in a drug target study by recapitulating the restoration of Huntingtin-dependent BDNF vesicle transport by activating palmitoylation^510^. They observed improved axonal BDNF trafficking, synapse formation, and viability in the chip channels. Based on an in vivo study of HD knock-in mice that showed improvements in motor deficiency and behavioural changes, the authors claimed that ML348, which inhibits acyl-protein thioesterase 1 (APT1), could be used as a therapy against disease progression. Using the same chip, Migazzi et al. ^507^ identified distortion in the anterograde axonal transport of HTT-meditated vesicles and neuronal damage due to arginine methylation-defective HTT(Fig. 2n). They identified how PRMT6 expression could restore HTT-mediated axonal trafficking. To model the corticostriatal pathway, they reconstituted two separate cell chambers connected through microchannels of different lengths and the middle “synaptic” chamber.

**Table 4:** Summary of Huntington’s disease-on-chips

*Huntington’s disease-on-chips*						
Neuropathogenesis			Chip model			*Ref*. Fig.
Targeted pathogenesis	Cell source	Modelling approach	Feature	Type	Design	
mHTT toxicity	pCoNeu/StrNeu	mHTT (*548-17q r118k*)	2D/S/C	Corticostriatal on-a-chip	3CHRs/MCs	^507^ Fig. 2n
Corticostriatal network dysfunction	pCoNeu/StrNeu	mHTT (*140 CAG repeat*)	2D/S/C/G	Corticostriatal on-a-chip	3CHRs/unilateral MCs	^508^
Corticostriatal atrophy	pCoNeu/StrNeu	mHTT (*97CAG full-length*)	2D/S/C	Corticostriatal on-a-chip	2CHRs/MCs	^509^ Fig. 2m

*C* closed, *CHR* chamber, *G* gradient, *MC* microchannel, *mHTT* mutant Huntingtin protein, *pCoNeu* primary cortical neuron, *S* static, *StrNeu* striatal neuron.

In another study, basal ganglia circuits were composed of the cortex, striatum, globus pallidus internal, substantia nigra reticularis, compacta, and thalamus using a chip with five chambers linked to microchannels^511^. Notably, the size and location of each regional chamber were designed to mimic a similar ratio of cells and interactions as the 3D anatomical structure of the basal ganglia circuits. They demonstrated the direct neuronal connectivity between five different cell populations using rodent primary neurons. The striatum and its communications with connected regions are important neuronal circuits in HD studies.

Moreover, by integrating an MEA with 256 electrodes, this microfluidic chip analysed the neuronal network functionality in real time. Although the experiment has not yet characterised the disease-specific pathophysiology, it is possible to detect the effect of Huntingtin or α-syn proteins in neural circuit function using this in vivo relevant MEA-integrated regional-neuron network chip. Microfluidic chips can be modified in several ways. One research group recently proposed an electrostimulation microfluidic chip by modifying the chip design for current HD disease modelling. They created holes with electrodes in cell chambers to directly stimulate neurons. This design recapitulated and successfully measured the neuronal activity of the corticostriatal circuit but has not been applied to HD studies yet^512^.

### Microfluidic-based 3D in vitro models

Brain organoids help characterise disease-specific brain regions with distinct cellular makeup, such as the forebrain, midbrain, and hindbrain. Several approaches have been developed to model NDs using brain organoids. To induce Aβ protein accumulation, researchers have treated normal organoids with patient serum^513^, used small molecule (Aftin-5) for post-modification of the APP protein^514^, used CRISPR/Cas9 to generate organoids expressing NDs genetic risk factors, or directly created patient-derived organoids^515^. However, they mostly modelled familial cases^516–524^ and comparably few sporadic cases^515,525^.

In the pathophysiological progression of NDs, crosstalk between distinct regions is crucial point to investigate. For such a recapitulation, combining two or more brain region-specific organoids may be useful. By addressing this, Pasca et al. ^526^ first generated combined organoids termed “assembloid” to better recapitulate the regional interactions, including non-neuronal cells, such as microglia and endothelial cells from different embryonic layers. Following this, brain assembloids have been developed for various regions, including the cortical-striatal, cortical-spinal^527^, cortical-perictyes^528^, corticospinal-skeletal muscle^529^, and disease models, such as Timothy disease^530^ and AD^531,532^. Moreover, to overcome the limitation of vascularisation, Kong et al. ^531^ recently developed a fused cortical vessel assembloid treated with Aβ proteins externally. When exposed to SARS-CoV-2 infection, this assembloid showed an increased number of astrocytes and microglia, indicating how systematic inflammation accelerates neuroinflammation in AD. In another study, instead of regional characterisation, Rickner et al. ^532^ generated a neuro-astrocyte assembloid (asteroids) composed of neurons cultured with tau oligomers and astrocytes that showed rapid maturation. Assembloid successfully demonstrated several pathological changes, such as tau misfolding, aggregation, and reactive astrogliosis. When treated with an HSP inhibitor, the pathological and neurodegenerative changes declined.

However, the use of organoid models to study NDs remains controversial for several reasons. The current method for developing organoids has only recently achieved anatomical and functional characterisations closer to those of the foetal neonatal human brain after approximately nine to 10 months of culture^533,534^. Therefore, it is considered more suitable for modelling neurodevelopmental disorders and has been applied to diseases, such as autism spectrum disorder^535^, epilepsy^536^, and microencephaly^25,537^. Nonetheless, encouraging results of organoid maturation have also been observed in terms of cellular composition, anatomical characteristics, such as distinct cortical layers, functional choroid plexus, and brain convolution formation using genetic or mechanical methods^538,539^.

**Fig. 3:**
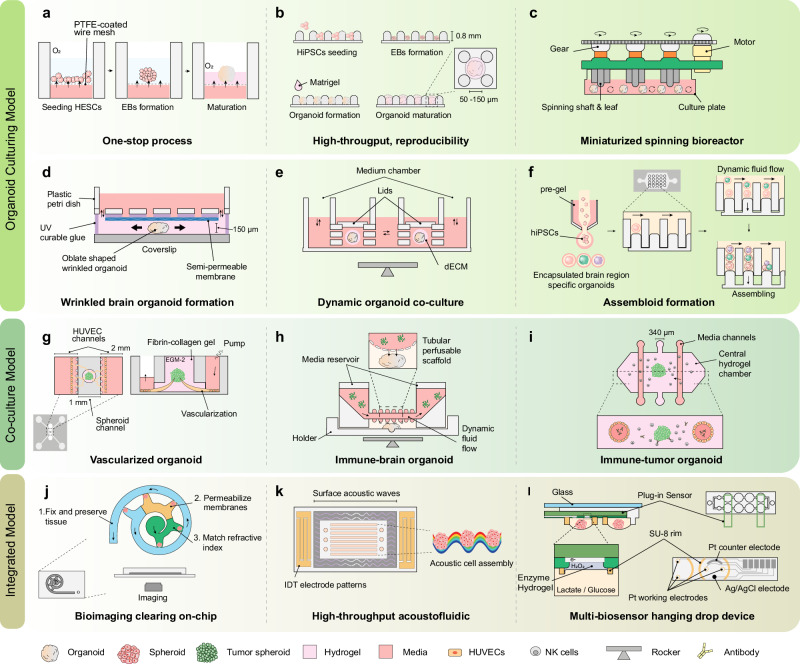
Representative designs of organoid on-a-chip. **a** One-stop microfluidic organoid-culture platform. The four-step organoid maturing process was performed on one platform^540^. **b** High-throughput micropillar array. Brain organoids were directly generated from dissociated hiPSCs in a controllable micropillar array with restrained space in the microarray^541^. **c** Miniaturised multi-well spinning bioreactor device. The device is cost-effective, high throughput, and has increased maturation and reproducibility^542^. **d** With the mechanical stimulation of a microfluidic chip, surface folding appeared in the brain organoid^544^. **e** Organoids in a human brain-derived decellularized ECM gel, under the dynamic condition of a microfluidic device, showed higher survival, and uniformity^50^. **f** Organoids were encapsulated via microfluidic electrospray, and transferred to multi-layered microfluidic chip to form brain-region-specific assembloid^547^. **g** A tumour spheroid was integrated with a perfusable vascular network in a three-line chamber microfluidic chip^548,549^. **h** Neuroimmune interaction of cortical organoid and microglia in a 3D-printed organoid culturing device^550^. **i** NK cell interaction with tumour organoid in a microfluidic chip^552^. **j** Microfluidic device for clearing intact 3D microtissue in 1 d for confocal microscopy^553^. **k** Acoustofluidic chip for forming uniform spheroids in a fast and high-throughput manner^554^. **l** Hanging drop device with plug-in multi biosensors^555^. PTFE polytetrafluoroethylene, EB embryonic bodies, ECM extracellular matrix, HESC human embryonic stem cell, hiPSC human-induced pluripotent stem cell, HUVEC human umbilical vein endothelial cell, IDT interdigital transducer pattern, NK natural killer.

Recently, various organoid culture platforms have been designed with promising results (Fig. 3). These platforms aim to improve in vivo characterisation, maturation, and uniformity of organoids while reducing the culturing time and labour required. They also have the potential to cooperate with immune and vascular components, bioimaging, and biosensors for effective mimicry and analysis^412^.

Conventional organoid protocols involve several steps that are likely to lead to contamination, damage, sample loss, and dissimilarity. This could be solved with a “one-stop” microfluidic platform that enables all steps of organoid culturing and long-term maintaining process, while monitoring and analysing without any manual additional transferring process^540,541^. Ao et al. ^540^ designed a simple device to study the effects of cannabis on prenatal brain organoids (Fig. 3a). This simple “one-stop” chip allows organoids to start from embryonic bodies (EB). With a physical barrier and an air-liquid interface, organoids are prevented from random merging and core necrosis, which leads to better uniformity and maturation. A similar “one-stop” approach has been employed to the micropillar array device, resulting in greater uniformity and reproducibility^541^ (Fig. 3b). However, organoids eventually exhibit nutrient deficiency in their cores. Furthermore, these models should be transferred to separate analysis tools such as MEA because the design does not include integrated analysis tools. Some research groups have used spinning bioreactors to provide an equal distribution of nutrients to cultured organoids^542^. To enhance organoid viability, a dynamic medium flow can be achieved by integrating either a rocking platform or a syringe pump^49,543^. Qian et al. ^542^. developed a 3D-printed bioreactor that resulted in improved cortical characterisation, such as a human-like outer radial glial layer, compared with static conditions (Fig. 3c). Furthermore, microfluidics can help achieve more human-like characteristics, such as surface wrinkles^544^ and functional myelinated axons of peripheral neurons^545^. One group induced mechanical force using a simple chip device to produce wrinkled brain organoids^544^ (Fig. 3d). Compressed organoids are manipulated to an oblate shape with a surface-wrinkling effect that is not observed in conventional methods. They observed contraction of the cytoskeleton in the core and nuclear expansion in the outer area of the brain organoids.

Another crucial component of 3D culture is the surrounding scaffolds. Supporting biomaterials, such as Matrigel, decellularised ECM (dECM), and other biocompatible hydrogels have been used to accelerate maturation and structural development by providing a brain-like environment^50,546^. Cho et al. ^50^ generated organoids with a brain-derived decellularized ECM on a microfluidic-based platform integrated with a rocking platform (Fig. 3e). Compared to the commonly used Matrigel, dECM induces rapid growth and desirable neuron-glia interactions. The microfluidic chip provides an optimal environment for dynamic fluid flow and gas nutrient transfer, thereby accelerating cell viability and growth. Additionally, this platform allows for indirect interactions between organoids which can be applied in co-culture studies of organoids from different regions. There are a few examples of the use of microfluidic chips for assembloid development. However, only recently one group reported a microfluidic-based assembloid-forming device^547^ (Fig. 3f). They designed a three-layered microfluidic chip (micropillar, microhole, and culture top layer) to generate an assembloid by combining encapsulated organoids. However, before loading onto the chip, EB and different brain regional organoids should be separately generated via a microfluidic encapsulation method. The interaction between the cortical-hippocampal-thalamic regions has been well characterised using assembloid modelling.

Vascularisation and immune interactions are critical for further characterisation of brain organoids. Using vasculogenesis- or angiogenesis-inducing factors, some groups have attempted to vascularise 3D organoids using microfluidic platforms. Nashimoto et al. ^548,549^ created a blood vessel network merged with tumour spheroids in a fibroblast hydrogel in the middle chamber by culturing human umbilical vein endothelial cells in two side channels (Fig. 3g). The vascular network exhibited high perfusability and viability. In other designs, instead of following the commonly used closed-well approach, Salmon et al. ^51^ established an open-well organoid chip using 3D printing and successfully generated perfusable neurovascular organoids by simultaneously differentiating iPSCs into vascular and neuronal lineages. One group developed an assembly chip device for the interaction of circulating microglia and brain organoids using a tubular porous scaffold^550^ (Fig. 3h). Moreover, this chip design was repurposed in different studies to recapitulate organoid vascularisation^506,551^. Repurposed chips can also be used in brain studies. Tumour spheroid interactions with natural killer cells were recapitulated in chips with vascular-mimicked channels^552^ (Fig. 3i).

Microfluidic chips can be integrated into bioimaging devices and biosensors for precise monitoring and analysis. However, because of their density and size, 3D cultures have limited imaging properties. One group has proposed on-a-chip clearing techniques for 3D spheroids for better imaging. Sequential clearing steps were performed on the device channels. This approach was 20 times faster than conventional clearing methods^553^ (Fig. 3j). For precise manipulation of 3D cultures, the surface acoustic wave has been applied to the microfluidic chip, which allows high-throughput production of uniform spheroids and assembling spheroids^554^ (Fig. 3k). Also, biosensor-integrated microfluidic chips have also been developed for 3D cultures^555^. This hanging drop chip allowed the monitoring of secreted glucose and lactate levels with electrodes functionalized with oxidase enzymes^555^ (Fig. 3l).

## The outlook of in vitro model in the pharmacological industry

### The challenges in neurodegenerative disease drug development

Despite substantial advances in our understanding of disease mechanisms and technological innovations, the development of effective treatments for NDs remains challenging. Recent breakthroughs have been made in the treatment of AD, including the use of aducanumab^16^, lecanemab^17^, and donanemab^556^. However, the effectiveness of the new drugs only appears at certain stages of AD^116,556^. Moreover, Aβ-against antibodies are controversial in human cognition although it exhibits a considerable decrease of the plaque. While these recent approvals are beneficial in certain groups of patients, there is still much work to be done to expand the effectiveness of these drugs, considering the multifactorial and heterogeneous characteristics of NDs.

In addition, the treatment development process requires a cost-effective and time-saving approach. The AD drug development field has invested approximately $42.5 billion in clinical trials over the past 30 years, which requires a long, dedicated effort, but has yielded few promising results^116,557^. Several factors have been attributed to drug failure, including intervention at an inappropriate stage of disease progression, insufficient biomarkers, and the use of incorrect research models^116^. Specifically, preclinical studies conducted on familial genetically engineered mouse models have limitations as they do not fully replicate all the features of NDs, especially sporadic cases. These models exhibit varied clinical and pathological phenotypes that restrict their effectiveness in translation to human physiology^558^. Moreover, long-term preservation of animals with age-related characteristics can be costly and time-consuming^559^. Translation to a more physiologically relevant, human-cell-sourced, in vitro model that incorporates most features of certain NDs for studying disease mechanisms and developing drugs has the potential to reduce costs and time for development and produce more representative and reliable test results.

### Microfluidic chip in pharmaceutical industry

Over the past two decades, the development of advanced in vitro models has increased in the academic field. However, the pharmaceutical industry has not yet adopted microfluidic chips^560,561^.

Following the recent FDA policy on the shift towards the use of more human-relevant models in drug testing, both academic and pharmaceutical industries have begun to use organ-on-a-chip and organoid-based models^562^. Several large pharmaceutical companies (AstraZeneca, Bayer, and Roche) have been using organoid and microfluidic chips to a limited degree to enhance the screening accuracy of potential drug candidates^561,563^. Moreover, global efforts (USA, Europe, and Japan) have been made to translate academic achievements to the pharmaceutical industry using a standardised and reproducible predictive drug-screening approach^564–566^. Tissue chip testing centres established by the NIH National Center for Advancing Translational Sciences initiated grantees to validate various types of tissue chip platforms for drug testing^566,567^. Under these grants, three independent organisations validated microphysiological systems that replicated the kidney, liver, and BBB for drug transport and toxicity studies, with the goal of increasing the reliability, reproducibility, robustness, and throughput of its effective application^568^.

### Microfluidic chip for drug screening at the academic level

Despite ongoing efforts to address reproducibility and scalability in the pharmaceutical industry, drug screening and toxicity studies of potential ND drug components have also been conducted on-a-chip in the academic field. Numerous companies, such as Emulate, TissUse, Mimetas, Nortis, and CN Bio offer various designs of microfluidic chips to researchers for conduct physiologically relevant studies of disease mechanism, drug screening, and toxicity^563^.

Some research groups have performed drug screening and toxicity studies on previously rejected drug candidates using microfluidic chips. Clinical trials on ASOs to prevent and alleviate the progression of AD, PD, ALS, and HD are currently underway. However, these clinical trials demonstrated toxicity. Nieskens et al. ^569^ verified ASOs toxicity by testing varying doses on a kidney proximal tubule-on-a-chip using human renal proximal tubule epithelial cells. In addition, one group reported the dose-dependent efficacy of NMJ-blocking drugs for ALS using a commonly used micro-channelled two-compartment design. This chip allows the interaction between motor neurons and muscles only through the axons. Functional ability was revealed by muscle contraction when motor neurons were stimulated using silver electrodes. They tested the several doses of botulinum neurotoxin, Curare, and α-bungarotoxin (α-BTX), and quantified the dose-dependent NMJ dysfunction as dose-response curve^570^.

Monolayer culture approaches have often been applied to drug discovery and preclinical screening. However, as described in the previous section, 3D cell culture is expected to provide closer in vivo characteristics, increasing the predictability of clinical results. In addition, replacing animal-based drug screening with patient-origin organoids is considered a substantial development in the pharmaceutical field which will increase human reliability and reduce costs and ethical concerns by overcoming the drawbacks of animal models. Recently, several attempts have been made to apply organoids in drug discovery studies in an academic field. Osaki et al. ^506^ tested the muscle atrophy-reversing effect of bosutinib and rapamycin on spheroids on chips, confirming drug efficacy with caspase-3/7 staining at day 14. Moreover, neuroprotective treatments positively influenced the optogenetic stimulation of muscle contraction. This chip design is beneficial because it evaluates not only the single or combined drug effects on functional 3D co-cultures, but also drug delivery across the EC barrier. This chip design significantly increased throughput by simultaneously screening single and combined drugs. Park et al. ^515^. introduced a novel organoid-culture drug-screening platform that employed mathematical network analysis. Researchers have investigated the effects of current FDA-approved drug candidates on AD patient-origin cerebral organoids and confirmed the structure, viability, and Aβ and tau depositions. This study demonstrates the potential of patient-origin organoids for drug screening of NDs.

### Potentials of advanced microfluidic chip in pharmaceutical industry

**Fig. 4:**
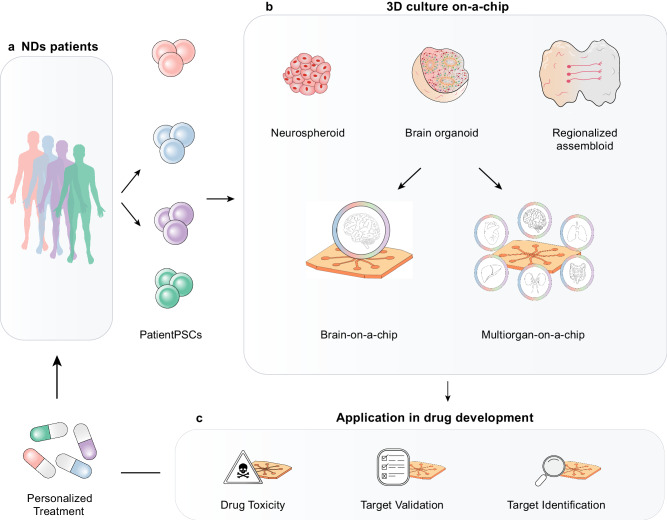
The future outlook of NDs-on-a-chip for drug development. **a** Patient-derived pluripotent stem cells can provide relevant insights into ND pathophysiology. **b** 3D culture-on-a-chip combines the in vivo relevant characteristics of patient-derived 3D cultures (neurospheroids, organoids, and assembloids) and the unique engineering possibilities of microfluidic chips that can model interconnected brain regions and multi-organs. **c** Microfluidic-based, patient-derived in vitro ND models will contribute to drug discovery and preclinical drug development studies by replacing the animal models with the promise of personalised treatment. ND neurodegenerative disease.

Through collaborative global efforts, the integration of microfluidic chips with advanced technologies has the potential to revolutionise the pharmaceutical industry. The organoid-on-a-chip and multiorgan-on-a-chip systems can play crucial roles in various stages of drug discovery, ranging from target identification to preclinical screening (Fig. 4). In addition, we believe that such an advanced model can be useful even in the clinical stage when deciding on the effective DMT for each patient subtype by sourcing patient-derived cells. However, to achieve this, many crucial industrial and technological challenges must be considered, such as reliability, robustness, reproducibility, compatibility with analysis techniques, material choice, scalability, and standardisation. Moreover, there is a great need for defined metrics for validation and benchmarks for modelling each ND.

An advanced on-a-chip system has the capability to not only validate the effects of drugs on neuropathological features, but also measure functional changes through real-time electrophysiological measurements. Efforts have been made to measure brain waves in real time from brain organoids using invasive and non-invasive electrophysiological biosensors integrated on a high-throughput microfluidic chip^534^. This integration will provide a clinically relevant assessment of NDs. Moreover, once drug candidates are identified for NDs, their mechanism of action, safety, dose-response relationship, potential side effects, and pharmacokinetics can be tested on even more complex inclusive systems, such as multiorgan-on-a-chip platforms (composed of the brain, kidney, liver, gut, and heart)^571^. The use of multiorgan-on-a-chip models reduces the dependence on animal models during this stage, while potentially providing more representative, cost-effective, and accurate results, thereby facilitating successful clinical testing. In addition, patient-derived cell sources can facilitate the development of effective treatments for certain patient subsets. Advanced on-a-chip systems could be beneficial for patient stratification testing to identify effective DMT with fewer side effects, especially in challenging multifactorial diseases, such as AD^572^.

In the future, microfluidic chips will substantially contribute to the translation of brain organoids and assembloids for the development of ND drug discovery and personalised medicine. Notably, in the development of microfluidic chips, there are biological, technical, and commercial challenges for the entire application to the pharmaceutical industry and personalised medicine^573^. Despite these hurdles, we believe that advanced microfluidic chips and molecular biophysical techniques will advance our understanding of the complexities of ND pathogenesis and challenge our dependence on animal-based tools.

## References

[1] United Nations, Department of Economic and Social Affairs, Population Division (2019). *World Population Prospects 2019: Highlights* (ST/ESA/SER.A/423). (United Nations, 2019).

[2] Nichols E (2022). Estimation of the global prevalence of dementia in 2019 and forecasted prevalence in 2050: an analysis for the Global Burden of Disease Study 2019. Lancet Public Health.

[3] Deuschl G (2020). The burden of neurological diseases in Europe: an analysis for the Global Burden of Disease Study 2017. Lancet Public Health.

[4] Wang Y (2023). Burden of common neurologic diseases in Asian Countries, 1990–2019. An Analysis for the Global Burden of Disease Study 2019. Neurology.

[5] Erkkinen, M. G., Kim, M. O. & Geschwind, M. D. Clinical neurology and epidemiology of the major neurodegenerative diseases. *Cold Spring Harb. Perspect. Biol.*10.1101/cshperspect.a033118 (2018). 10.1101/cshperspect.a033118 PMC5880171 28716886

[6] Poewe W (2017). Parkinson disease. Nat. Rev. Dis. Prim..

[7] Kiernan MC (2011). Amyotrophic lateral sclerosis. Lancet.

[8] Chaganti SS, McCusker EA, Loy CT (2017). What do we know about late onset Huntington’s disease?. J. Huntingt. Dis..

[9] Knopman DS (2021). Alzheimer disease. Nat. Rev. Dis. Prim..

[10] Masters CL (2015). Alzheimer’s disease. Nat. Rev. Dis. Prim..

[11] Przedborski S (2017). The two-century journey of Parkinson disease research. Nat. Rev. Neurosci..

[12] Taylor JP, Brown RH, Cleveland DW (2016). Decoding ALS: from genes to mechanism. Nature.

[13] Bates GP (2015). Huntington disease. Nat. Rev. Dis. Prim..

[14] Gan L, Cookson MR, Petrucelli L, La Spada AR (2018). Converging pathways in neurodegeneration, from genetics to mechanisms. Nat. Neurosci..

[15] Peng C, Trojanowski JQ, Lee VM (2020). Protein transmission in neurodegenerative disease. Nat. Rev. Neurol..

[16] Cummings J (2021). Aducanumab produced a clinically meaningful benefit in association with amyloid lowering. Alzheimers Res. Ther..

[17] van Dyck CH (2022). Lecanemab in early Alzheimer’s disease. N. Engl. J. Med..

[18] Gurney ME, Fleck TJ, Himes CS, Hall ED (1998). Riluzole preserves motor function in a transgenic model of familial amyotrophic lateral sclerosis. Neurology.

[19] Ito H (2008). Treatment with edaravone, initiated at symptom onset, slows motor decline and decreases SOD1 deposition in ALS mice. Exp. Neurol..

[20] Bjornson-Hooper, Z. B. et al. A Comprehensive Atlas of Immunological Differences Between Humans, Mice, and Non-Human Primates. *Front. Immunol.*10.3389/fimmu.2022.867015 (2022). 10.3389/fimmu.2022.867015 PMC8962947 35359965

[21] Hodge RD (2019). Conserved cell types with divergent features in human versus mouse cortex. Nature.

[22] Bang S, Jeong S, Choi N, Kim HN (2019). Brain-on-a-chip: a history of development and future perspective. Biomicrofluidics.

[23] Bertram L, Tanzi RE (2005). The genetic epidemiology of neurodegenerative disease. J. Clin. Investig..

[24] Loewa A, Feng JJ, Hedtrich S (2023). Human disease models in drug development. Nat. Rev. Bioeng..

[25] Lancaster MA (2013). Cerebral organoids model human brain development and microcephaly. Nature.

[26] Sakaguchi H (2015). Generation of functional hippocampal neurons from self-organizing human embryonic stem cell-derived dorsomedial telencephalic tissue. Nat. Commun..

[27] Qian X (2018). Generation of human brain region–specific organoids using a miniaturized spinning bioreactor. Nat. Protoc..

[28] Jo J (2016). Midbrain-like organoids from human pluripotent stem cells contain functional dopaminergic and neuromelanin-producing neurons. Cell Stem Cell.

[29] Pellegrini L (2020). Human CNS barrier-forming organoids with cerebrospinal fluid production. Science.

[30] Muguruma K, Nishiyama A, Kawakami H, Hashimoto K, Sasai Y (2015). Self-organization of polarized cerebellar tissue in 3D culture of human pluripotent stem cells. Cell Rep..

[31] Kim J, Koo B-K, Knoblich JA (2020). Human organoids: model systems for human biology and medicine. Nat. Rev. Mol. Cell Biol..

[32] Hofer M, Lutolf MP (2021). Engineering organoids. Nat. Rev. Mater..

[33] Park JH, Byeun DG, Choi JK (2022). Progress, prospects, and limitations of organoid technology. Organoid.

[34] Ingber DE (2022). Human organs-on-chips for disease modelling, drug development and personalized medicine. Nat. Rev. Genet..

[35] Mou L (2022). Integrated biosensors for monitoring microphysiological systems. Lab Chip.

[36] Taylor AM, Dieterich DC, Ito HT, Kim SA, Schuman EM (2010). Microfluidic local perfusion chambers for the visualization and manipulation of synapses. Neuron.

[37] Park, J., Koito, H., Li, J. & Han, A. A multi-compartment CNS neuron-glia Co-culture microfluidic platform. *J. Vis. Exp.*10.3791/1399 (2009). 10.3791/1399 PMC2774404 19745806

[38] Park T-E (2019). Hypoxia-enhanced blood-brain barrier chip recapitulates human barrier function and shuttling of drugs and antibodies. Nat. Commun..

[39] Kawakita S (2022). Organ-on-a-chip models of the blood–brain barrier: recent advances and future prospects. Small.

[40] Lyu Z (2021). A neurovascular-unit-on-a-chip for the evaluation of the restorative potential of stem cell therapies for ischaemic stroke. Nat. Biomed. Eng..

[41] Yi H-G (2019). A bioprinted human-glioblastoma-on-a-chip for the identification of patient-specific responses to chemoradiotherapy. Nat. Biomed. Eng..

[42] Yap, Y. C., Dickson, T. C., King, A. E., Breadmore, M. C. & Guijt, R. M. In *Stem Cell Technologies in Neuroscience. Neuromethods* (eds Srivastava, A. K., Snyder, E. Y. & Teng, Y. D.) Ch. 10, 145–156 (Springer, New York, 2017).

[43] Pediaditakis I (2022). A microengineered Brain-Chip to model neuroinflammation in humans. iScience.

[44] Amirifar L (2022). Brain-on-a-chip: recent advances in design and techniques for microfluidic models of the brain in health and disease. Biomaterials.

[45] Haque MR (2022). Patient-derived pancreatic cancer-on-a-chip recapitulates the tumor microenvironment. Microsyst. Nanoeng..

[46] Wang Y (2020). Modeling Human Nonalcoholic Fatty Liver Disease (NAFLD) with an organoids-on-a-chip system. ACS Biomater. Sci. Eng..

[47] Homan KA (2019). Flow-enhanced vascularization and maturation of kidney organoids in vitro. Nat. Methods.

[48] Lee KK (2018). Human stomach-on-a-chip with luminal flow and peristaltic-like motility. Lab Chip.

[49] Wang Y, Wang L, Zhu Y, Qin J (2018). Human brain organoid-on-a-chip to model prenatal nicotine exposure. Lab Chip.

[50] Cho A-N (2021). Microfluidic device with brain extracellular matrix promotes structural and functional maturation of human brain organoids. Nat. Commun..

[51] Salmon I (2022). Engineering neurovascular organoids with 3D printed microfluidic chips. Lab Chip.

[52] 2023 Alzheimer’s disease facts and figures. *Alzheimers Dement.* **19**, 1598–1695 (2023). 10.1002/alz.13016 36918389

[53] Bature F, Guinn B-A, Pang D, Pappas Y (2017). Signs and symptoms preceding the diagnosis of Alzheimer’s disease: a systematic scoping review of literature from 1937 to 2016. BMJ Open.

[54] Rocca WA, Amaducci LA, Schoenberg BS (1986). Epidemiology of clinically diagnosed Alzheimer’s disease. Ann. Neurol..

[55] Jansen WJ (2015). Prevalence of cerebral amyloid pathology in persons without dementia: a meta-analysis. Jama.

[56] Serrano-Pozo A, Frosch MP, Masliah E, Hyman BT (2011). Neuropathological alterations in Alzheimer disease. Cold Spring Harb. Perspect. Med..

[57] Kang J (1987). The precursor of Alzheimer’s disease amyloid A4 protein resembles a cell-surface receptor. Nature.

[58] Sherrington R (1995). Cloning of a gene bearing missense mutations in early-onset familial Alzheimer’s disease. Nature.

[59] Levy-Lahad E (1995). Candidate gene for the chromosome 1 familial Alzheimer’s disease locus. Science.

[60] Schwartzentruber J (2021). Genome-wide meta-analysis, fine-mapping and integrative prioritization implicate new Alzheimer’s disease risk genes. Nat. Genet..

[61] Wightman DP (2021). A genome-wide association study with 1,126,563 individuals identifies new risk loci for Alzheimer’s disease. Nat. Genet..

[62] Yamazaki Y, Zhao N, Caulfield TR, Liu CC, Bu G (2019). Apolipoprotein E and Alzheimer disease: pathobiology and targeting strategies. Nat. Rev. Neurol..

[63] Guerreiro R (2012). TREM2 variants in Alzheimer’s disease. N. Engl. J. Med..

[64] Jonsson T (2013). Variant of TREM2 associated with the risk of Alzheimer’s disease. N. Engl. J. Med..

[65] Giau VV (2019). Genetic analyses of early-onset Alzheimer’s disease using next generation sequencing. Sci. Rep..

[66] Mendez MF (2017). Early-onset Alzheimer disease. Neurol. Clin..

[67] Guo Q, Wang Z, Li H, Wiese M, Zheng H (2012). APP physiological and pathophysiological functions: insights from animal models. Cell Res..

[68] Hardy JA, Higgins GA (1992). Alzheimer’s disease: the amyloid cascade hypothesis. Science.

[69] Liu Y (2021). Roles and mechanisms of the protein quality control system in Alzheimer’s disease. Int. J. Mol. Sci..

[70] Hou H (2017). Low-density lipoprotein receptor-related protein-1 (LRP1) C4408R mutant promotes amyloid precursor protein (APP) α-cleavage in vitro. Neuromol. Med..

[71] Okada H (2010). Proteomic identification of sorting nexin 6 as a negative regulator of BACE1-mediated APP processing. FASEB J..

[72] Kim W (2018). BACE1 elevation engendered by GGA3 deletion increases β-amyloid pathology in association with APP elevation and decreased CHL1 processing in 5XFAD mice. Mol. Neurodegener..

[73] Andersen OM (2006). Molecular dissection of the interaction between amyloid precursor protein and its neuronal trafficking receptor SorLA/LR11. Biochemistry.

[74] Lambert MP (1998). Diffusible, nonfibrillar ligands derived from Abeta1-42 are potent central nervous system neurotoxins. Proc. Natl Acad. Sci. USA.

[75] Lacor PN (2004). Synaptic targeting by Alzheimer’s-related amyloid beta oligomers. J. Neurosci..

[76] Younan ND, Chen K-F, Rose R-S, Crowther DC, Viles JH (2018). Prion protein stabilizes amyloid-β (Aβ) oligomers and enhances Aβ neurotoxicity in a Drosophila model of Alzheimer’s disease. J. Biol. Chem..

[77] Liang J, Kulasiri D, Samarasinghe S (2017). Computational investigation of Amyloid-β-induced location- and subunit-specific disturbances of NMDAR at hippocampal dendritic spine in Alzheimer’s disease. PLoS ONE.

[78] Wang D (2013). β2 adrenergic receptor, protein kinase A (PKA) and c-Jun N-terminal kinase (JNK) signaling pathways mediate tau pathology in Alzheimer disease models. J. Biol. Chem..

[79] Shen L-L (2019). Neurotrophin receptor p75 mediates amyloid β-induced tau pathology. Neurobiol. Dis..

[80] Bencherif M, Lippiello PM (2010). Alpha7 neuronal nicotinic receptors: the missing link to understanding Alzheimer’s etiopathology?. Med. Hypotheses.

[81] Bode DC, Baker MD, Viles JH (2017). Ion channel formation by amyloid-β42 oligomers but not amyloid-β40 in cellular membranes. J. Biol. Chem..

[82] Wang H (2020). Parkin overexpression attenuates Aβ-induced mitochondrial dysfunction in HEK293 cells by restoring impaired mitophagy. Life Sci..

[83] Sun JL (2019). Co-activation of selective nicotinic acetylcholine receptors is required to reverse beta amyloid–induced Ca2+ hyperexcitation. Neurobiol. Aging.

[84] Zhang F (2020). β-amyloid redirects norepinephrine signaling to activate the pathogenic GSK3β/tau cascade. Sci. Transl. Med..

[85] Thal DR, Rüb U, Orantes M, Braak H (2002). Phases of Aβ-deposition in the human brain and its relevance for the development of AD. Neurology.

[86] Pignataro A, Middei S (2017). Trans-Synaptic Spread of Amyloid-β in Alzheimer’s Disease: Paths to β-Amyloidosis. Neural Plast..

[87] Jucker M, Walker LC (2013). Self-propagation of pathogenic protein aggregates in neurodegenerative diseases. Nature.

[88] Olsson TT, Klementieva O, Gouras GK (2018). Prion-like seeding and nucleation of intracellular amyloid-β. Neurobiol. Dis..

[89] Cohen SIA (2013). Proliferation of amyloid-β42 aggregates occurs through a secondary nucleation mechanism. Proc. Natl Acad. Sci. USA.

[90] Sowade RF, Jahn TR (2017). Seed-induced acceleration of amyloid-β mediated neurotoxicity in vivo. Nat. Commun..

[91] Nath S (2012). Spreading of neurodegenerative pathology via neuron-to-neuron transmission of β-amyloid. J. Neurosci..

[92] Sardar Sinha M (2018). Alzheimer’s disease pathology propagation by exosomes containing toxic amyloid-beta oligomers. Acta Neuropathol..

[93] Rajendran L (2006). Alzheimer’s disease β-amyloid peptides are released in association with exosomes. Proc. Natl Acad. Sci. USA.

[94] Lim CZJ (2019). Subtyping of circulating exosome-bound amyloid β reflects brain plaque deposition. Nat. Commun..

[95] Wang Y, Cui J, Sun X, Zhang Y (2011). Tunneling-nanotube development in astrocytes depends on p53 activation. Cell Death Differ..

[96] Zhang K (2021). Intercellular transport of Tau protein and β-amyloid mediated by tunneling nanotubes. Am. J. Transl. Res..

[97] Valappil, D. K., Mini, N. J., Dilna, A. & Nath, S. Membrane interaction to intercellular spread of pathology in Alzheimer’s disease. *Front. Neurosci.*10.3389/fnins.2022.936897 (2022). 10.3389/fnins.2022.936897 PMC9500529 36161178

[98] Arriagada PV, Growdon JH, Hedley-Whyte ET, Hyman BT (1992). Neurofibrillary tangles but not senile plaques parallel duration and severity of Alzheimer’s disease. Neurology.

[99] Gong CX, Liu F, Grundke-Iqbal I, Iqbal K (2005). Post-translational modifications of tau protein in Alzheimer’s disease. J. Neural Transm..

[100] Alonso ADC, Zaidi T, Grundke-Iqbal I, Iqbal K (1994). Role of abnormally phosphorylated tau in the breakdown of microtubules in Alzheimer disease. Proc. Natl Acad. Sci. USA.

[101] Schneider A, Biernat J, von Bergen M, Mandelkow E, Mandelkow EM (1999). Phosphorylation that detaches tau protein from microtubules (Ser262, Ser214) also protects it against aggregation into Alzheimer paired helical filaments. Biochemistry.

[102] Strang KH (2019). Phosphorylation of serine 305 in tau inhibits aggregation. Neurosci. Lett..

[103] Stefanoska K (2022). Alzheimer’s disease: ablating single master site abolishes tau hyperphosphorylation. Sci. Adv..

[104] Patrick GN (1999). Conversion of p35 to p25 deregulates Cdk5 activity and promotes neurodegeneration. Nature.

[105] Mazanetz MP, Fischer PM (2007). Untangling tau hyperphosphorylation in drug design for neurodegenerative diseases. Nat. Rev. Drug Discov..

[106] Goedert M, Jakes R, Qi Z, Wang JH, Cohen P (1995). Protein phosphatase 2A is the major enzyme in brain that dephosphorylates tau protein phosphorylated by proline-directed protein kinases or cyclic AMP-dependent protein kinase. J. Neurochem..

[107] Liu F, Grundke-Iqbal I, Iqbal K, Gong CX (2005). Contributions of protein phosphatases PP1, PP2A, PP2B and PP5 to the regulation of tau phosphorylation. Eur. J. Neurosci..

[108] Pao P-C (2023). A Cdk5-derived peptide inhibits Cdk5/p25 activity and improves neurodegenerative phenotypes. Proc. Natl Acad. Sci. USA.

[109] Clavaguera F (2009). Transmission and spreading of tauopathy in transgenic mouse brain. Nat. Cell Biol..

[110] Wang Y (2017). The release and trans-synaptic transmission of Tau via exosomes. Mol. Neurodegener..

[111] Tardivel M (2016). Tunneling nanotube (TNT)-mediated neuron-to neuron transfer of pathological Tau protein assemblies. Acta Neuropathol. Commun..

[112] Asai H (2015). Depletion of microglia and inhibition of exosome synthesis halt tau propagation. Nat. Neurosci..

[113] Gabrielli M (2022). Microglial large extracellular vesicles propagate early synaptic dysfunction in Alzheimer’s disease. Brain.

[114] Chen G-F (2017). Amyloid beta: structure, biology and structure-based therapeutic development. Acta Pharmacol. Sin..

[115] Sanchez JS (2021). The cortical origin and initial spread of medial temporal tauopathy in Alzheimer’s disease assessed with positron emission tomography. Sci. Transl. Med..

[116] Boxer AL, Sperling R (2023). Accelerating Alzheimer’s therapeutic development: the past and future of clinical trials. Cell.

[117] Ittner LM, Götz J (2011). Amyloid-β and tau — a toxic pas de deux in Alzheimer’s disease. Nat. Rev. Neurosci..

[118] Muralidar S, Ambi SV, Sekaran S, Thirumalai D, Palaniappan B (2020). Role of tau protein in Alzheimer’s disease: the prime pathological player. Int J. Biol. Macromol..

[119] Shin WS (2019). Amyloid β-protein oligomers promote the uptake of tau fibril seeds potentiating intracellular tau aggregation. Alzheimers Res. Ther..

[120] Busche MA, Hyman BT (2020). Synergy between amyloid-β and tau in Alzheimer’s disease. Nat. Neurosci..

[121] Lauritzen I (2016). Intraneuronal aggregation of the β-CTF fragment of APP (C99) induces Aβ-independent lysosomal-autophagic pathology. Acta Neuropathol..

[122] Pulina, M. V., Hopkins, M., Haroutunian, V., Greengard, P. & Bustos, V. C99 selectively accumulates in vulnerable neurons in Alzheimer’s disease. *Alzheimers Dement.*10.1016/j.jalz.2019.09.002 (2019). 10.1016/j.jalz.2019.09.002 31677937

[123] Walker KA, Ficek BN, Westbrook R (2019). Understanding the role of systemic inflammation in Alzheimer’s disease. ACS Chem. Neurosci..

[124] Taipa R (2019). Proinflammatory and anti-inflammatory cytokines in the CSF of patients with Alzheimer’s disease and their correlation with cognitive decline. Neurobiol. Aging.

[125] Serrano-Pozo A (2011). Reactive glia not only associates with plaques but also parallels tangles in Alzheimer’s disease. Am. J. Pathol..

[126] Ries, M. & Sastre, M. Mechanisms of Aβ clearance and degradation by glial cells. *Front. Aging Neurosci.*10.3389/fnagi.2016.00160 (2016). 10.3389/fnagi.2016.00160 PMC4932097 27458370

[127] Ising C (2019). NLRP3 inflammasome activation drives tau pathology. Nature.

[128] Venegas C (2017). Microglia-derived ASC specks cross-seed amyloid-β in Alzheimer’s disease. Nature.

[129] Efthymiou AG, Goate AM (2017). Late onset Alzheimer’s disease genetics implicates microglial pathways in disease risk. Mol. Neurodegener..

[130] Kleinberger G (2014). TREM2 mutations implicated in neurodegeneration impair cell surface transport and phagocytosis. Sci. Transl. Med..

[131] Jiang T (2014). Upregulation of TREM2 ameliorates neuropathology and rescues spatial cognitive impairment in a transgenic mouse model of Alzheimer’s disease. Neuropsychopharmacology.

[132] Ulland TK, Colonna M (2018). TREM2 — a key player in microglial biology and Alzheimer disease. Nat. Rev. Neurol..

[133] Bailey CC, DeVaux LB, Farzan M (2015). The triggering receptor expressed on myeloid cells 2 binds apolipoprotein E. J. Biol. Chem..

[134] Yeh FL, Wang Y, Tom I, Gonzalez LC, Sheng M (2016). TREM2 binds to apolipoproteins, including APOE and CLU/APOJ, and thereby facilitates uptake of amyloid-beta by microglia. Neuron.

[135] Gratuze M (2023). TREM2-independent microgliosis promotes tau-mediated neurodegeneration in the presence of ApoE4. Neuron.

[136] van Lengerich B (2023). A TREM2-activating antibody with a blood–brain barrier transport vehicle enhances microglial metabolism in Alzheimer’s disease models. Nat. Neurosci..

[137] Rodríguez JJ, Olabarria M, Chvatal A, Verkhratsky A (2009). Astroglia in dementia and Alzheimer’s disease. Cell Death Differ..

[138] Ding Z-B (2021). Astrocytes: a double-edged sword in neurodegenerative diseases. Neural Regener. Res..

[139] Ferrari-Souza JP (2022). Astrocyte biomarker signatures of amyloid-β and tau pathologies in Alzheimer’s disease. Mol. Psychiatry.

[140] Mathys H (2019). Single-cell transcriptomic analysis of Alzheimer’s disease. Nature.

[141] Simonovitch S (2016). Impaired Autophagy in APOE4 Astrocytes. J. Alzheimers Dis..

[142] Zhao J (2017). APOE ε4/ε4 diminishes neurotrophic function of human iPSC-derived astrocytes. Hum. Mol. Genet..

[143] Lee SI (2021). APOE4-carrying human astrocytes oversupply cholesterol to promote neuronal lipid raft expansion and Aβ generation. Stem Cell Rep..

[144] Blanchard JW (2022). APOE4 impairs myelination via cholesterol dysregulation in oligodendrocytes. Nature.

[145] Lin YT (2018). APOE4 causes widespread molecular and cellular alterations associated with Alzheimer’s disease phenotypes in human iPSC-derived brain cell types. Neuron.

[146] Koutsodendris N (2023). Neuronal APOE4 removal protects against tau-mediated gliosis, neurodegeneration and myelin deficits. Nat. Aging.

[147] Habib N (2020). Disease-associated astrocytes in Alzheimer’s disease and aging. Nat. Neurosci..

[148] Keren-Shaul H (2017). A unique microglia type associated with restricting development of Alzheimer’s disease. Cell.

[149] Zeng H (2023). Integrative in situ mapping of single-cell transcriptional states and tissue histopathology in a mouse model of Alzheimer’s disease. Nat. Neurosci..

[150] Nation DA (2019). Blood–brain barrier breakdown is an early biomarker of human cognitive dysfunction. Nat. Med..

[151] Sweeney MD, Sagare AP, Zlokovic BV (2018). Blood–brain barrier breakdown in Alzheimer disease and other neurodegenerative disorders. Nat. Rev. Neurol..

[152] Montagne A, Zhao Z, Zlokovic BV (2017). Alzheimer’s disease: a matter of blood–brain barrier dysfunction?. J. Exp. Med..

[153] Bennett RE (2018). Tau induces blood vessel abnormalities and angiogenesis-related gene expression in P301L transgenic mice and human Alzheimer’s disease. Proc. Natl Acad. Sci. USA.

[154] Armulik A (2010). Pericytes regulate the blood-brain barrier. Nature.

[155] Song J, Choi S-M, Whitcomb DJ, Kim BC (2017). Adiponectin controls the apoptosis and the expression of tight junction proteins in brain endothelial cells through AdipoR1 under beta amyloid toxicity. Cell Death Dis..

[156] Liu C-C (2020). Tau and apolipoprotein E modulate cerebrovascular tight junction integrity independent of cerebral amyloid angiopathy in Alzheimer’s disease. Alzheimers Dement..

[157] Wang Y (2014). Interleukin-1β induces blood–brain barrier disruption by downregulating sonic hedgehog in astrocytes. PLoS ONE.

[158] Iliff JJ (2012). A paravascular pathway facilitates CSF flow through the brain parenchyma and the clearance of interstitial solutes, including amyloid β. Sci. Transl. Med..

[159] Xu Z (2015). Deletion of aquaporin-4 in APP/PS1 mice exacerbates brain Aβ accumulation and memory deficits. Mol. Neurodegener..

[160] Feng W (2020). Microglia prevent beta-amyloid plaque formation in the early stage of an Alzheimer’s disease mouse model with suppression of glymphatic clearance. Alzheimers Res. Ther..

[161] Ishida, K. et al. Glymphatic system clears extracellular tau and protects from tau aggregation and neurodegeneration. *J. Exp. Med*. 10.1084/jem.20211275 (2022). 10.1084/jem.20211275 PMC8932543 35212707

[162] Iliff J, Simon M (2019). CrossTalk proposal: the glymphatic system supports convective exchange of cerebrospinal fluid and brain interstitial fluid that is mediated by perivascular aquaporin-4. J. Physiol..

[163] Salman MM (2021). Emerging roles for dynamic aquaporin-4 subcellular relocalization in CNS water homeostasis. Brain.

[164] Zeppenfeld DM (2017). Association of perivascular localization of aquaporin-4 with cognition and Alzheimer disease in aging brains. JAMA Neurol..

[165] Simon MJ (2018). Transcriptional network analysis of human astrocytic endfoot genes reveals region-specific associations with dementia status and tau pathology. Sci. Rep..

[166] Pedersen TJ, Keil SA, Han W, Wang MX, Iliff JJ (2023). The effect of aquaporin-4 mis-localization on Aβ deposition in mice. Neurobiol. Dis..

[167] Kitchen P (2020). Targeting aquaporin-4 subcellular localization to treat central nervous system edema. Cell.

[168] Sylvain NJ (2021). The effects of trifluoperazine on brain edema, aquaporin-4 expression and metabolic markers during the acute phase of stroke using photothrombotic mouse model. Biochim. Biophys. Acta Biomembr..

[169] Murray ER, Kemp M, Nguyen TT (2022). The microbiota–gut–brain axis in Alzheimer’s disease: a review of taxonomic alterations and potential avenues for interventions. Arch. Clin. Neuropsychol..

[170] Vogt NM (2017). Gut microbiome alterations in Alzheimer’s disease. Sci. Rep..

[171] Chandra S, Sisodia SS, Vassar RJ (2023). The gut microbiome in Alzheimer’s disease: what we know and what remains to be explored. Mol. Neurodegener..

[172] Kumar DKV (2016). Amyloid-β peptide protects against microbial infection in mouse and worm models of Alzheimer’s disease. Sci. Transl. Med..

[173] Seo D-O (2023). ApoE isoform and microbiota-dependent progression of neurodegeneration in a mouse model of tauopathy. Science.

[174] Gao L, Zhang Y, Sterling K, Song W (2022). Brain-derived neurotrophic factor in Alzheimer’s disease and its pharmaceutical potential. Transl. Neurodegener..

[175] Zimbone, S. et al. Amyloid beta monomers regulate cyclic adenosine monophosphate response element binding protein functions by activating type-1 insulin-like growth factor receptors in neuronal cells. *Aging Cell*10.1111/acel.12684 (2018). 10.1111/acel.12684 PMC5770784 29094448

[176] Jiao SS (2016). Brain-derived neurotrophic factor protects against tau-related neurodegeneration of Alzheimer’s disease. Transl. Psychiatry.

[177] Mary A, Eysert F, Checler F, Chami M (2023). Mitophagy in Alzheimer’s disease: molecular defects and therapeutic approaches. Mol. Psychiatry.

[178] Roca-Agujetas V (2021). Cholesterol alters mitophagy by impairing optineurin recruitment and lysosomal clearance in Alzheimer’s disease. Mol. Neurodegener..

[179] Lee S-E (2022). Accumulation of APP-CTF induces mitophagy dysfunction in the iNSCs model of Alzheimer’s disease. Cell Death Discov..

[180] Schmukler E (2020). Altered mitochondrial dynamics and function in APOE4-expressing astrocytes. Cell Death Dis..

[181] Saxena S, Caroni P (2011). Selective neuronal vulnerability in neurodegenerative diseases: from stressor thresholds to degeneration. Neuron.

[182] Rexach J, Geschwind D (2020). Selective neuronal vulnerability in Alzheimer’s disease: a modern holy grail. Neuron.

[183] Roussarie J-P (2020). Selective neuronal vulnerability in Alzheimer’s disease: a network-based analysis. Neuron.

[184] Wang X, Michaelis EK (2010). Selective neuronal vulnerability to oxidative stress in the brain. Front. Aging Neurosci..

[185] Fu H, Hardy J, Duff KE (2018). Selective vulnerability in neurodegenerative diseases. Nat. Neurosci..

[186] Zhang X (2023). Midlife lipid and glucose levels are associated with Alzheimer’s disease. Alzheimers Dement..

[187] Lazar AN (2013). Time-of-flight secondary ion mass spectrometry (TOF-SIMS) imaging reveals cholesterol overload in the cerebral cortex of Alzheimer disease patients. Acta Neuropathol..

[188] Mosconi L (2005). Brain glucose metabolism in the early and specific diagnosis of Alzheimer’s disease. Eur. J. Nucl. Med. Mol. Imaging.

[189] Duran-Aniotz C, Hetz C (2016). Glucose metabolism: a sweet relief of Alzheimer’s disease. Curr. Biol..

[190] Niccoli T (2016). Increased glucose transport into neurons rescues Aβ toxicity in drosophila. Curr. Biol..

[191] Willette AA (2015). Association of insulin resistance with cerebral glucose uptake in late middle–aged adults at risk for Alzheimer disease. JAMA Neurol..

[192] Claxton A (2013). Sex and ApoE genotype differences in treatment response to two doses of intranasal insulin in adults with mild cognitive impairment or Alzheimer’s disease. J. Alzheimers Dis..

[193] Kyrtata N, Emsley HCA, Sparasci O, Parkes LM, Dickie BR (2021). A systematic review of glucose transport alterations in Alzheimer’s disease. Front Neurosci..

[194] Winkler EA (2015). GLUT1 reductions exacerbate Alzheimer’s disease vasculo-neuronal dysfunction and degeneration. Nat. Neurosci..

[195] Foley P (2010). Lipids in Alzheimer’s disease: a century-old story. Biochim. Biophys. Acta.

[196] Hicks, D., Nalivaeva, N. & Turner, A. Lipid rafts and Alzheimer’s disease: protein-lipid interactions and perturbation of signaling. *Front. Physiol.*10.3389/fphys.2012.00189 (2012). 10.3389/fphys.2012.00189 PMC3381238 22737128

[197] Feringa, F. M. & van der Kant, R. Cholesterol and Alzheimer’s disease; from risk genes to pathological effects. *Front. Aging Neurosci.*10.3389/fnagi.2021.690372 (2021). 10.3389/fnagi.2021.690372 PMC8264368 34248607

[198] Gamba P (2019). A crosstalk between brain cholesterol oxidation and glucose metabolism in Alzheimer’s disease. Front Neurosci..

[199] Chaudhuri KR, Healy DG, Schapira AHV (2006). Non-motor symptoms of Parkinson’s disease: diagnosis and management. Lancet Neurol..

[200] Schapira AHV, Chaudhuri KR, Jenner P (2017). Non-motor features of Parkinson disease. Nat. Rev. Neurosci..

[201] Damier P, Hirsch EC, Agid Y, Graybiel AM (1999). The substantia nigra of the human brain. II. Patterns of loss of dopamine-containing neurons in Parkinson’s disease. Brain.

[202] Cheng HC, Ulane CM, Burke RE (2010). Clinical progression in Parkinson disease and the neurobiology of axons. Ann. Neurol..

[203] Wirdefeldt K, Adami H-O, Cole P, Trichopoulos D, Mandel J (2011). Epidemiology and etiology of Parkinson’s disease: a review of the evidence. Eur. J. Epidemiol..

[204] Blauwendraat C, Nalls MA, Singleton AB (2020). The genetic architecture of Parkinson’s disease. Lancet Neurol..

[205] Funayama M, Nishioka K, Li Y, Hattori N (2023). Molecular genetics of Parkinson’s disease: contributions and global trends. J. Hum. Genet..

[206] Fung H-C (2006). Genome-wide genotyping in Parkinson’s disease and neurologically normal controls: first stage analysis and public release of data. Lancet Neurol..

[207] Simón-Sánchez J (2009). Genome-wide association study reveals genetic risk underlying Parkinson’s disease. Nat. Genet..

[208] Chartier-Harlin M-C (2004). α-synuclein locus duplication as a cause of familial Parkinson’s disease. Lancet.

[209] Pollanen MS, Dickson DW, Bergeron C (1993). Pathology and biology of the Lewy body. J. Neuropathol. Exp. Neurol..

[210] Gibb WR, Lees AJ (1988). The relevance of the Lewy body to the pathogenesis of idiopathic Parkinson’s disease. J. Neurol. Neurosurg. Psychiatry.

[211] Spillantini MG (1997). α-Synuclein in Lewy bodies. Nature.

[212] McCann H, Stevens CH, Cartwright H, Halliday GM (2014). α-Synucleinopathy phenotypes. Parkinsonism Relat. Disord..

[213] Sulzer D, Edwards RH (2019). The physiological role of α-synuclein and its relationship to Parkinson’s Disease. J. Neurochem..

[214] Chandra S, Gallardo G, Fernández-Chacón R, Schlüter OM, Südhof TC (2005). α-Synuclein cooperates with CSPα in preventing neurodegeneration. Cell.

[215] Bendor, J., Logan, T. & Edwards, R. H. The dunction of α-synuclein. *Neuron* **79**, 1044–1066 (2013). 10.1016/j.neuron.2013.09.004 PMC3866954 24050397

[216] Bartels T (2010). The N-terminus of the intrinsically disordered protein α-synuclein triggers membrane binding and helix folding. Biophys. J..

[217] Weinreb PH, Zhen W, Poon AW, Conway KA, Lansbury PT (1996). NACP, a protein implicated in Alzheimer’s disease and learning, is natively unfolded. Biochemistry.

[218] Chartier S, Duyckaerts C (2018). Is Lewy pathology in the human nervous system chiefly an indicator of neuronal protection or of toxicity?. Cell Tissue Res..

[219] Bengoa-Vergniory N, Roberts RF, Wade-Martins R, Alegre-Abarrategui J (2017). Alpha-synuclein oligomers: a new hope. Acta Neuropathol..

[220] Danzer KM (2012). Exosomal cell-to-cell transmission of alpha synuclein oligomers. Mol. Neurodegener..

[221] Ingelsson, M. Alpha-synuclein oligomers—neurotoxic molecules in Parkinson’s disease and other Lewy body disorders. *Front. Neurosci.*10.3389/fnins.2016.00408 (2016). 10.3389/fnins.2016.00408 PMC5011129 27656123

[222] Winner B (2011). In vivo demonstration that α-synuclein oligomers are toxic. Proc. Natl Acad. Sci. USA.

[223] Wang W (2011). A soluble α-synuclein construct forms a dynamic tetramer. Proc. Natl Acad. Sci. USA.

[224] Bartels T, Choi JG, Selkoe DJ (2011). α-Synuclein occurs physiologically as a helically folded tetramer that resists aggregation. Nature.

[225] Narkiewicz J, Giachin G, Legname G (2014). In vitro aggregation assays for the characterization of α-synuclein prion-like properties. Prion.

[226] Yagi H, Kusaka E, Hongo K, Mizobata T, Kawata Y (2005). Amyloid fibril formation of alpha-synuclein is accelerated by preformed amyloid seeds of other proteins: implications for the mechanism of transmissible conformational diseases. J. Biol. Chem..

[227] Bousset L (2013). Structural and functional characterization of two alpha-synuclein strains. Nat. Commun..

[228] Luk KC (2012). Intracerebral inoculation of pathological α-synuclein initiates a rapidly progressive neurodegenerative α-synucleinopathy in mice. J. Exp. Med..

[229] Mougenot A-L (2012). Prion-like acceleration of a synucleinopathy in a transgenic mouse model. Neurobiol. Aging.

[230] Tanudjojo B (2021). Phenotypic manifestation of α-synuclein strains derived from Parkinson’s disease and multiple system atrophy in human dopaminergic neurons. Nat. Commun..

[231] Goedert M, Spillantini MG, Del Tredici K, Braak H (2013). 100 years of Lewy pathology. Nat. Rev. Neurol..

[232] Visanji NP, Brooks PL, Hazrati L-N, Lang AE (2013). The prion hypothesis in Parkinson’s disease: Braak to the future. Acta Neuropathol. Commun..

[233] Polymeropoulos MH (1996). Mapping of a gene for Parkinson’s disease to chromosome 4q21-q23. Science.

[234] Fuchs J (2007). Phenotypic variation in a large Swedish pedigree due to SNCA duplication and triplication. Neurology.

[235] Zarranz JJ (2004). The new mutation, E46K, of α-synuclein causes parkinson and Lewy body dementia. Ann. Neurol..

[236] Appel-Cresswell S (2013). Alpha-synuclein p.H50Q, a novel pathogenic mutation for Parkinson’s disease. Mov. Disord..

[237] Polymeropoulos MH (1997). Mutation in the alpha-synuclein gene identified in families with Parkinson’s disease. Science.

[238] Ruggeri FS (2020). The influence of pathogenic mutations in α-synuclein on biophysical and structural characteristics of amyloid fibrils. ACS Nano.

[239] Glickman MH, Ciechanover A (2002). The ubiquitin-proteasome proteolytic pathway: destruction for the sake of construction. Physiol. Rev..

[240] Ciechanover A (2005). Proteolysis: from the lysosome to ubiquitin and the proteasome. Nat. Rev. Mol. Cell Biol..

[241] Hipp MS, Park S-H, Hartl FU (2014). Proteostasis impairment in protein-misfolding and -aggregation diseases. Trends Cell Biol..

[242] Hipp MS, Kasturi P, Hartl FU (2019). The proteostasis network and its decline in ageing. Nat. Rev. Mol. Cell Biol..

[243] Kuzuhara S, Mori H, Izumiyama N, Yoshimura M, Ihara Y (1988). Lewy bodies are ubiquitinated. Acta Neuropathol..

[244] Ii K, Ito H, Tanaka K, Hirano A (1997). Immunocytochemical co-localization of the proteasome in ubiquitinated structures in neurodegenerative diseases and the elderly. J. Neuropathol. Exp. Neurol..

[245] Bedford L (2008). Depletion of 26S proteasomes in mouse brain neurons causes neurodegeneration and Lewy-like inclusions resembling human pale bodies. J. Neurosci..

[246] Sun F (2006). Proteasome inhibitor MG-132 induces dopaminergic degeneration in cell culture and animal models. NeuroToxicology.

[247] Schapira AHV (2006). Proteasomal inhibition causes loss of nigral tyrosine hydroxylase neurons. Ann. Neurol..

[248] McKinnon C (2020). Early-onset impairment of the ubiquitin-proteasome system in dopaminergic neurons caused by α-synuclein. Acta Neuropathol. Commun..

[249] Bellomo G, Paciotti S, Gatticchi L, Parnetti L (2020). The vicious cycle between α-synuclein aggregation and autophagic-lysosomal dysfunction. Mov. Disord..

[250] Usenovic M, Tresse E, Mazzulli JR, Taylor JP, Krainc D (2012). Deficiency of ATP13A2 leads to lysosomal dysfunction, α-synuclein accumulation, and neurotoxicity. J. Neurosci..

[251] Murphy KE (2015). Lysosomal-associated membrane protein 2 isoforms are differentially affected in early Parkinson’s disease. Mov. Disord..

[252] Martinez-Vicente M (2008). Dopamine-modified α-synuclein blocks chaperone-mediated autophagy. J. Clin. Investig..

[253] Mazzulli JR, Zunke F, Isacson O, Studer L, Krainc D (2016). α-Synuclein–induced lysosomal dysfunction occurs through disruptions in protein trafficking in human midbrain synucleinopathy models. Proc. Natl Acad. Sci. USA.

[254] Cramb KML, Beccano-Kelly D, Cragg SJ, Wade-Martins R (2023). Impaired dopamine release in Parkinson’s disease. Brain.

[255] Rinne JO (1995). Increased density of dopamine D2 receptors in the putamen, but not in the caudate nucleus in early Parkinson’s disease: a PET study with [11C]raclopride. J. Neurol. Sci..

[256] Block ML, Zecca L, Hong JS (2007). Microglia-mediated neurotoxicity: uncovering the molecular mechanisms. Nat. Rev. Neurosci..

[257] Wang J, Wang F, Mai D, Qu S (2020). Molecular mechanisms of glutamate toxicity in Parkinson’s disease. Front. Neurosci..

[258] Borta A, Höglinger GU (2007). Dopamine and adult neurogenesis. J. Neurochem..

[259] Kamath T (2022). Single-cell genomic profiling of human dopamine neurons identifies a population that selectively degenerates in Parkinson’s disease. Nat. Neurosci..

[260] Lin MT, Beal MF (2006). Mitochondrial dysfunction and oxidative stress in neurodegenerative diseases. Nature.

[261] Langston JW, Ballard P, Tetrud JW, Irwin I (1983). Chronic Parkinsonism in humans due to a product of meperidine-analog synthesis. Science.

[262] Langston JW, Irwin I, Langston EB, Forno LS (1984). 1-methyl-4-phenylpyridinium ion (MPP+): identification of a metabolite of MPTP, a toxin selective to the substantia nigra. Neurosci. Lett..

[263] Watanabe Y, Himeda T, Araki T (2005). Mechanisms of MPTP toxicity and their implications for therapy of Parkinson’s disease. Med. Sci. Monit..

[264] Abou-Sleiman PM, Muqit MMK, Wood NW (2006). Expanding insights of mitochondrial dysfunction in Parkinson’s disease. Nat. Rev. Neurosci..

[265] Exner N, Lutz AK, Haass C, Winklhofer KF (2012). Mitochondrial dysfunction in Parkinson’s disease: molecular mechanisms and pathophysiological consequences. EMBO J..

[266] Dauer W (2002). Resistance of α-synuclein null mice to the parkinsonian neurotoxin MPTP. Proc. Natl Acad. Sci. USA.

[267] Thomas B (2011). Resistance to MPTP-neurotoxicity in α-synuclein knockout mice is complemented by human α-synuclein and associated with increased β-synuclein and Akt activation. PLoS ONE.

[268] Mullin S, Schapira A (2013). α-synuclein and mitochondrial dysfunction in Parkinson’s disease. Mol. Neurobiol..

[269] Grassi D (2018). Identification of a highly neurotoxic α-synuclein species inducing mitochondrial damage and mitophagy in Parkinson’s disease. Proc. Natl Acad. Sci. USA.

[270] Zambon F (2019). Cellular α-synuclein pathology is associated with bioenergetic dysfunction in Parkinson’s iPSC-derived dopamine neurons. Hum. Mol. Genet..

[271] Ryan BJ, Hoek S, Fon EA, Wade-Martins R (2015). Mitochondrial dysfunction and mitophagy in Parkinson’s: from familial to sporadic disease. Trends Biochem. Sci..

[272] Larsen SB, Hanss Z, Krüger R (2018). The genetic architecture of mitochondrial dysfunction in Parkinson’s disease. Cell Tissue Res..

[273] Pickrell AliciaM, Youle RichardJ (2015). The roles of PINK1, Parkin, and mitochondrial fidelity in Parkinson’s disease. Neuron.

[274] Ge P, Dawson VL, Dawson TM (2020). PINK1 and Parkin mitochondrial quality control: a source of regional vulnerability in Parkinson’s disease. Mol. Neurodegener..

[275] Geisler S (2010). PINK1/Parkin-mediated mitophagy is dependent on VDAC1 and p62/SQSTM1. Nat. Cell Biol..

[276] Quinn PMJ, Moreira PI, Ambrósio AF, Alves CH (2020). PINK1/PARKIN signalling in neurodegeneration and neuroinflammation. Acta Neuropathol. Commun..

[277] Bonifati V (2003). Mutations in the DJ-1 gene associated with autosomal recessive early-onset parkinsonism. Science.

[278] Dolgacheva LP, Berezhnov AV, Fedotova EI, Zinchenko VP, Abramov AY (2019). Role of DJ-1 in the mechanism of pathogenesis of Parkinson’s disease. J. Bioenerg. Biomembr..

[279] Guzman JN (2010). Oxidant stress evoked by pacemaking in dopaminergic neurons is attenuated by DJ-1. Nature.

[280] Zhang L (2005). Mitochondrial localization of the Parkinson’s disease related protein DJ-1: implications for pathogenesis. Hum. Mol. Genet..

[281] Taira T (2004). DJ-1 has a role in antioxidative stress to prevent cell death. EMBO Rep..

[282] Canet-Avilés RM (2004). The Parkinson’s disease protein DJ-1 is neuroprotective due to cysteine-sulfinic acid-driven mitochondrial localization. Proc. Natl Acad. Sci. USA.

[283] Nabi M, Tabassum N (2022). Role of environmental toxicants on neurodegenerative disorders. Front. Toxicol..

[284] Ramirez Ortega D (2020). Kynurenine pathway as a new target of cognitive impairment induced by lead toxicity during the lactation. Sci. Rep..

[285] Cannon JR, Greenamyre JT (2011). The role of environmental exposures in neurodegeneration and neurodegenerative diseases. Toxicol. Sci..

[286] Liu Z (2023). Anionic nanoplastic contaminants promote Parkinson’s disease–associated α-synuclein aggregation. Sci. Adv..

[287] Mejzini R (2019). ALS genetics, mechanisms, and therapeutics: where are we now?. Front. Neurosci..

[288] Suzuki, N., Nishiyama, A., Warita, H. & Aoki, M. Genetics of amyotrophic lateral sclerosis: seeking therapeutic targets in the era of gene therapy. *J. Hum. Genet.*10.1038/s10038-022-01055-8 (2022). 10.1038/s10038-022-01055-8 PMC9968660 35691950

[289] Smukowski SN (2022). Progress in amyotrophic lateral sclerosis gene discovery: reflecting on classic approaches and leveraging emerging technologies. Neurol Genet..

[290] Talbott EO, Malek AM, Lacomis D (2016). The epidemiology of amyotrophic lateral sclerosis. Handb. Clin. Neurol..

[291] Saberi S, Stauffer JE, Schulte DJ, Ravits J (2015). Neuropathology of amyotrophic lateral sclerosis and its variants. Neurol. Clin..

[292] Goldstein LH, Abrahams S (2013). Changes in cognition and behaviour in amyotrophic lateral sclerosis: nature of impairment and implications for assessment. Lancet Neurol..

[293] Sanes JR, Lichtman JW (2001). Induction, assembly, maturation and maintenance of a postsynaptic apparatus. Nat. Rev. Neurosci..

[294] Chowdhury A, Mukherjee A, Sinharoy U, Pandit A, Biswas A (2021). Non-motor features of amyotrophic lateral sclerosis: a clinic-based study. Ann. Indian Acad. Neurol..

[295] Blokhuis AM, Groen EJN, Koppers M, van den Berg LH, Pasterkamp RJ (2013). Protein aggregation in amyotrophic lateral sclerosis. Acta Neuropathol..

[296] Hayashi Y, Homma K, Ichijo H (2016). SOD1 in neurotoxicity and its controversial roles in SOD1 mutation-negative ALS. Adv. Biol. Regul..

[297] Forsberg K (2010). Novel antibodies reveal inclusions containing non-native SOD1 in sporadic ALS patients. PLoS ONE.

[298] Paré B (2018). Misfolded SOD1 pathology in sporadic amyotrophic lateral sclerosis. Sci. Rep..

[299] Berdyński M (2022). SOD1 mutations associated with amyotrophic lateral sclerosis analysis of variant severity. Sci. Rep..

[300] Benkler C (2018). Aggregated SOD1 causes selective death of cultured human motor neurons. Sci. Rep..

[301] Forsberg K, Andersen PM, Marklund SL, Brännström T (2011). Glial nuclear aggregates of superoxide dismutase-1 are regularly present in patients with amyotrophic lateral sclerosis. Acta Neuropathol..

[302] Ayers JI, Fromholt SE, O’Neal VM, Diamond JH, Borchelt DR (2016). Prion-like propagation of mutant SOD1 misfolding and motor neuron disease spread along neuroanatomical pathways. Acta Neuropathol..

[303] Grad LI (2014). Intercellular propagated misfolding of wild-type Cu/Zn superoxide dismutase occurs via exosome-dependent and -independent mechanisms. Proc. Natl Acad. Sci. USA.

[304] Münch C, O’Brien J, Bertolotti A (2011). Prion-like propagation of mutant superoxide dismutase-1 misfolding in neuronal cells. Proc. Natl Acad. Sci. USA.

[305] Thomas EV, Fenton WA, McGrath J, Horwich AL (2017). Transfer of pathogenic and nonpathogenic cytosolic proteins between spinal cord motor neurons in vivo in chimeric mice. Proc. Natl Acad. Sci. USA.

[306] Da Cruz S (2017). Misfolded SOD1 is not a primary component of sporadic ALS. Acta Neuropathol..

[307] Neumann M (2006). Ubiquitinated TDP-43 in frontotemporal lobar degeneration and amyotrophic lateral sclerosis. Science.

[308] Xu Z, Yang C (2014). TDP-43-the key to understanding amyotrophic lateral sclerosis. Rare Dis..

[309] Arseni D (2022). Structure of pathological TDP-43 filaments from ALS with FTLD. Nature.

[310] Lipstein N (2021). Munc13-1 is a Ca2+-phospholipid-dependent vesicle priming hub that shapes synaptic short-term plasticity and enables sustained neurotransmission. Neuron.

[311] Brown A-L (2022). TDP-43 loss and ALS-risk SNPs drive mis-splicing and depletion of UNC13A. Nature.

[312] Ma XR (2022). TDP-43 represses cryptic exon inclusion in the FTD–ALS gene UNC13A. Nature.

[313] Vogler TO (2018). TDP-43 and RNA form amyloid-like myo-granules in regenerating muscle. Nature.

[314] Keller BA (2012). Co-aggregation of RNA binding proteins in ALS spinal motor neurons: evidence of a common pathogenic mechanism. Acta Neuropathol..

[315] Choi SY (2019). C9ORF72-ALS/FTD-associated poly(GR) binds Atp5a1 and compromises mitochondrial function in vivo. Nat. Neurosci..

[316] May S (2014). C9orf72 FTLD/ALS-associated Gly-Ala dipeptide repeat proteins cause neuronal toxicity and Unc119 sequestration. Acta Neuropathol..

[317] Mizielinska S (2014). C9orf72 repeat expansions cause neurodegeneration in drosophila through arginine-rich proteins. Science.

[318] Ryan S, Rollinson S, Hobbs E, Pickering-Brown S (2022). C9orf72 dipeptides disrupt the nucleocytoplasmic transport machinery and cause TDP-43 mislocalisation to the cytoplasm. Sci. Rep..

[319] Tran H (2022). Suppression of mutant C9orf72 expression by a potent mixed backbone antisense oligonucleotide. Nat. Med..

[320] DeJesus-Hernandez M (2011). Expanded GGGGCC hexanucleotide repeat in noncoding region of C9ORF72 causes chromosome 9p-linked FTD and ALS. Neuron.

[321] Liu, J. & Wang, F. Role of neuroinflammation in amyotrophic lateral sclerosis: cellular mechanisms and therapeutic implications. *Front. Immunol.*10.3389/fimmu.2017.01005 (2017). 10.3389/fimmu.2017.01005 PMC5567007 28871262

[322] Liu, E., Karpf, L. & Bohl, D. Neuroinflammation in amyotrophic lateral sclerosis and frontotemporal dementia and the interest of induced pluripotent stem cells to study immune cells interactions with neurons. *Front. Mol. Neurosci.*10.3389/fnmol.2021.767041 (2021). 10.3389/fnmol.2021.767041 PMC8712677 34970118

[323] Liao B, Zhao W, Beers DR, Henkel JS, Appel SH (2012). Transformation from a neuroprotective to a neurotoxic microglial phenotype in a mouse model of ALS. Exp. Neurol..

[324] Nagai M (2007). Astrocytes expressing ALS-linked mutated SOD1 release factors selectively toxic to motor neurons. Nat. Neurosci..

[325] Howland DS (2002). Focal loss of the glutamate transporter EAAT2 in a transgenic rat model of SOD1 mutant-mediated amyotrophic lateral sclerosis (ALS). Proc. Natl Acad. Sci. USA.

[326] Ferraiuolo L (2011). Dysregulation of astrocyte–motoneuron cross-talk in mutant superoxide dismutase 1-related amyotrophic lateral sclerosis. Brain.

[327] Hensley K (2006). Primary glia expressing the G93A-SOD1 mutation present a neuroinflammatory phenotype and provide a cellular system for studies of glial inflammation. J. Neuroinflamm..

[328] Marchetto MC (2008). Non-cell-autonomous effect of human SOD1G37R astrocytes on motor neurons derived from human embryonic stem cells. Cell Stem Cell.

[329] Re DianeB (2014). Necroptosis drives motor neuron death in models of both sporadic and familial ALS. Neuron.

[330] Ferraiuolo L (2016). Oligodendrocytes contribute to motor neuron death in ALS via SOD1-dependent mechanism. Proc. Natl Acad. Sci. USA.

[331] Nakaya T, Maragkakis M (2018). Amyotrophic lateral sclerosis associated FUS mutation shortens mitochondria and induces neurotoxicity. Sci. Rep..

[332] Magrané J, Cortez C, Gan W-B, Manfredi G (2013). Abnormal mitochondrial transport and morphology are common pathological denominators in SOD1 and TDP43 ALS mouse models. Hum. Mol. Genet..

[333] Lopez-Gonzalez R (2016). Poly(GR) in C9ORF72-related ALS/FTD compromises mitochondrial function and increases oxidative stress and DNA damage in iPSC-derived motor neurons. Neuron.

[334] Singh T (2021). Neuronal mitochondrial dysfunction in sporadic amyotrophic lateral sclerosis is developmentally regulated. Sci. Rep..

[335] Butti, Z. & Patten, S. A. RNA Dysregulation in amyotrophic lateral sclerosis. *Front. Genet.*10.3389/fgene.2018.00712 (2019). 10.3389/fgene.2018.00712 PMC6349704 30723494

[336] Buratti, E. & Baralle, F. E. Multiple roles of TDP-43 in gene expression, splicing regulation, and human disease. *Front. Biosci.* **13**, 867–878 (2008). 10.2741/2727 17981595

[337] Polymenidou M (2011). Long pre-mRNA depletion and RNA missplicing contribute to neuronal vulnerability from loss of TDP-43. Nat. Neurosci..

[338] Ross CA (2014). Huntington disease: natural history, biomarkers and prospects for therapeutics. Nat. Rev. Neurol..

[339] Johnson EB (2021). Dynamics of cortical degeneration over a decade in Huntington’s disease. Biol. Psychiatry.

[340] Rosas HD (2008). Cerebral cortex and the clinical expression of Huntington’s disease: complexity and heterogeneity. Brain.

[341] A novel gene containing a trinucleotide repeat that is expanded and unstable on Huntington’s disease chromosomes. The Huntington’s Disease Collaborative Research Group. *Cell* **72**, 971–983 (1993). 10.1016/0092-8674(93)90585-e 8458085

[342] Nasir J (1995). Targeted disruption of the Huntington’s disease gene results in embryonic lethality and behavioral and morphological changes in heterozygotes. Cell.

[343] Zuccato C, Cattaneo E (2014). Huntington’s disease. Handb. Exp. Pharmacol..

[344] Kennedy L (2003). Dramatic tissue-specific mutation length increases are an early molecular event in Huntington disease pathogenesis. Hum. Mol. Genet..

[345] Mangiarini L (1997). Instability of highly expanded CAG repeats in mice transgenic for the Huntington’s disease mutation. Nat. Genet..

[346] Rosenblatt A (2012). Age, CAG repeat length, and clinical progression in Huntington’s disease. Mov. Disord..

[347] Aviolat H (2019). Assessing average somatic CAG repeat instability at the protein level. Sci. Rep..

[348] Kim M (2013). Beta conformation of polyglutamine track revealed by a crystal structure of Huntingtin N-terminal region with insertion of three histidine residues. Prion.

[349] Yamamoto A, Lucas JJ, Hen R (2000). Reversal of neuropathology and motor dysfunction in a conditional model of Huntington’s disease. Cell.

[350] Harper SQ (2005). RNA interference improves motor and neuropathological abnormalities in a Huntington’s disease mouse model. Proc. Natl Acad. Sci. USA.

[351] Sathasivam K (2013). Aberrant splicing of HTT generates the pathogenic exon 1 protein in Huntington disease. Proc. Natl Acad. Sci. USA.

[352] Benn CL (2005). Contribution of nuclear and extranuclear polyQ to neurological phenotypes in mouse models of Huntington’s disease. Hum. Mol. Genet..

[353] Saudou F, Finkbeiner S, Devys D, Greenberg ME (1998). Huntingtin acts in the nucleus to induce apoptosis but death does not correlate with the formation of intranuclear inclusions. Cell.

[354] Neueder A (2017). The pathogenic exon 1 HTT protein is produced by incomplete splicing in Huntington’s disease patients. Sci. Rep..

[355] Hoffner G, Island ML, Djian P (2005). Purification of neuronal inclusions of patients with Huntington’s disease reveals a broad range of N-terminal fragments of expanded huntingtin and insoluble polymers. J. Neurochem..

[356] Yang H (2020). Truncation of mutant huntingtin in knock-in mice demonstrates exon1 huntingtin is a key pathogenic form. Nat. Commun..

[357] Arrasate M, Mitra S, Schweitzer ES, Segal MR, Finkbeiner S (2004). Inclusion body formation reduces levels of mutant huntingtin and the risk of neuronal death. Nature.

[358] Miller J (2011). Identifying polyglutamine protein species in situ that best predict neurodegeneration. Nat. Chem. Biol..

[359] Nucifora LG (2012). Identification of novel potentially toxic oligomers formed in vitro from mammalian-derived expanded huntingtin exon-1 protein. J. Biol. Chem..

[360] DiGiovanni LF, Mocle AJ, Xia J, Truant R (2016). Huntingtin N17 domain is a reactive oxygen species sensor regulating Huntingtin phosphorylation and localization. Hum. Mol. Genet..

[361] Moldovean SN, Chiş V (2020). Molecular dynamics simulations applied to structural and dynamical transitions of the Huntingtin protein: a review. ACS Chem. Neurosci..

[362] Owada R, Mitsui S, Nakamura K (2022). Exogenous polyserine and polyleucine are toxic to recipient cells. Sci. Rep..

[363] Ramdzan YM (2017). Huntingtin inclusions trigger cellular quiescence, deactivate apoptosis, and lead to delayed necrosis. Cell Rep..

[364] Layburn FE (2022). N-terminal mutant huntingtin deposition correlates with CAG repeat length and symptom onset, but not neuronal loss in Huntington’s disease. Neurobiol. Dis..

[365] Costanzo M (2013). Transfer of polyglutamine aggregates in neuronal cells occurs in tunneling nanotubes. J. Cell Sci..

[366] Babcock DT, Ganetzky B (2015). Transcellular spreading of huntingtin aggregates in the drosophila brain. Proc. Natl Acad. Sci. USA.

[367] Pearce MMP, Spartz EJ, Hong W, Luo L, Kopito RR (2015). Prion-like transmission of neuronal huntingtin aggregates to phagocytic glia in the Drosophila brain. Nat. Commun..

[368] Lin JT (2013). Regulation of feedback between protein kinase A and the proteasome system worsens Huntington’s disease. Mol. Cell. Biol..

[369] Ravikumar B (2004). Inhibition of mTOR induces autophagy and reduces toxicity of polyglutamine expansions in fly and mouse models of Huntington disease. Nat. Genet..

[370] Ly S (2022). Mutant huntingtin messenger RNA forms neuronal nuclear clusters in rodent and human brains. Brain Commun..

[371] Gratuze M, Cisbani G, Cicchetti F, Planel E (2016). Is Huntington’s disease a tauopathy?. Brain.

[372] Vuono R (2015). The role of tau in the pathological process and clinical expression of Huntington’s disease. Brain.

[373] Fernández-Nogales M (2014). Huntington’s disease is a four-repeat tauopathy with tau nuclear rods. Nat. Med..

[374] Blum D (2015). Mutant huntingtin alters Tau phosphorylation and subcellular distribution. Hum. Mol. Genet..

[375] Gratuze M (2015). Tau hyperphosphorylation and deregulation of calcineurin in mouse models of Huntington’s disease. Hum. Mol. Genet..

[376] Lahue RS (2020). New developments in Huntington’s disease and other triplet repeat diseases: DNA repair turns to the dark side. Neuronal Signal.

[377] Lee J-M (2019). CAG repeat not polyglutamine length determines timing of Huntington’s disease onset. Cell.

[378] Bunting E (2022). I01|Msh3-targeting antisense oligonucleotides halt CAG repeat expansions in Huntington’s disease IPSC-derived neurons. J. Neurol. Neurosurg. Psychiatry.

[379] McAllister B (2022). Exome sequencing of individuals with Huntington’s disease implicates FAN1 nuclease activity in slowing CAG expansion and disease onset. Nat. Neurosci..

[380] Maiuri T (2016). Huntingtin is a scaffolding protein in the ATM oxidative DNA damage response complex. Hum. Mol. Genet..

[381] Hodges A (2006). Regional and cellular gene expression changes in human Huntington’s disease brain. Hum. Mol. Genet..

[382] Guo S (2022). MicroRNA editing patterns in Huntington’s disease. Sci. Rep..

[383] Hervás-Corpión I (2018). Early alteration of epigenetic-related transcription in Huntington’s disease mouse models. Sci. Rep..

[384] Mouro Pinto R (2020). Patterns of CAG repeat instability in the central nervous system and periphery in Huntington’s disease and in spinocerebellar ataxia type 1. Hum. Mol. Genet..

[385] Vonsattel JP (1985). Neuropathological classification of Huntington’s disease. J. Neuropathol. Exp. Neurol..

[386] Graveland GA, Williams RS, DiFiglia M (1985). Evidence for degenerative and regenerative changes in neostriatal spiny neurons in Huntington’s disease. Science.

[387] Victor MB (2018). Striatal neurons directly converted from Huntington’s disease patient fibroblasts recapitulate age-associated disease phenotypes. Nat. Neurosci..

[388] Cicchetti F, Soulet D, Freeman TB (2011). Neuronal degeneration in striatal transplants and Huntington’s disease: potential mechanisms and clinical implications. Brain.

[389] Olney JW, Gubareff TD (1978). Glutamate neurotoxicity and Huntington’s chorea. Nature.

[390] Skotte NH (2018). Integrative characterization of the R6/2 mouse model of Huntington’s disease reveals dysfunctional astrocyte metabolism. Cell Rep..

[391] Braz BY (2022). Treating early postnatal circuit defect delays Huntington’s disease onset and pathology in mice. Science.

[392] Kim J (2010). Mitochondrial loss, dysfunction and altered dynamics in Huntington’s disease. Hum. Mol. Genet..

[393] Johri A, Chandra A, Flint Beal M (2013). PGC-1α, mitochondrial dysfunction, and Huntington’s disease. Free Radic. Biol. Med..

[394] Browne SE, Beal MF (2004). The energetics of Huntington’s disease. Neurochem. Res..

[395] Cui L (2006). Transcriptional repression of PGC-1alpha by mutant huntingtin leads to mitochondrial dysfunction and neurodegeneration. Cell.

[396] Panov AV (2002). Early mitochondrial calcium defects in Huntington’s disease are a direct effect of polyglutamines. Nat. Neurosci..

[397] Manczak M, Reddy PH (2015). Mitochondrial division inhibitor 1 protects against mutant huntingtin-induced abnormal mitochondrial dynamics and neuronal damage in Huntington’s disease. Hum. Mol. Genet..

[398] Haun F (2013). S-nitrosylation of dynamin-related protein 1 mediates mutant huntingtin-induced mitochondrial fragmentation and neuronal injury in Huntington’s disease. Antioxid. Redox Signal..

[399] Altar CA (1997). Anterograde transport of brain-derived neurotrophic factor and its role in the brain. Nature.

[400] Gauthier LR (2004). Huntingtin controls neurotrophic support and survival of neurons by enhancing BDNF vesicular transport along microtubules. Cell.

[401] Zuccato C (2001). Loss of Huntingtin-mediated BDNF gene transcription in Huntington’s disease. Science.

[402] Hong Y, Zhao T, Li XJ, Li S (2016). Mutant Huntingtin impairs BDNF release from astrocytes by disrupting conversion of Rab3a-GTP into Rab3a-GDP. J. Neurosci..

[403] Rocha, N. P. et al. Microglia activation in basal ganglia is a late event in Huntington disease pathophysiology. *Neurol. Neuroimmunol. Neuroinflamm.*10.1212/nxi.0000000000000984 (2021). 10.1212/NXI.0000000000000984 PMC8017723 33795375

[404] Khakh BS (2017). Unravelling and exploiting astrocyte dysfunction in Huntington’s disease. Trends Neurosci..

[405] Lee H-G, Wheeler MA, Quintana FJ (2022). Function and therapeutic value of astrocytes in neurological diseases. Nat. Rev. Drug Discov..

[406] Benraiss A (2016). Human glia can both induce and rescue aspects of disease phenotype in Huntington disease. Nat. Commun..

[407] Liddelow SA (2017). Neurotoxic reactive astrocytes are induced by activated microglia. Nature.

[408] Faideau M (2010). In vivo expression of polyglutamine-expanded huntingtin by mouse striatal astrocytes impairs glutamate transport: a correlation with Huntington’s disease subjects. Hum. Mol. Genet..

[409] Shin J-Y (2005). Expression of mutant Huntingtin in glial cells contributes to neuronal excitotoxicity. J. Cell Biol..

[410] O’Regan GC (2021). Human Huntington’s disease pluripotent stem cell-derived microglia develop normally but are abnormally hyper-reactive and release elevated levels of reactive oxygen species. J. Neuroinflamm..

[411] Tan H-Y, Cho H, Lee LP (2021). Human mini-brain models. Nat. Biomed. Eng..

[412] Fang, G., Chen, Y.-C., Lu, H. & Jin, D. Advances in spheroids and organoids on a chip. *Adv. Funct. Mater.* **33**, 2215043 (2023).

[413] Huh D (2010). Reconstituting organ-level lung functions on a chip. Science.

[414] Huh D (2012). A human disease model of drug toxicity-induced pulmonary edema in a lung-on-a-chip microdevice. Sci. Transl. Med..

[415] Kim HJ, Huh D, Hamilton G, Ingber DE (2012). Human gut-on-a-chip inhabited by microbial flora that experiences intestinal peristalsis-like motions and flow. Lab Chip.

[416] Musah, S. et al. Mature induced-pluripotent-stem-cell-derived human podocytes reconstitute kidney glomerular-capillary-wall function on a chip. *Nat Biomed. Eng.*10.1038/s41551-017-0069 (2017). 10.1038/s41551-017-0069 PMC5639718 29038743

[417] Azevedo FA (2009). Equal numbers of neuronal and nonneuronal cells make the human brain an isometrically scaled-up primate brain. J. Comp. Neurol..

[418] Piwecka M, Rajewsky N, Rybak-Wolf A (2023). Single-cell and spatial transcriptomics: deciphering brain complexity in health and disease. Nat. Rev. Neurol..

[419] Yokoyama, M., Kobayashi, H., Tatsumi, L. & Tomita, T. Mouse models of Alzheimer’s disease. *Front. Mol. Neurosci.*10.3389/fnmol.2022.912995 (2022). 10.3389/fnmol.2022.912995 PMC9254908 35799899

[420] Dovonou A (2023). Animal models of Parkinson’s disease: bridging the gap between disease hallmarks and research questions. Transl. Neurodegener..

[421] Fisher EMC (2023). Opinion: more mouse models and more translation needed for ALS. Mol. Neurodegener..

[422] Pouladi MA, Morton AJ, Hayden MR (2013). Choosing an animal model for the study of Huntington’s disease. Nat. Rev. Neurosci..

[423] Uhl EW, Warner NJ (2015). Mouse models as predictors of human responses: evolutionary medicine. Curr. Pathobiol. Rep..

[424] Semple BD, Blomgren K, Gimlin K, Ferriero DM, Noble-Haeusslein LJ (2013). Brain development in rodents and humans: Identifying benchmarks of maturation and vulnerability to injury across species. Prog. Neurobiol..

[425] Baglietto-Vargas D (2021). Generation of a humanized Aβ expressing mouse demonstrating aspects of Alzheimer’s disease-like pathology. Nat. Commun..

[426] Balducci C (2010). Synthetic amyloid-β oligomers impair long-term memory independently of cellular prion protein. Proc. Natl Acad. Sci. USA.

[427] Luk KC (2012). Pathological α-synuclein transmission initiates Parkinson-like neurodegeneration in nontransgenic mice. Science.

[428] Chung HK, Ho HA, Pérez-Acuña D, Lee SJ (2019). Modeling α-synuclein propagation with preformed fibril injections. J. Mov. Disord..

[429] Shadrina, M. & Slominsky, P. Modeling Parkinson’s disease: not only rodents? *Front. Aging Neurosci.*10.3389/fnagi.2021.695718 (2021). 10.3389/fnagi.2021.695718 PMC8377290 34421573

[430] Nistor G (2011). Derivation of high purity neuronal progenitors from human embryonic stem cells. PLoS ONE.

[431] Rivetti di Val Cervo P, Besusso D, Conforti P, Cattaneo E (2021). hiPSCs for predictive modelling of neurodegenerative diseases: dreaming the possible. Nat. Rev. Neurol..

[432] Takahashi K, Yamanaka S (2006). Induction of pluripotent stem cells from mouse embryonic and adult fibroblast cultures by defined factors. Cell.

[433] Kang S (2017). Characteristic analyses of a neural differentiation model from iPSC-derived neuron according to morphology, physiology, and global gene expression pattern. Sci. Rep..

[434] Penney J, Ralvenius WT, Tsai L-H (2020). Modeling Alzheimer’s disease with iPSC-derived brain cells. Mol. Psychiatry.

[435] Chen SW (2021). Efficient conversion of human induced pluripotent stem cells into microglia by defined transcription factors. Stem Cell Rep..

[436] Marton RM, Ioannidis JPA (2019). A comprehensive analysis of protocols for deriving dopaminergic neurons from human pluripotent stem cells. Stem Cells Transl. Med..

[437] Wang Y (2018). Generation of induced pluripotent stem cell line (ZZUi007-A) from a 52-year-old patient with a novel CHCHD2 gene mutation in Parkinson’s disease. Stem Cell Res..

[438] Chung SY (2016). Parkin and PINK1 patient iPSC-derived midbrain dopamine neurons exhibit mitochondrial dysfunction and α-synuclein accumulation. Stem Cell Rep..

[439] Jankowsky JL, Zheng H (2017). Practical considerations for choosing a mouse model of Alzheimer’s disease. Mol. Neurodegener..

[440] Campenot RB (1977). Local control of neurite development by nerve growth factor. Proc. Natl Acad. Sci. USA.

[441] Megarity D (2022). A modular microfluidic platform to enable complex and customisable in vitro models for neuroscience. Lab Chip.

[442] Peyrin J-M (2011). Axon diodes for the reconstruction of oriented neuronal networks in microfluidic chambers. Lab Chip.

[443] Renault R, Durand J-B, Viovy J-L, Villard C (2016). Asymmetric axonal edge guidance: a new paradigm for building oriented neuronal networks. Lab Chip.

[444] Courte J (2018). Reconstruction of directed neuronal networks in a microfluidic device with asymmetric microchannels. Methods Cell Biol..

[445] le Feber, J., Postma, W., de Weerd, E., Weusthof, M. & Rutten, W. L. C. Barbed channels enhance unidirectional connectivity between neuronal networks cultured on multi electrode arrays. *Front. Neurosci.*10.3389/fnins.2015.00412 (2015). 10.3389/fnins.2015.00412 PMC4630305 26578869

[446] Gu L (2014). Microfluidic control of axonal guidance. Sci. Rep..

[447] Kim J, Kang M, Jensen EC, Mathies RA (2012). Lifting gate polydimethylsiloxane microvalves and pumps for microfluidic control. Anal. Chem..

[448] Unger MA, Chou HP, Thorsen T, Scherer A, Quake SR (2000). Monolithic microfabricated valves and pumps by multilayer soft lithography. Science.

[449] Sesen M, Rowlands CJ (2021). Thermally-actuated microfluidic membrane valve for point-of-care applications. Microsyst. Nanoeng..

[450] Fernandes JTS (2014). Modulation of alpha-synuclein toxicity in yeast using a novel microfluidic-based gradient generator. Lab Chip.

[451] Choi YJ (2013). Neurotoxic amyloid beta oligomeric assemblies recreated in microfluidic platform with interstitial level of slow flow. Sci. Rep..

[452] Shin Y (2019). Blood–brain barrier dysfunction in a 3D in vitro model of Alzheimer’s disease. Adv. Sci..

[453] Pediaditakis I (2021). Modeling alpha-synuclein pathology in a human brain-chip to assess blood-brain barrier disruption. Nat. Commun..

[454] Leung CM (2022). A guide to the organ-on-a-chip. Nat. Rev. Methods Prim..

[455] Aleman J, Kilic T, Mille LS, Shin SR, Zhang YS (2021). Microfluidic integration of regeneratable electrochemical affinity-based biosensors for continual monitoring of organ-on-a-chip devices. Nat. Protoc..

[456] Henry OYF (2017). Organs-on-chips with integrated electrodes for trans-epithelial electrical resistance (TEER) measurements of human epithelial barrier function. Lab Chip.

[457] Moutaux E, Charlot B, Genoux A, Saudou F, Cazorla M (2018). An integrated microfluidic/microelectrode array for the study of activity-dependent intracellular dynamics in neuronal networks. Lab Chip.

[458] Salman MM (2020). Design and validation of a human brain endothelial microvessel-on-a-chip open microfluidic model enabling advanced optical imaging. Front. Bioeng. Biotechnol..

[459] Centeno EGZ, Cimarosti H, Bithell A (2018). 2D versus 3D human induced pluripotent stem cell-derived cultures for neurodegenerative disease modelling. Mol. Neurodegener..

[460] Hebisch M (2023). The impact of the cellular environment and aging on modeling Alzheimer’s disease in 3D cell culture models. Adv. Sci..

[461] Bolognin S (2019). 3D cultures of Parkinson’s disease-specific dopaminergic neurons for high content phenotyping and drug testing. Adv. Sci..

[462] Choi SH (2014). A three-dimensional human neural cell culture model of Alzheimer’s disease. Nature.

[463] Kwak SS (2020). Amyloid-β42/40 ratio drives tau pathology in 3D human neural cell culture models of Alzheimer’s disease. Nat. Commun..

[464] Baruffaldi D, Palmara G, Pirri C, Frascella F (2021). 3D cell culture: recent development in materials with tunable stiffness. ACS Appl. Bio Mater..

[465] Segel M (2019). Niche stiffness underlies the ageing of central nervous system progenitor cells. Nature.

[466] Murphy MC (2016). Regional brain stiffness changes across the Alzheimer’s disease spectrum. Neuroimage Clin..

[467] Sobrino A (2016). 3D microtumors in vitro supported by perfused vascular networks. Sci. Rep..

[468] Trietsch SJ (2017). Membrane-free culture and real-time barrier integrity assessment of perfused intestinal epithelium tubes. Nat. Commun..

[469] Dujardin S (2014). Neuron-to-neuron wild-type Tau protein transfer through a trans-synaptic mechanism: relevance to sporadic tauopathies. Acta Neuropathol. Commun..

[470] Takeda S (2015). Neuronal uptake and propagation of a rare phosphorylated high-molecular-weight tau derived from Alzheimer’s disease brain. Nat. Commun..

[471] Wu JW (2013). Small misfolded Tau species are internalized via bulk endocytosis and anterogradely and retrogradely transported in neurons. J. Biol. Chem..

[472] Usenovic M (2015). Internalized tau oligomers cause neurodegeneration by inducing accumulation of pathogenic tau in human neurons derived from induced pluripotent stem cells. J. Neurosci..

[473] Kunze A, Meissner R, Brando S, Renaud P (2011). Co-pathological connected primary neurons in a microfluidic device for alzheimer studies. Biotechnol. Bioeng..

[474] Annweiler C, Brugg B, Peyrin J-M, Bartha R, Beauchet O (2014). Combination of memantine and vitamin D prevents axon degeneration induced by amyloid-beta and glutamate. Neurobiol. Aging.

[475] Poon WW (2011). β-Amyloid impairs axonal BDNF retrograde trafficking. Neurobiol. Aging.

[476] Deleglise B (2014). β-amyloid induces a dying-back process and remote trans-synaptic alterations in a microfluidic-based reconstructed neuronal network. Acta Neuropathol. Commun..

[477] Wu JW (2016). Neuronal activity enhances tau propagation and tau pathology in vivo. Nat. Neurosci..

[478] Calafate S (2015). Synaptic contacts enhance cell-to-cell tau pathology propagation. Cell Rep..

[479] Ruiz A (2014). Testing Aβ toxicity on primary CNS cultures using drug-screening microfluidic chips. Lab Chip.

[480] Maragakis NJ, Rothstein JD (2006). Mechanisms of disease: astrocytes in neurodegenerative disease. Nat. Clin. Pract. Neurol..

[481] Liu, J. et al. Microglia: a double-edged sword in intracerebral hemorrhage from basic mechanisms to clinical research. *Front. Immunol.*10.3389/fimmu.2021.675660 (2021). 10.3389/fimmu.2021.675660 PMC8135095 34025674

[482] Park J (2018). A 3D human triculture system modeling neurodegeneration and neuroinflammation in Alzheimer’s disease. Nat. Neurosci..

[483] Baik SH (2014). Migration of neutrophils targeting amyloid plaques in Alzheimer’s disease mouse model. Neurobiol. Aging.

[484] Cho H (2013). Microfluidic chemotaxis platform for differentiating the roles of soluble and bound amyloid-β on microglial accumulation. Sci. Rep..

[485] Park J (2015). Three-dimensional brain-on-a-chip with an interstitial level of flow and its application as an in vitro model of Alzheimer’s disease. Lab Chip.

[486] Soden PA, Henderson AR, Lee E (2022). A microfluidic model of AQP4 polarization dynamics and fluid transport in the healthy and inflamed human brain: the first step towards glymphatics-on-a-chip. Adv. Biol..

[487] Seidi A (2011). A microfluidic-based neurotoxin concentration gradient for the generation of an in vitro model of Parkinson’s disease. Biomicrofluidics.

[488] Brahic M, Bousset L, Bieri G, Melki R, Gitler AD (2016). Axonal transport and secretion of fibrillar forms of α-synuclein, Aβ42 peptide and HTTExon 1. Acta Neuropathol..

[489] Freundt EC (2012). Neuron-to-neuron transmission of α-synuclein fibrils through axonal transport. Ann. Neurol..

[490] Van Laar, V. S. et al. Evidence for compartmentalized axonal mitochondrial biogenesis: mitochondrial DNA replication increases in distal axons as an early response to Parkinson’s disease-relevant stress. *J. Neurosci.* **38**, 7505–7515 (2018). 10.1523/JNEUROSCI.0541-18.2018 PMC6104298 30030401

[491] Braak H, Ghebremedhin E, Rüb U, Bratzke H, Del Tredici K (2004). Stages in the development of Parkinson’s disease-related pathology. Cell Tissue Res..

[492] Prots I (2018). α-Synuclein oligomers induce early axonal dysfunction in human iPSC-based models of synucleinopathies. Proc. Natl Acad. Sci. USA.

[493] Volpicelli-Daley LA, Luk KC, Lee VM (2014). Addition of exogenous α-synuclein preformed fibrils to primary neuronal cultures to seed recruitment of endogenous α-synuclein to Lewy body and Lewy neurite-like aggregates. Nat. Protoc..

[494] Fernandes, J. T. S., Chutna, O., Chu, V., Conde, J. P. & Outeiro, T. F. A novel microfluidic cell co-culture platform for the study of the molecular mechanisms of Parkinson’s disease and other synucleinopathies. *Front. Neurosci.*10.3389/fnins.2016.00511 (2016). 10.3389/fnins.2016.00511 PMC5108800 27895548

[495] Gribaudo S (2019). Propagation of α-synuclein strains within human reconstructed neuronal network. Stem Cell Rep..

[496] Perrino G, Wilson C, Santorelli M, di Bernardo D (2019). Quantitative characterization of α-synuclein aggregation in living cells through automated microfluidics feedback control. Cell Rep..

[497] de Rus Jacquet A (2023). The contribution of inflammatory astrocytes to BBB impairments in a brain-chip model of Parkinson’s disease. Nat. Commun..

[498] Todd TW, Petrucelli L (2022). Modelling amyotrophic lateral sclerosis in rodents. Nat. Rev. Neurosci..

[499] Cook C, Petrucelli L (2019). Genetic convergence brings clarity to the enigmatic red line in ALS. Neuron.

[500] Hardiman O (2017). Amyotrophic lateral sclerosis. Nat. Rev. Dis. Prim..

[501] Westergard T (2016). Cell-to-cell transmission of dipeptide repeat proteins linked to C9orf72-ALS/FTD. Cell Rep..

[502] Kunze A (2013). Astrocyte–neuron co-culture on microchips based on the model of SOD mutation to mimic ALS. Integr. Biol..

[503] Stoklund Dittlau K (2021). Human motor units in microfluidic devices are impaired by FUS mutations and improved by HDAC6 inhibition. Stem Cell Rep..

[504] Altman T (2021). Axonal TDP-43 condensates drive neuromuscular junction disruption through inhibition of local synthesis of nuclear encoded mitochondrial proteins. Nat. Commun..

[505] Machado CB (2019). In vitro modeling of nerve–muscle connectivity in a compartmentalized tissue culture device. Adv. Biosyst..

[506] Osaki T, Uzel SGM, Kamm RD (2018). Microphysiological 3D model of amyotrophic lateral sclerosis (ALS) from human iPS-derived muscle cells and optogenetic motor neurons. Sci. Adv..

[507] Migazzi A (2021). Huntingtin-mediated axonal transport requires arginine methylation by PRMT6. Cell Rep..

[508] Virlogeux A (2018). Reconstituting corticostriatal network on-a-chip reveals the contribution of the presynaptic compartment to Huntington’s disease. Cell Rep..

[509] Zhao X (2016). TRiC subunits enhance BDNF axonal transport and rescue striatal atrophy in Huntington’s disease. Proc. Natl Acad. Sci. USA.

[510] Virlogeux, A. et al. Increasing brain palmitoylation rescues behavior and neuropathology in Huntington disease mice. *Sci. Adv.*10.1126/sciadv.abb0799 (2021). 10.1126/sciadv.abb0799 PMC8011966 33789888

[511] Maisonneuve BGC (2022). Deposition chamber technology as building blocks for a standardized brain-on-chip framework. Microsyst. Nanoeng..

[512] Han S, Bang S, Kim HN, Choi N, Kim SH (2023). Modulating and monitoring the functionality of corticostriatal circuits using an electrostimulable microfluidic device. Mol. Brain.

[513] Chen X (2021). Modeling sporadic Alzheimer’s disease in human brain organoids under serum exposure. Adv. Sci..

[514] Pavoni S (2018). Small-molecule induction of Aβ-42 peptide production in human cerebral organoids to model Alzheimer’s disease associated phenotypes. PLoS ONE.

[515] Park JC (2021). A logical network-based drug-screening platform for Alzheimer’s disease representing pathological features of human brain organoids. Nat. Commun..

[516] Raja WK (2016). Self-organizing 3D human neural tissue derived from induced pluripotent stem cells recapitulate Alzheimer’s disease phenotypes. PLoS ONE.

[517] Gonzalez C (2018). Modeling amyloid beta and tau pathology in human cerebral organoids. Mol. Psychiatry.

[518] Arber C (2021). Familial Alzheimer’s disease mutations in PSEN1 lead to premature human stem cell neurogenesis. Cell Rep..

[519] Shimada H (2022). A next-generation iPSC-derived forebrain organoid model of tauopathy with tau fibrils by AAV-mediated gene transfer. Cell Rep. Methods.

[520] Yin J, VanDongen AM (2021). Enhanced neuronal activity and asynchronous calcium transients revealed in a 3D organoid model of Alzheimer’s disease. ACS Biomater. Sci. Eng..

[521] Son MY (2017). Distinctive genomic signature of neural and intestinal organoids from familial Parkinson’s disease patient-derived induced pluripotent stem cells. Neuropathol. Appl. Neurobiol..

[522] Smits LM (2019). Modeling Parkinson’s disease in midbrain-like organoids. npj Parkinsons Dis..

[523] Pereira JD (2021). Human sensorimotor organoids derived from healthy and amyotrophic lateral sclerosis stem cells form neuromuscular junctions. Nat. Commun..

[524] Szebényi K (2021). Human ALS/FTD brain organoid slice cultures display distinct early astrocyte and targetable neuronal pathology. Nat. Neurosci..

[525] Zhao J (2020). APOE4 exacerbates synapse loss and neurodegeneration in Alzheimer’s disease patient iPSC-derived cerebral organoids. Nat. Commun..

[526] Marton RM, Pașca SP (2020). Organoid and assembloid technologies for investigating cellular crosstalk in human brain development and disease. Trends Cell Biol..

[527] Miura Y (2022). Engineering brain assembloids to interrogate human neural circuits. Nat. Protoc..

[528] Wang L (2021). A human three-dimensional neural-perivascular ‘assembloid’ promotes astrocytic development and enables modeling of SARS-CoV-2 neuropathology. Nat. Med..

[529] Andersen J (2020). Generation of functional human 3D cortico-motor assembloids. Cell.

[530] Birey F (2022). Dissecting the molecular basis of human interneuron migration in forebrain assembloids from Timothy syndrome. Cell Stem Cell.

[531] Kong D (2023). Cortical-blood vessel assembloids exhibit Alzheimer’s disease phenotypes by activating glia after SARS-CoV-2 infection. Cell Death Discov..

[532] Rickner HD (2022). Single cell transcriptomic profiling of a neuron-astrocyte assembloid tauopathy model. Nat. Commun..

[533] Gordon A (2021). Long-term maturation of human cortical organoids matches key early postnatal transitions. Nat. Neurosci..

[534] Trujillo CA (2019). Complex oscillatory waves emerging from cortical organoids model early human brain network development. Cell Stem Cell.

[535] Mariani J (2015). FOXG1-dependent dysregulation of GABA/glutamate neuron differentiation in autism spectrum disorders. Cell.

[536] Birey F (2017). Assembly of functionally integrated human forebrain spheroids. Nature.

[537] Luo J, Li P (2021). Human pluripotent stem cell-derived brain organoids as in vitro models for studying neural disorders and cancer. Cell Biosci..

[538] Li Y (2017). Induction of expansion and folding in human cerebral organoids. Cell Stem Cell.

[539] Scott G, Huang Y (2022). Engineering cerebral folding in brain organoids. Neural Regen. Res..

[540] Ao Z (2020). One-stop microfluidic assembly of human brain organoids to model prenatal cannabis exposure. Anal. Chem..

[541] Zhu Y (2017). In situ generation of human brain organoids on a micropillar array. Lab Chip.

[542] Qian X (2016). Brain-region-specific organoids using mini-bioreactors for modeling ZIKV exposure. Cell.

[543] Ao Z (2021). Tubular human brain organoids to model microglia-mediated neuroinflammation. Lab Chip.

[544] Karzbrun E, Kshirsagar A, Cohen SR, Hanna JH, Reiner O (2018). Human brain organoids on a chip reveal the physics of folding. Nat. Phys..

[545] Sharma AD (2019). Engineering a 3D functional human peripheral nerve in vitro using the nerve-on-a-chip platform. Sci. Rep..

[546] Sood D (2019). Functional maturation of human neural stem cells in a 3D bioengineered brain model enriched with fetal brain-derived matrix. Sci. Rep..

[547] Zhu Y, Zhang X, Sun L, Wang Y, Zhao Y (2023). Engineering human brain assembloids by microfluidics. Adv. Mater..

[548] Nashimoto Y (2017). Integrating perfusable vascular networks with a three-dimensional tissue in a microfluidic device. Integr. Biol..

[549] Nashimoto Y (2020). Vascularized cancer on a chip: the effect of perfusion on growth and drug delivery of tumor spheroid. Biomaterials.

[550] Ao Z (2022). Understanding immune-driven brain aging by human brain organoid microphysiological analysis platform. Adv. Sci..

[551] Zhang S, Wan Z, Kamm RD (2021). Vascularized organoids on a chip: strategies for engineering organoids with functional vasculature. Lab Chip.

[552] Ayuso JM (2019). Evaluating natural killer cell cytotoxicity against solid tumors using a microfluidic model. Oncoimmunology.

[553] Chen YY (2016). Clarifying intact 3D tissues on a microfluidic chip for high-throughput structural analysis. Proc. Natl Acad. Sci. USA.

[554] Chen B (2019). High-throughput acoustofluidic fabrication of tumor spheroids. Lab Chip.

[555] Misun PM, Rothe J, Schmid YRF, Hierlemann A, Frey O (2016). Multi-analyte biosensor interface for real-time monitoring of 3D microtissue spheroids in hanging-drop networks. Microsyst. Nanoeng..

[556] Sims JR (2023). Donanemab in early symptomatic Alzheimer disease: the TRAILBLAZER-ALZ 2 randomized clinical trial. JAMA.

[557] Cummings JL, Goldman DP, Simmons-Stern NR, Ponton E (2022). The costs of developing treatments for Alzheimer’s disease: a retrospective exploration. Alzheimers Dement..

[558] Akhtar A (2022). Preclinical models for Alzheimer’s disease: past, present, and future approaches. ACS Omega.

[559] Watamura N, Sato K, Saido TC (2022). Mouse models of Alzheimer’s disease for preclinical research. Neurochem. Int..

[560] Vulto P, Joore J (2021). Adoption of organ-on-chip platforms by the pharmaceutical industry. Nat. Rev. Drug Discov..

[561] Marx U (2020). Biology-inspired microphysiological systems to advance patient benefit and animal welfare in drug development. ALTEX.

[562] Wadman M (2023). FDA no longer has to require animal testing for new drugs. Science.

[563] Mastrangeli M, Millet S, van den Eijnden-van Raaij J, Orchid partners, T. (2019). Organ-on-chip in development: towards a roadmap for organs-on-chip. ALTEX.

[564] Figarol A (2020). Interstitial flow regulates in vitro three-dimensional self-organized brain micro-vessels. Biochem. Biophys. Res. Commun..

[565] Mastrangeli M (2019). Building blocks for a European Organ-on-Chip roadmap. ALTEX.

[566] Rusyn I (2022). Microphysiological systems evaluation: experience of TEX-VAL Tissue Chip Testing Consortium. Toxicol. Sci..

[567] Schurdak M (2020). Applications of the microphysiology systems database for experimental ADME-Tox and disease models. Lab Chip.

[568] Sakolish C (2023). Analysis of reproducibility and robustness of a renal proximal tubule microphysiological system OrganoPlate 3-lane 40 for in vitro studies of drug transport and toxicity. Toxicol. Sci..

[569] Nieskens TTG (2021). Nephrotoxic antisense oligonucleotide SPC5001 induces kidney injury biomarkers in a proximal tubule-on-a-chip. Arch. Toxicol..

[570] Santhanam N (2018). Stem cell derived phenotypic human neuromuscular junction model for dose response evaluation of therapeutics. Biomaterials.

[571] Tavakol DN, Fleischer S, Vunjak-Novakovic G (2021). Harnessing organs-on-a-chip to model tissue regeneration. Cell Stem Cell.

[572] Abdelnour C (2022). Perspectives and challenges in patient stratification in Alzheimer’s disease. Alzheimers Res. Ther..

[573] Low LA, Mummery C, Berridge BR, Austin CP, Tagle DA (2021). Organs-on-chips: into the next decade. Nat. Rev. Drug Discov..

[574] Song H-L (2014). β-Amyloid is transmitted via neuronal connections along axonal membranes. Ann. Neurol..

[575] Nobuhara CK (2017). Tau antibody targeting pathological species blocks neuronal uptake and interneuron propagation of tau in vitro. Am. J. Pathol..

[576] Wang B (2018). 14-3-3 proteins reduce cell-to-cell transfer and propagation of pathogenic α-synuclein. J. Neurosci..

[577] Moreno EL (2015). Differentiation of neuroepithelial stem cells into functional dopaminergic neurons in 3D microfluidic cell culture. Lab Chip.

